# Risk estimation and dynamic prediction using discrete-time joint models for longitudinal and multistate data with interval and state censoring

**DOI:** 10.1093/biostatistics/kxag018

**Published:** 2026-07-06

**Authors:** Lu You, Falastin Salami, Carina Törn, Åke Lernmark, Kendra Vehik, Xiang Liu, Peihua Qiu, Roy Tamura

**Affiliations:** Health Informatics Institute, University of South Florida, 3650 Spectrum Blvd, Tampa, FL 33612, United States; Department of Clinical Sciences, Lund University, Jan Waldenströms gata 35, Malmö 21428, Sweden; Department of Clinical Sciences, Lund University, Jan Waldenströms gata 35, Malmö 21428, Sweden; Department of Clinical Sciences, Lund University, Jan Waldenströms gata 35, Malmö 21428, Sweden; Health Informatics Institute, University of South Florida, 3650 Spectrum Blvd, Tampa, FL 33612, United States; Health Informatics Institute, University of South Florida, 3650 Spectrum Blvd, Tampa, FL 33612, United States; Department of Biostatistics, University of Florida, 2004 Mowry Rd, Gainesville, FL 32610,United States; Health Informatics Institute, University of South Florida, 3650 Spectrum Blvd, Tampa, FL 33612, United States

**Keywords:** dynamic prediction, interval censoring, joint modeling, longitudinal data analysis, measurement error, multistate model

## Abstract

This paper presents a joint model of multivariate longitudinal data and multistate data with application to modeling and predicting autoantibody development in The Environmental Determinants of Diabetes in the Young (TEDDY) study. The model quantifies the risks of state transitions based on observed time-varying and non-time-varying risk factors. Based on the estimated model, a dynamic prediction approach is suggested to predict future state occupation probabilities using historical data. The proposed method can handle uncertainties in the observed data, due to measurement errors in the observed longitudinal data and interval censoring or missing information in the observed multistate data. For evaluating the predictions by the proposed approach, some performance metrics and their estimation are discussed. The proposed method is evaluated by some simulation studies. It is discussed in detail how this method can be used in analyzing the TEDDY data by properly handling the missing information and predicting future disease status using the proposed dynamic prediction algorithm.

## 1. Introduction

Many chronic diseases are better understood in terms of their progression through a series of specific events. For example, the progression of heart failure can be described in Stages A to D depending on whether there are symptoms of heart failure and whether there is structural heart disease (c.f., Heidenreich et al. 2022). Alzheimer’s disease can be described by events associated with cognitive decline and functional decline (c.f. Zahodne et al. 2013). Progression of type 1 diabetes (T1D) is characterized by the development of autoantibodies, dysglycemia onset, and the onset of symptoms (c.f., Insel et al. 2015). The timing and the experienced event sequence may vary among individuals, and people experiencing different events may have varied disease risk. Many clinical studies have been designed to understand the pattern of disease progression through these events. Participants of these studies will be monitored for the occurrence of certain events, and biomarkers indicative of disease progression will be recorded over time. Data generated from such studies allow us to examine how various factors contribute to the risks of different events and ultimately influence disease progression.

**Figure 1:**
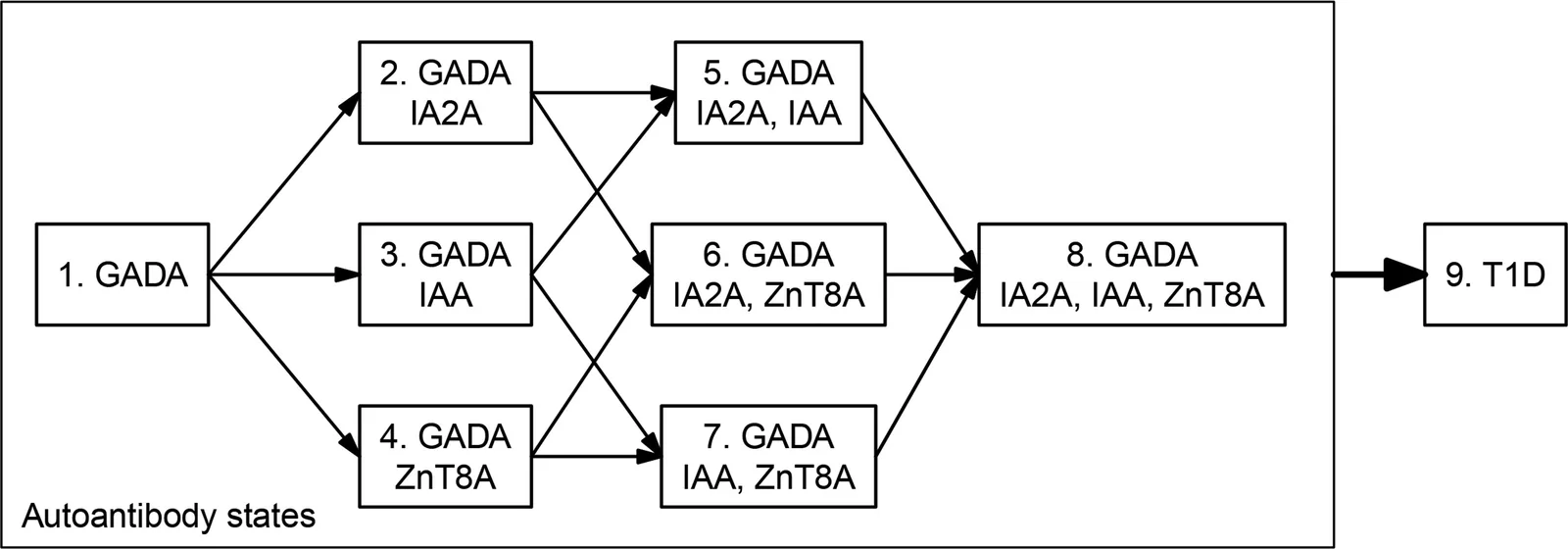
A multistate model for characterizing the pattern of autoantibody development in the TEDDY study.

This research is motivated by The Environmental Determinants of Diabetes in the Young (TEDDY) study. The enrolled participants of the study are genetically at risk of T1D and were followed since 3 until 15 yr of age or diagnosis of T1D (c.f., TEDDY Study Group 2007). T1D is an autoimmune disease characterized by the development of autoantibodies and the destruction of insulin-producing beta cells in the pancreas. Participants were scheduled to be monitored for the development of 4 types of autoantibodies, including glutamic acid decarboxylase autoantibodies (GADA), islet antigen-2 autoantibodies (IA2A), insulin autoantibodies (IAA), and zinc transporter 8 autoantibodies (ZnT8A), every 3 or 6 mo depending on the participant’s age and autoantibody status. Each of these autoantibodies plays a distinct role in the progression to T1D, and there are some patterns of the chronological order of development. For example, IA2A and ZnT8A are typically detected following the detection of other autoantibodies, and the presence of IA2A usually indicates a more advanced stage with higher risks (c.f., Jia and Yu 2023). The majority of current literature describes the development of autoantibodies by the number of detected autoantibodies (usually from a single autoantibody to multiple autoantibodies), or concerns only about the type of the first detected autoantibody (c.f., Giannopoulou et al. 2015; Steck et al. 2015; Krischer et al. 2019, 2022). In this work, we will build on the works by Vehik et al. (2020), Mistry et al. (2023), and Kwon et al. (2022) to investigate the progression and chronological order of autoantibody development considering the specific types. More specifically, we aim to use a multistate model illustrated in Fig. 1 to characterize the pattern of autoantibody development in participants with GADA as the first appearing autoantibody while incorporating longitudinal (eg glucose levels) and non-time-varying covariates (eg demographic information). The model can then be used to make dynamic predictions of risks in developing other autoantibodies and T1D for new individuals following GADA positivity.

The application to the TEDDY dataset reveals a range of new statistical challenges. The observed data may not fully capture the complete event history, and some longitudinal biomarkers are observed with measurement errors. We outline the potential uncertainties in the observed data below and aim to address them through the proposed method. First, the metabolic variables used to measure glucose levels are often observed with measurement errors. The model should enable us to correlate the risk of state transitions when measurement errors are present. Second, the state transition times may be subject to interval censoring when there are missing visits or extended periods without follow-ups. Third, the number of events and the actual event sequence may not be exactly determined for all participants based on the observed data. For example, in Fig. 1, if a participant went from State 1 to State 5 from a visit to the next, we observed that IA2A and IAA were added but were unable to figure out which autoantibody appeared first. Finally, the autoantibody tests are occasionally incomplete. One common case observed in the TEDDY study is related to missing ZnT8A tests, since this autoantibody was assayed retroactively per protocol. As illustrated in Fig. 1, if a participant had a positive GADA test with a missing ZnT8A test during a visit, then we may not be able to determine whether that participant was in State 1 or State 4. This type of uncertainty is referred to as the censored state in other works (c.f., Jackson 2011). Besides these complexities in the observed data, the proposed method will also handle difficulties in making dynamic predictions of disease risk from the estimated model and in evaluating the predictions. In addition, several performance metrics for evaluating the dynamic disease risk predictions will be presented along with their estimation from interval-censored data.

There are some relevant statistical methods in the literature that can be used to address some challenges mentioned above. For instance, some existing studies have used joint modeling of longitudinal and multistate data to explore the relationship between longitudinal variables and the risk of state transitions (c.f., Lange et al. 2015; Ferrer et al. 2016; Yiu and Tom 2017). But, these methods typically do not address interval-censored multistate data or censored states. While there are existing methods for analyzing interval-censored multistate data using parametric or semiparametric models, they typically do not focus on longitudinal data (c.f., Marshall et al. 1995; Pak et al. 2017; Zhang et al. 2020). In cases when only longitudinal disease risk factors are available, different dynamic screening systems have been developed for dynamic disease screening under various conditions (c.f., Qiu and Xiang 2014; You and Qiu 2020; Qiu and You 2022; Qiu 2024). These methods, however, cannot accommodate multistate data at all. Dynamic disease predictions based on joint models with multiple events have been discussed previously by Blanche et al. (2015) and Proust-Lima et al. (2016). But, such research is mainly limited to competing risk modeling. Therefore, new methodologies are needed to analyze the TEDDY dataproperly.

To fill the methodological gap mentioned above, we develop a joint modeling approach for longitudinal and multistate data. Our method primarily integrates techniques from Ferrer et al. (2016), Gu et al. (2024), and You et al. (2024a). The method by Ferrer et al. (2016) outlines the model estimation framework, though it does not account for interval-censored data. Building on this, Gu et al. (2024) proposed a method for handling interval-censored multistate models with random effects using data augmentation. However, this work does not directly focus on joint modeling, and the method may be computationally demanding for multistate models with complex structures while also imposing additional assumptions (eg no loops in the multistate model). Our proposed approach combines these foundational efforts with the discrete approximation method from You et al. (2024a) to enable efficient computation. Additionally, we will discuss methods for evaluating the predictions of future state occupation probabilities in multistate models. These methods build on evaluation metrics from Blanche et al. (2015), which address joint models of longitudinal and competing-risks data, and Beyene and El Ghouch (2022), which focus on interval-censored single-event data. However, extending these metrics to multistate models with interval-censoring presents significant challenges.

The remainder of the manuscript is organized as follows. The proposed method is introduced in Section 2, where we will provide details about model construction and estimation and about dynamic disease risk prediction. The proposed method will be evaluated in Section 3 by some simulation studies. In Section 4, application of the proposed method to a dataset from the TEDDY study is discussed in detail. Section 5 concludes the paper with some discussions and directions for future research.

## 2. Materials and methods

### 2.1. Model and notation

#### 2.1.1. Study setting and notation

Before introducing the proposed method, we provide an overview of the notations utilized to describe the observed data. Suppose that the dataset includes longitudinal data and multistate data from * m * individuals. In the multistate data, there are $ N_{s} $ states under investigation, labeled from 1 to $ N_{s} $. Transitions between different states can be described by a directed graph $ (\mathbb{V},\mathbb{E}) $, where the set of vertices $ \mathbb{V}=\{1 , \ldots, N_{s}\} $ represents the $ N_{s} $ states and the collection of directed edges $ \mathbb{E} $ is defined such that $ (s_{1},s_{2})\in\mathbb{E} $ if and only if a transition from state $ s_{1} $ to state $ s_{2} $ is allowed for $ s_{1}\neq s_{2}\in\mathbb{V} $. Let $ s_{i}(t) $ denote the true state that the * i *th individual occupies at time * t *. Due to interval censoring, the complete history of $ s_{i}(t) $ is not directly observed. The * i *th individual is sequentially monitored for state transitions at the observation time sequence $ 0\,=\,T_{i0} < T_{i1} < T_{i2} < \ldots < T_{in_{i}} $, where $ T_{in_{i}} $ can possibly indicate a truncation of the observation time sequence due to right censoring. In this manuscript, we allow states to be observed with uncertainty. Specifically, at each monitoring time $ T_{ij} $, we only know that $ s_{i}(t) $ lies within the set $ S_{ij}\subset\mathbb{V} $. In the literature, such partially observed states are often referred to as censored states (c.f., Jackson 2011). For example, in Fig. 1, when we are not sure if the individual * i * is in State 1 or 4, $ S_{ij}=\{1,4\} $. We define $ \delta_{i, s_{1}\rightarrow s_{2}}(t)=I\big(s_{i}(t-)=s_{1},s_{i}(t)=s_{2}\big) $ and $ g_{i, s_{1}}(t)=I\big(s_{i}(t-)=s_{1}\big) $ as the underlying event indicator process and at-risk process, respectively. Namely, $ \delta_{i, s_{1}\rightarrow s_{2}}(t)=1 $ if and only if the * i *th individual transitions from state $ s_{1} $ to $ s_{2} $ at time * t *, and $ g_{i, s_{1}}(t)=1 $ if and only if the * i *th individual is at risk of transitioning out of state $ s_{1} $ at time * t *.

The longitudinal dataset includes $ N_{y} $ repeatedly measured risk factors of transitions for the * m * individuals. The * j *th measurement of the * k *th longitudinal variable for the * i *th individual, denoted by $ y_{ikj} $, is collected at time $ t_{ikj} $ ($ i\,=\,1\ldots, N $, $ k\,=\,1 , \ldots, N_{y} $ and $ j\,=\,1 , \ldots, n_{ik} $). The presence of uncertainty in longitudinal data implies that the longitudinal variables $ y_{ikj} $ are observed with measurement errors. Namely, it is assumed that $ y_{ikj}=m_{ik}(t_{ikj})+\epsilon_{ikj} $, where $ m_{ik}(t) $ denotes the latent trajectory of the * k *th longitudinal variable and $ \epsilon_{ikj} $ is the mean-zero measurement error. Our proposed joint model aims to describe risk factors that are potentially related to state transitions. It is believed that the risks of state transitions are associated with the trajectories $ m_{ik}(t) $ whereas the measurement error $ \epsilon_{ikj} $ is noninformative of the state transition process. In addition to the longitudinal variables, the model will incorporate $ N_{z} $ non-time-varying risk factors, denoted by a $ N_{z} $-dimensional vector $ \mathbf{z}_{i} $ for the * i *th individual, which are observed without measurement errors. To simplify the notations, we stack all risk factors under investigation as a $ N_{\eta} $-dimensional vector $ \boldsymbol{\eta}_{i}(t)=(\mathbf{m}_{i}(t)^{\mathsf{T}},\mathbf{z}_{i}^{\mathsf{T}})^ {\mathsf{T}} $, where $ \mathbf{m}_{i}(t)=(m_{i1}(t),\ldots, m_{iN_{y}}(t))^{\mathsf{T}} $ and $ N_{\eta}=N_{y}+N_{z} $. We let $ [0 , \mathcal{T}] $ be the design time interval, where $ \mathcal{T} $ is chosen to include all observation times $ \{T_{ij}\}_{i\,=\,1}^{N}{}_{j\,=\,1}^{n_{i}} $ and $ \{t_{ikj}\}_{i\,=\,1}^{N}{}_{k\,=\,1}^{N_{y}}{}_{j\,=\,1}^{n_{ik}} $. In this manuscript, we will assume that the visiting processes $ \{T_{ij}\}_{i\,=\,1}^{N}{}_{j\,=\,1}^{n_{i}} $ and $ \{t_{ikj}\}_{i\,=\,1}^{N}{}_{k\,=\,1}^{N_{y}}{}_{j\,=\,1}^{n_{ik}} $ follow the “visiting completely at random” assumption in Pullenayegum and Lim (2016) (detailed in Section S1.3).

#### 2.1.2. Discrete-time approximation framework

In this paper, the discrete-time approximation considered in You et al. (2024a) is used to simplify model estimation. Rather than assuming that event times have discrete probability masses at specific time points as in other discrete-time survival models (c.f., Section 12.2 of Singer and Willett 2003), we approximate an underlying continuous-time process by formulating the model over intervals. The discrete-time approximation method partitions the interval $ (0 , \mathcal{T}] $ into $ N_{t} $ disjoint subintervals $ \mathfrak{I}_{1}=(\tau_{0},\tau_{1}],\mathfrak{I}_{2}=(\tau_{1},\tau_{2}],\ldots , \mathfrak{I}_{N_{t}}=(\tau_{N_{t}-1},\tau_{N_{t}}] $. The full assumptions on the partition are discussed in detail in Section S1.4. Here, we assume that (i) $ \mathbb{T}=\{\tau_{0},\ldots , \tau_{N_{t}}\} $ encompasses observation times $ \{T_{ij}\}_{i\,=\,1}^{N}{}_{j\,=\,1}^{n_{i}} $, (ii) the longitudinal trajectories $ \mathbf{m}_{i}(t) $ can be adequately approximated by a constant in $ \mathfrak{I}_{j} $, and (iii) the likelihood of making 2 or more transitions in $ \mathfrak{I}_{j} $ is negligibly small. Note that Requirement (i) is trivial, and Requirement (ii) and (iii) can also be achieved by adding more elements to $ \mathbb{T} $ to reduce $ \max_{j\in 1\ldots, N_{t}}|\tau_{j}-\tau_{j-1}| $ with additional assumptions on $ \mathbf{m}_{i}(t) $ and the transition rates (eg Lipschitz continuity). For each $ j\,=\,1\ldots N_{t} $, let us define $ \delta_{i, s_{1}\rightarrow s_{2}}(\mathfrak{I}_{j})=\max_{t\in\mathfrak{I}_{j}}\delta_{i, s_{1}\rightarrow s_{2}}(t) $ and $ g_{i, s_{1}}(\mathfrak{I}_{j})=\max_{t\in\mathfrak{I}_{j}}g_{i, s_{1}}(t) $, so that $ \delta_{i, s_{1}\rightarrow s_{2}}(\mathfrak{I}_{j})=1 $ if and only if the transition from state $ s_{1} $ to state $ s_{2} $ is observed in the interval $ \mathfrak{I}_{j} $, and $ g_{i, s_{1}}(\mathfrak{I}_{j})=1 $ if and only if the individual is at risk of transitioning out of state $ s_{1} $ before entering the interval $ \mathfrak{I}_{j} $. Similarly, we let $ s_{i}(\mathfrak{I}_{j})=s_{i}(\tau_{j}) $ be the state occupied at the end of the interval $ \mathfrak{I}_{j} $. Additionally, $ \mathbf{m}_{i}(\mathfrak{I}_{j}) $ and $ \boldsymbol{\eta}_{i}(\mathfrak{I}_{j}) $ are defined as $ \mathbf{m}_{i}(\tilde{\tau}_{j}) $ and $ \boldsymbol{\eta}_{i}(\tilde{\tau}_{j}) $, respectively, where $ \tilde{\tau}_{j}=(\tau_{j}+\tau_{j-1})/2 $ is the mid-point of the interval $ \mathfrak{I}_{j} $.

#### 2.1.3. Longitudinal submodel

The proposed joint model is an integration of a longitudinal model and a survival model.The detailed assumptions underlying the proposed joint model are provided in Section S1. The longitudinal model seeks to reconstruct the trajectory $ \mathbf{m}_{i}(t) $ from the observed data. We assume that the trajectory of the * k *th longitudinal variable $ m_{ik}(t) $ can be approximated by a linear combination of $ N_{Bk} $ spline basis functions $ B_{k1}(t),\ldots, B_{kN_{Bk}}(t) $:


\begin{align*} m_{ik}(t)=\sum\limits_{l=1}^{N_{Bk}}b_{ikl}B_{kl}(t)=\mathbf{b}_{ik}^{\mathsf{T}}\mathbf{B}_{k}(t),\end{align*}

where $ \mathbf{b}_{ik}=(b_{ik1},\ldots, b_{ikN_{Bk}})^{\mathsf{T}} $ is the coefficient vector and $ \mathbf{B}_{k}(t)=(B_{k1}(t),\ldots, B_{kN_{Bk}}(t))^{\mathsf{T}} $. The spline basis functions can be selected from a range of different types, including linear splines, polynomial splines, and B-splines. The observed longitudinal data can then be modeled by the following mixed effects model (c.f., Rice and Wu 2001):


\begin{align*}\mathbf{y}_{ik}=\mathbf{X}_{ik}\mathbf{b}_{ik}+\boldsymbol{\epsilon}_{ik}=\mathbf{X}_{ik}\mathbf{c}_{k}+\mathbf{X}_{ik}\mathbf{a}_{ik}+\boldsymbol{\epsilon}_{ik},\end{align*}

where $ \mathbf{y}_{ik}=(y_{ik1},\ldots, y_{ikn_{ik}})^{\mathsf{T}} $ is the vector of observed longitudinal data, $ \mathbf{c}_{k}=(c_{k1},\ldots, c_{kN_{Bk}})^{\mathsf{T}} $ is the fixed-effects coefficient vector, $ \mathbf{a}_{ik}=(a_{k1},\ldots, a_{kN_{Bk}})^{\mathsf{T}} $ is the zero-mean random-effects coefficient vector describing individual deviations from the population mean function, $ \boldsymbol{\epsilon}_{ik}=(\epsilon_{ik1},\ldots , \epsilon_{ikN_{Bk}})^{\mathsf{T}} $ is the vector of zero-mean measurement errors, and


\begin{align*}\mathbf{X}_{ik}=\begin{bmatrix}B_{k1}(t_{ik1})&\cdots&B_{kN_{Bk}} (t_{ik1})\\\vdots&\ddots&\vdots\\B_{k1}(t_{ikn_{ik}})&\cdots&B_{kN_{Bk}}(t_{ikn_{ik}})\end{bmatrix}\end{align*}

is the design matrix for modeling $ \mathbf{y}_{ik} $. The measurement errors $ \epsilon_{ikj} $ are assumed to be independently distributed with the normal distribution $ N(0 , \sigma_{k}^{2}) $. To accommodate within-subject data correlation, it is assumed that $ \big(\mathbf{a}_{i1}^{\mathsf{T}},\ldots , \mathbf{a}_{iN_{y}}^{\mathsf{T}}\big)^{\mathsf{T}} $ follows a joint multivariate normal distribution $ N(\boldsymbol{0},\boldsymbol{\Sigma}_{a}) $. To simplify the notations, let $ \mathbf{a}_{i}=\big(\mathbf{a}_{i1}^{\mathsf{T}},\ldots , \mathbf{a}_{iN_{y}}^ {\mathsf{T}}\big)^{\mathsf{T}} $, $ \mathbf{b}_{i}=\big(\mathbf{b}_{i1}^{\mathsf{T}},\ldots , \mathbf{b}_{iN_{y}}^ {\mathsf{T}}\big)^{\mathsf{T}} $, and $ \mathbf{c}=\big(\mathbf{c}_{1}^{\mathsf{T}},\ldots , \mathbf{c}_{N_{y}}^{\mathsf{T}}\big)^{\mathsf{T}} $ be $ N_{a}=\sum_{k\,=\,1}^{N_{y}}N_{Bk} $ dimensional vectors. A more complete description of the assumptions in the longitudinal model is provided in Section S1.1.

#### 2.1.4. Multistate submodel

The multistate model aims to identify the associations between the state transitions and the longitudinal trajectories and risk factors. Let $ \boldsymbol{\eta}_{i}(t)=(\mathbf{m}_{i}(t),\mathbf{z}_{i})^{\mathsf{T}} $ be the collection of all risk factors from the * i *th individual. For each transition $ (s_{1},s_{2})\in\mathbb{E} $, we aim to quantify the transition rate in relation to $ \boldsymbol{\eta}_{i}(t) $, or more generally, a transformation of $ \boldsymbol{\eta}_{i}(t) $ that is applied commonly to all individuals, which we denote by $ \boldsymbol{\eta}_{i, s_{1}\rightarrow s_{2}}(t) $. In this manuscript, we primarily consider current value joint models in which $ \boldsymbol{\eta}_{i, s_{1}\rightarrow s_{2}}(t) $ is either the full vector $ \boldsymbol{\eta}_{i}(t) $ or a subset of its components, and we similarly define $ \boldsymbol{\eta}_{i, s_{1}\rightarrow s_{2}}(\mathfrak{I}_{j}) $ as the corresponding subset of $ \boldsymbol{\eta}_{i}(\mathfrak{I}_{j}) $. The extensions to other cases are fully discussed in Rizopoulos and Ghosh (2011). Let $ N_{\eta, s_{1}\rightarrow s_{2}} $ denote the number of elements in $ \boldsymbol{\eta}_{i, s_{1}\rightarrow s_{2}}(t) $. The following multistate model with a complementary log-log link is considered


\begin{align*}\operatorname{P}\Big(\delta_{i, s_{1}\rightarrow s_{2}}(\mathfrak{I}_{j})=1\Big|g_{i, s_{1}}(\mathfrak{I}_{j})=1\Big)=1-\exp\Big\{-h_{0, s_{1}\rightarrow s_{2}}(\mathfrak{I}_{j})\exp\big[\boldsymbol{\beta}_{s_{1}\rightarrow s_ {2}}^{\mathsf{T}}\boldsymbol{\eta}_{i, s_{1}\rightarrow s_{2}}(\mathfrak{I}_{j})\big]\Big\},\end{align*}

where $ \boldsymbol{\beta}_{s_{1}\rightarrow s_{2}} $ is a $ N_{\eta, s_{1}\rightarrow s_{2}} $-dimensional vector of regression coefficients that measure the association between risk factors and the risk of transitions from $ s_{1} $ to $ s_{2} $, and the parameter $ h_{0, s_{1}\rightarrow s_{2}}(\mathfrak{I}_{j}) $ characterizes the baseline transition rate in the interval $ \mathfrak{I}_{j} $. When the focus of statistical inference is on the regression coefficient $ \boldsymbol{\beta}_{s_{1}\rightarrow s_{2}} $, $ \mathbf{h}_{0, s_{1}\rightarrow s_{2}}=\big\{h_{0, s_{1}\rightarrow s_{2}}(\mathfrak{I}_{j})\big\}_{j\,=\,1}^{N_{t}} $ are usually treated as nuisance parameters. As demonstrated in You et al. (2024a), the discrete-time model is an approximation of a continuous-time proportional hazards model specified by


\begin{align*}\lim_{\Delta t\to 0+}P\big(s_{i}(t+\Delta t)=s_{2}|s_{i}(t)=s_{1}\big)/\Delta t=h_{0, s_{1}\rightarrow s_{2}}(t)\exp\big\{\boldsymbol{\beta}_{s_{1}\rightarrow s_{2}}^{\mathsf{T}}\boldsymbol{\eta}_{i, s_{1}\rightarrow s_{2}}(t)\big\},\end{align*}

where the continuous-time baseline transition rate $ h_{0, s_{1}\rightarrow s_{2}}(t) $ can be related to discrete-time baseline transition rate $ h_{0, s_{1}\rightarrow s_{2}}(\mathfrak{I}_{j}) $ through the approximation $ h_{0, s_{1}\rightarrow s_{2}}(\mathfrak{I}_{j})\approx(\tau_{j}-\tau_{j-1})h_{0 ,s_{1}\rightarrow s_{2}}(t) $ (see Section S1.4). In the current formulation, the transition probability $ \operatorname{P}\big(\delta_{i, s_{1}\rightarrow s_{2}}(\mathfrak{I}_{j})=1\big|g_{i, s_{1}}(\mathfrak{I}_{j})=1\big) $ is directly associated with the value of $ \boldsymbol{\eta}_{i, s_{1}\rightarrow s_{2}}(\mathfrak{I}_{j}) $, such a case is usually referred to as the current value parameterization (c.f., Rizopoulos and Ghosh 2011). A more complete description of the assumptions in the multistate model is provided in Section S1.2.

### 2.2. Model estimation

Estimation of multistate models with mixed-effects terms has been previously discussed by Gu et al. (2024) and Ferrer et al. (2016). In particular, Gu et al. (2024) addressed interval censoring in a mixed effects model, while Ferrer et al. (2016) focused on joint models incorporating a longitudinal component. Our current manuscript expands their methods to allow censored states. As the estimation of the model considered in our manuscript is usually computationally intensive, to simplify the procedure, we further adopt the approximation method proposed by You et al. (2024a) in this manuscript.

Let $ \mathcal{S}_{i}=\big\{(S_{ij},T_{ij}):j\,=\,1 , \ldots, n_{i}\big\} $ denote all observed state occupation data for the * i *th individual, $ \mathcal{Y}_{i}=\big\{(y_{ikj},t_{ikj}):k\,=\,1 , \ldots, N_{y};\, j\,=\,1 , \ldots, n_{ik}\big\} $ denote all observed longitudinal data for the * i *th individual, $ \mathcal{A}_{i}=\big\{\big(g_{i, s_{1}}(\mathfrak{I}_{j}),\delta_{i, s_{1}\rightarrow s_{2}}(\mathfrak{I}_{j})\big):(s_{1},s_{2})\in\mathbb{E};\, j\,=\,1 , \ldots, N_{t}\big\} $ be the augmented data of state occupation, $ \boldsymbol{\Theta}_{\beta, h}=\big\{(\boldsymbol{\beta}_{s_{1}\rightarrow s_{2}},h_{0, s_{1}\rightarrow s_{2}}(\mathfrak{I}_{j})):(s_{1},s_{2})\in\mathbb{E};\, j\,=\,1 , \ldots, N_{t}\big\} $ denote all parameters in the multistate model, $ \boldsymbol{\Theta}_{\sigma}=\{\sigma_{k}^{2}:k\,=\,1 , \ldots, N_{y}\} $ denote the parameters in the longitudinal model, and $ \boldsymbol{\Theta}_{c , \Sigma}=\{\mathbf{c},\boldsymbol{\Sigma}_{a}\} $ be the parameters in the mixed-effects model. Additionally, $ \boldsymbol{\Omega}_{\mathbf{b}_{i}} $ and $ \boldsymbol{\Omega}_{\mathcal{A}_{i}} $ denote the sample spaces for $ \mathbf{b}_{i} $ and $ \mathcal{A}_{i} $. In our model, $ \mathcal{S}_{i} $ and $ \mathcal{Y}_{i} $ represent all observed information, while $ \mathbf{b}_{i} $ and $ \mathcal{A}_{i} $ are unobserved latent quantities. We will use the notation $ [\![\mathbf{v}]\!]_{\rho} $ to denote the subview of a vector $ \mathbf{v} $ corresponding to the indices in the set * ρ *, and $ [\![\mathbf{A}]\!]_{\rho_{1},\rho_{2}} $ to denote the subview of a matrix $ \mathbf{A} $ corresponding to the row indices $ \rho_{1} $ and column indices $ \rho_{2} $.

The likelihood function of the joint model is an integration of the probability density/mass functions of multistate data, longitudinal data, and mixed-effects terms


\begin{align*} L(\boldsymbol{\Theta}_{\sigma},\boldsymbol{\Theta}_{\beta, h},\boldsymbol{\Theta}_{c , \Sigma})=\prod\limits_{i=1}^{N}\int_{\Omega_{\mathbf{b}_{i}}}f\big(\mathcal{S}_{i}\big|\mathbf{b}_{i};\boldsymbol{\Theta}_{\beta, h}\big)f\big(\mathcal{Y}_{i}\big{|}\mathbf{b}_{i};\boldsymbol{\Theta}_{\sigma}\big)f\big(\mathbf{b}_{i}\big|\boldsymbol{\Theta}_{c , \Sigma}\big)\, d\mathbf{b}_{i}.\end{align*}

By the model assumptions (see Section S1) and the standard derivation of joint models (see Ferrer et al. 2016), the log probability density function for the longitudinal data and the mixed-effects terms $ \mathbf{b}_{i} $ can be readily written as follows:


\begin{align*}\log\, f(\mathcal{Y}_{i}|\mathbf{b}_{i};\boldsymbol{\Theta}_{\sigma}) &=\sum_{k=1}^{N_{y}}\bigg[-\frac{1}{2\sigma_{k}^{2}}(\mathbf{y}_ {ik}-\mathbf{X}_{ik}\mathbf{b}_{ik})^{\mathsf{T}}(\mathbf{y}_{ik}-\mathbf{X}_{ik}\mathbf{b}_{ik})-\frac{n_{ik}}{2}\log\{2\pi\sigma_{k}^{2}\}\bigg]\\\log\, f(\mathbf{b}_{i}|\boldsymbol{\Theta}_{c , \Sigma}) &=-\frac{1}{2}(\mathbf{b}_{i}-\mathbf{c})^{\mathsf{T}}\boldsymbol{\Sigma}_ {a}^{-1}(\mathbf{b}_{i}-\mathbf{c})-\frac{1}{2}\log\det\{2\pi\boldsymbol{\Sigma}_{a}\}.\end{align*}

Unfortunately, the log probability mass function for the multistate data takes the complicated form below


(1) \begin{align*}\log\, f(\mathcal{S}_{i}|\mathbf{b}_{i};\boldsymbol{\Theta}_{\beta, h})=\log\bigg\{[\![\boldsymbol{1}]\!]_{S_{i0}}^{\mathsf{T}}\bigg[\prod\limits_{j=1}^{n_{i}}\big[\![\mathbf{P}_{i ,(T_{i, j-1},T_{ij}]}(\mathbf{b}_{i};\boldsymbol{\Theta}_{\beta, h})\big]\!]_{S_{i, j-1}, S_{ij}}\bigg][\![\boldsymbol{1}]\!]_{S_{in_{i}}}\bigg\},\end{align*}

where $ \boldsymbol{1} $ is the 1-vector of size $ N_{s} $, * ∏ * denotes matrix multiplication defaulting to left-to-right order, the $ (s_{1},s_{2}) $-th entry of $ \mathbf{P}_{i , \mathfrak{I}_{j}}(\mathbf{b}_{i};\boldsymbol{\Theta}_{\beta, h}) $ is


(2) \begin{align*} [\![\mathbf{P}_{i , \mathfrak{I}_{j}}(\mathbf{b}_{i};\boldsymbol{\Theta}_{\beta, h})]\!]_{\{s _{1}\},\{s_{2}\}}=\begin{cases}\operatorname{P}\Big(\delta_{i, s_{1}\rightarrow s_{2}}(\mathfrak{I}_{j})=1\Big|g_{i, s_{1}}(\mathfrak{I}_{j})=1\Big)&s_{1}\neq s_{2}\\1-\sum\nolimits_{s_{2}:s_{2}\neq s_{1}}\operatorname{P}\Big(\delta_{i, s_{1}\rightarrow s_{2}}(\mathfrak{I}_{j})=1\Big|g_{i, s_{1}}(\mathfrak{I}_{j})=1\Big)&s_{1}=s_{2}\end{cases},\end{align*}

and $ \mathbf{P}_{i,(t_{1},t_{2}]}(\mathbf{b}_{i};\boldsymbol{\Theta}_{\beta, h})=\prod_{\mathfrak{I}_{j}\subset(t_{1},t_{2}]}\mathbf{P}_{i , \mathfrak{I}_{j}}(\mathbf{b}_{i};\boldsymbol{\Theta}_{\beta, h}) $. The matrix multiplications in (1) make it challenging to differentiate the likelihood function for parameter estimation. To facilitate a simple and computationally efficient model estimation, You et al. (2024a) proposed to use the following log probability mass function $ \log\, f(\mathcal{A}_{i},\mathcal{S}_{i}|\mathbf{b}_{i};\boldsymbol{\Theta}_{\beta, h}) $ for the augmented data $ \mathcal{A}_{i},\mathcal{S}_{i} $, and apply a first-order approximation $ \log\, f^{(1)}(\mathcal{A}_{i},\mathcal{S}_{i}|\mathbf{b}_{i};\boldsymbol{\Theta}_{\beta ,h}) $


\begin{align*} &\log\, f(\mathcal{A}_{i},\mathcal{S}_{i}|\mathbf{b}_{i};\boldsymbol{\Theta} _{\beta, h})=\log\, f(\mathcal{A}_{i}|\mathbf{b}_{i};\boldsymbol{\Theta}_{\beta, h})+\log\, f (\mathcal{S}_{i}|\mathcal{A}_{i},\mathbf{b}_{i})=\\ &\quad\sum\limits_{j=1}^{N_{t}}\sum\limits_{(s_{1},s_{2})\in\mathbb{E}}g_{i, s_{1}}(\mathfrak{I}_{j})\bigg[\delta_{i, s_{1}\rightarrow s_{2}}(\mathfrak{I}_{j})\log\Big\{\exp\big\{h_{0, s_{1}\rightarrow s_{2}}(\mathfrak{I}_{j})\exp\big(\boldsymbol{\beta}_{s_{1}\rightarrow s_{2}}^{\mathsf{T}}\boldsymbol{\eta}_{i, s_{1}\rightarrow s_{2}}(\mathfrak{I}_{j})\big)\big\}-1\Big\}\\&\qquad-h_{0, s_{1}\rightarrow s_{2}}(\mathfrak{I}_{j})\exp\big\{\boldsymbol{\beta}_{s_{1}\rightarrow s_{2}}^{\mathsf{T}}\boldsymbol{\eta}_{i, s_{1}\rightarrow s _{2}}(\mathfrak{I}_{j})\big\}\bigg]+\log\, f(\mathcal{S}_{i}|\mathcal{A}_{i} ,\mathbf{b}_{i}),\\&\log\, f^{(1)}(\mathcal{A}_{i},\mathcal{S}_{i}|\mathbf{b}_{i};\boldsymbol{\Theta}_{\beta, h})=\\&\quad\sum\limits_{j=1}^{N_{t}}\sum\limits_{(s_{1},s_{2})\in\mathbb{E}}g_{i, s_{1}}(\mathfrak{I}_{j})\bigg[\delta_{i, s_{1}\rightarrow s_{2}}(\mathfrak{I}_{j})\big\{\log h_{0, s_{1}\rightarrow s_{2}}(\mathfrak{I}_{j})+\boldsymbol{\beta}_{s_{1}\rightarrow s_{2}}^{\mathsf{T}}\boldsymbol{\eta}_{i, s_{1}\rightarrow s_{2}}(\mathfrak{I}_{j})\big\}\\&\qquad-h_{0, s_{1}\rightarrow s_{2}}(\mathfrak{I}_{j})\big(1-\delta_{i, s_{1}\rightarrow s_{2}}(\mathfrak{I}_{j})/2\big)\exp\big\{\boldsymbol{\beta}_{s_{1}\rightarrow s_{2}}^{\mathsf{T}}\boldsymbol{\eta}_{i, s_{1}\rightarrow s_{2}}(\mathfrak{I}_{j})\big\}\bigg]+\log\, f(\mathcal{S}_{i}|\mathcal{A}_{i},\mathbf{b}_{i}).\end{align*}

where $ f(\mathcal{S}_{i}|\mathcal{A}_{i},\mathbf{b}_{i}) $ should not depend on the parameters $ \boldsymbol{\Theta}_{\sigma},\boldsymbol{\Theta}_{\beta, h},\boldsymbol{\Theta}_{c , \Sigma} $ by the noninformative censored state assumption. By the proposed approximation and data augmentation technique, the likelihood function will become


\begin{align*} L^{(1)}(\boldsymbol{\Theta}_{\sigma},\boldsymbol{\Theta}_{\beta, h},\boldsymbol{\Theta}_{c , \Sigma})=\prod\limits_{i=1}^{N}\int_{\boldsymbol{\Omega}_{\mathbf{b}_{i}}}\int_{\boldsymbol{\Omega} _{\mathcal{A}_{i}}}f^{(1)}\big(\mathcal{A}_{i},\mathcal{S}_{i}\big|\mathbf {b}_{i};\boldsymbol{\Theta}_{\beta, h}\big)f\big(\mathcal{Y}_{i}\big|\mathbf{b}_{i};\boldsymbol{\Theta}_{\sigma}\big)f\big(\mathbf{b}_{i}\big|\boldsymbol{\Theta}_{c , \Sigma}\big)\, d\mathcal{A}_{i}d\mathbf{b}_{i}.\end{align*}

Let $ \tilde{\operatorname{E}}_{i}[\cdot]=\operatorname{E}[\cdot|\mathcal{S}_{i},\mathcal{Y}_{i}] $ be the conditional expectation of random variables given the observed data from the * i *th individual $ \mathcal{S}_{i} $ and $ \mathcal{Y}_{i} $, and $ \tilde{\operatorname{E}}[\cdot]=\operatorname{E}\big[\cdot\big|\{\mathcal{S}_{i}\}_{i\,=\,1}^{N},\{\mathcal{Y}_{i}\}_{i\,=\,1}^{N}\big] $ be the conditional expectation of random variables given all observed data. The expectation-maximization (EM) algorithm proceeds by repeatedly maximizing $ \tilde{\operatorname{E}}[l_{c}^{(1)}(\boldsymbol{\Theta}_{\sigma},\boldsymbol{\Theta}_{\beta, h},\boldsymbol{\Theta}_{c , \Sigma})] $, where $ l_{c}^{(1)} $ is the complete log-likelihood function given below:


(3) \begin{align*} l_{c}^{(1)}(\boldsymbol{\Theta}_{\sigma},\boldsymbol{\Theta}_{\beta, h},\boldsymbol{\Theta}_{c , \Sigma}) =\sum\limits_{i=1}^{N}\Big[\log\, f^{(1)}\big(\mathcal{A}_{i},\mathcal{S}_{i}\big{|}\mathbf{b}_{i};\boldsymbol{\Theta}_{\beta, h}\big)+\log\, f\big(\mathcal{Y}_{i}\big{|}\mathbf{b}_{i};\boldsymbol{\Theta}_{\sigma}\big)+\log\, f\big(\mathbf{b}_{i}\big{|}\boldsymbol{\Theta}_{c , \Sigma}\big)\Big].\end{align*}

By differentiating (3) with respect to the parameters, we obtain the following parameter updating procedure:


\begin{align*}\begin{gathered}\mathbf{c}\leftarrow\sum_{i=1}^{N}\tilde{\operatorname{E}}_{i}[\mathbf{b}_{i}]/N , \;\boldsymbol{\Sigma}_{a}\leftarrow\sum_{i=1}^ {N}\tilde{\operatorname{E}}_{i}[(\mathbf{b}_{i}-\mathbf{c})^{\otimes 2}]/N , \;\sigma_{k}^{2}\leftarrow\sum_{i=1}^{N}\sum_{j=1}^{n_{ik}}\tilde{\operatorname{E}}_{i}[(y_{ikj}-\mathbf{b}_{ik}^{\mathsf{T}}\mathbf{B}_{k}(t_{ikj}))^{2}]/\sum_{i=1}^{N}n_{ik}\\ \boldsymbol{\beta}_{s_{1}\rightarrow s_{2}}\leftarrow\boldsymbol{\beta}_{s_{1}\rightarrow s_{2}}-\bigg[\frac{\partial^{2}\tilde{\operatorname{E}}[l_{c}^{(1)}]}{(\partial\boldsymbol{\beta}_{s_{1}\rightarrow s_{2}})^{2}}\bigg]^{-1}\bigg[\frac{\partial\tilde{\operatorname{E}}[l_{c}^{(1)}]}{\partial\boldsymbol{\beta}_{s_{1}\rightarrow s_{2}}}\bigg],\; h_{0, s_{1}\rightarrow s_{2}}(\mathfrak{I}_{j})\leftarrow A_{s_{1}\rightarrow s_{2}}^{0}(\mathfrak{I}_{j})/A_{s_{1}\rightarrow s_{2}}^{1}(\mathfrak{I}_{j}),\end{gathered}\end{align*}

where $ A_{s_{1}\rightarrow s_{2}}^{0}(\mathfrak{I}_{j})=\sum_{i\,=\,1}^{N}\tilde{\operatorname{E}}_{i}[g_{i, s_{1}}(\mathfrak{I}_{j})\delta_{i, s_{1}\rightarrow s _{2}}(\mathfrak{I}_{j})] $, and


\begin{align*} A_{s_{1}\rightarrow s_{2}}^{1}(\mathfrak{I}_{j}) =\sum\limits_{i=1}^{N}\tilde{\operatorname{E}}_{i}\bigg[g_{i, s_{1}}(\mathfrak{I}_{j})\big(1-\delta_{i, s_{1}\rightarrow s_{2}}(\mathfrak{I}_{j})/2\big)\exp\Big\{\boldsymbol{\beta}_{s_{1}\rightarrow s_{2}}^{\mathsf{T}}\boldsymbol{\eta}_ {i, s_{1}\rightarrow s_{2}}(\mathfrak{I}_{j})\Big\}\bigg].\end{align*}

We also note that a partial likelihood technique is available to simplify the expression for $ \frac{\partial\tilde{\operatorname{E}}[l_{c}^{(1)}]}{\partial\boldsymbol{\beta}_{s_{1}\rightarrow s_{2}}} $ and $ \frac{\partial^{2}\tilde{\operatorname{E}}[l_{c}^{(1)}]}{(\partial\boldsymbol{\beta}_{s_{1}\rightarrow s_{2}})^{2}} $, as detailed in Section S2.

The conditional expectations used in the above updating procedure can be simplified as follows using the methods in Gu et al. (2024):


\begin{align*}\tilde{\operatorname{E}}_{i}[\psi(\mathbf{b}_{i})] &=\frac{\operatorname{E}_{\mathbf{b}_{i}}[\psi(\mathbf{b}_{i})f(\mathcal{S}_{i}|\mathbf{b}_{i})|\mathcal{Y}_{i}]}{\operatorname{E}_{\mathbf{b} _{i}}[f(\mathcal{S}_{i}|\mathbf{b}_{i})|\mathcal{Y}_{i}]}\\ \tilde{\operatorname{E}}_{i}[\phi(\mathcal{A}_{i})\psi(\mathbf{b} _{i})] &=\frac{\operatorname{E}_{\mathbf{b}_{i}}\big[\operatorname{E}_{\mathcal{A}_{i}}[\phi(\mathcal{A}_{i})|\mathcal{S}_{i},\mathbf{b}_{i}]\psi(\mathbf{b}_{i})f(\mathcal{S}_{i}|\mathbf{b}_{i})\big|\mathcal{Y}_{i}\big]}{\operatorname{E}_{\mathbf{b}_{i}}[f(\mathcal{S}_{i}|\mathbf{b}_{i})|\mathcal{Y}_{i}]}.\end{align*}

$ \operatorname{E}_{\mathbf{b}_{i}}[\cdot|\mathcal{Y}_{i}] $ can be well-approximated by Gaussian quadrature or Monte-Carlo samples in $ \boldsymbol{\Omega}_{\mathbf{b}_{i}} $ as $ p(\mathbf{b}_{i}|\mathcal{Y}_{i}) $ is the probability density function for a multivariate normal variable. $ \operatorname{E}_{\mathcal{A}_{i}}[\phi(\mathcal{A}_{i})|\mathcal{S}_{i},\mathbf{b}_{i}] $ can take the following 2 forms and be evaluated correspondingly as


(4) \begin{align*}\operatorname{E}_{\mathcal{A}_{i}}[g_{i, s_{1}}(\mathfrak{I}_{j})\delta_{i, s_{1}\rightarrow s_{2}}(\mathfrak{I}_{j})|\mathcal{S}_{i},\mathbf{b} _{i}] &=\mathrm{P}_{\mathcal{A}_{i}}\big(g_{i, s_{1}}(\mathfrak{I}_{j})\delta_{i, s_{1}\rightarrow s_{2}}(\mathfrak{I}_{j})=1\big|\mathcal{S}_ {i},\mathbf{b}_{i}\big)=\kappa^{g\delta}_{i, j, s_{1},s_{2}}(\mathbf{b}_{i})/\kappa_{i}^{0}(\mathbf{b}_{i})\end{align*}

(5) \begin{align*}\operatorname{E}_{\mathcal{A}_{i}}[g_{i, s_{1}}(\mathfrak{I}_{j})|\mathcal{S}_{i},\mathbf{b}_{i}] &=\mathrm{P}_{\mathcal{A}_{i}}\big(g_{i, s_{1}}(\mathfrak{I}_{j})=1\big|\mathcal{S}_{i},\mathbf{b}_{i}\big)=\kappa^{g}_{i, j, s_{1}}(\mathbf{b}_{i})/\kappa_{i}^{0}(\mathbf{b}_{i}),\end{align*}

where for each * j * such that $ \mathfrak{I}_{j}\subset(T_{i, J-1},T_{iJ}] $,


\begin{align*} &\kappa^{g\delta}_{i, j, s_{1},s_{2}}(\mathbf{b}_{i})=[\![\boldsymbol{1}]\!]_{S_ {i0}}^{\mathsf{T}}\bigg[\prod\limits_{j=1}^{J-1}[\![\mathbf{P}_{i,(T_{i, j-1},T_{ij}]} (\mathbf{b}_{i};\boldsymbol{\Theta}_{\beta, h})]\!]_{S_{i, j-1},S_{ij}}\bigg][\![\mathbf{P}_{i,(T_{i, J-1},\tau_{j-1}]}(\mathbf{b}_{i};\boldsymbol{\Theta}_{\beta, h})]\!]_{S_{i, J-1},s_{1}}\\&\quad\times[\![\mathbf{P}_{i,(\tau_{j-1},\tau_{j}]}(\mathbf{b}_{i};\boldsymbol{\Theta}_{\beta, h})]\!]_{s_{1},s_{2}}[\![\mathbf{P}_{i,(\tau_{j},T_{iJ}]}(\mathbf{b}_{i};\boldsymbol{\Theta}_{\beta, h})]\!]_{s_{2},S_{i, J}}\\&\quad\times\bigg[\prod\limits_{j=J+1}^{n_{i}}[\![\mathbf{P}_{i,(T_{i, j-1},T_{ij}]}(\mathbf{b}_{i};\boldsymbol{\Theta}_{\beta, h})]\!]_{S_{i, j-1},S_{ij}}\bigg][\![\boldsymbol{1}]\!]_{S_{in_{i}}}\\&\kappa^{g}_{i, j, s_{1}}(\mathbf{b}_{i})=[\![\boldsymbol{1}]\!]_{S_{i0}}^{\mathsf{T}}\bigg[\prod\limits_{j=1}^{J-1}[\![\mathbf{P}_{i,(T_{i, j-1},T_{ij}]}(\mathbf{b}_{i};\boldsymbol{\Theta}_{\beta, h})]\!]_{S_{i, j-1},S_{ij}}\bigg][\![\mathbf{P} _{i,(T_{i, J-1},\tau_{j-1}]}(\mathbf{b}_{i};\boldsymbol{\Theta}_{\beta, h})]\!]_{S_{i, J-1} ,s_{1}}\\&\quad\times[\![\mathbf{P}_{i,(\tau_{j-1},T_{iJ}]}(\mathbf{b}_{i};\boldsymbol{\Theta}_{\beta, h})]\!]_{s_{1},S_{iJ}}\bigg[\prod\limits_{j=J+1}^{n_{i}}[\![\mathbf{P}_{i,(T_{i, j-1},T_{ij}]}(\mathbf{b}_{i};\boldsymbol{\Theta}_{\beta, h})]\!]_{S_{i, j-1},S _{ij}}\bigg][\![\boldsymbol{1}]\!]_{S_{in_{i}}}\\&\kappa^{0}_{i}(\mathbf{b}_{i};\boldsymbol{\Theta}_{\beta, h})=[\![\boldsymbol{1}]\!]_{S_{i0}}^{\mathsf{T}}\bigg[\prod\limits_{j=1}^{n_{i}}[\![\mathbf{P}_{i,(T_{i, j-1},T_{ij}]}(\mathbf{b}_{i};\boldsymbol{\Theta}_{\beta, h})]\!]_{S_{i, j-1},S_{ij}}\bigg][\![\boldsymbol{1}]\!]_{S_{in_{i}}},\end{align*}

and $ \operatorname{E}_{\mathcal{A}_{i}}[g_{i, s_{1}}(\mathfrak{I}_{j})\delta_{i, s_{1}\rightarrow s_{2}}(\mathfrak{I}_{j})|\mathcal{S}_{i},\mathbf{b}_{i}]=\operatorname{E}_{\mathcal{A}_{i}}[g_{i, s_{1}}(\mathfrak{I}_{j})|\mathcal{S}_{i},\mathbf{b}_{i}]=0 $ for each * j * such that $ \mathfrak{I}_{j}\subset(T_{in_{i}},\mathcal{T}] $, indicating censoring of all state transitions after time $ T_{in_{i}} $. The complete details on model estimation and techniques for simplifying the estimation procedure can be found in Sections S2 and S5. In particular, we would like to point out that in the numerical evaluation of $ \operatorname{E}_{\mathbf{b}_{i}}[\cdot|\mathcal{Y}_{i}] $ using Gaussian quadrature or Monte-Carlo samples, we have noticed that under extreme draws of $ \mathbf{b}_{i} $ from the density $ p(\mathbf{b}_{i}|\mathcal{Y}_{i}) $, the resulting transition probabilities may violate the following constraint, potentially leading to ill-posed transition matrices and numerical singularity:


\begin{align*} 1-\sum\limits_{s_{2}:s_{2}\neq s_{1}}\operatorname{P}\Big(\delta_{i, s_{1}\rightarrow s_{2}}(\mathfrak{I}_{j})=1\Big|g_{i, s_{1}}(\mathfrak{I}_{j})=1\Big) > 0.\end{align*}

In Section S2.2, we describe 3 complementary strategies for a systematic treatment of the problem. The first strategy excludes Monte Carlo draws that lead to nonpositive likelihood contributions, whereas the second and third strategies refine the discretization of the time intervals. These strategies are applied to ensure that the transition probability constraint is respected throughout estimation while avoiding ad hoc modifications to the likelihood after marginalization. As we recognize the importance of appropriately selecting the number of intervals $ N_{t} $, in Section S4, we also provide a discussion on how to choose $ N_{t} $ and present simulation studies evaluating its impact on the estimation procedure.

To provide an illustrative interpretation of our proposed estimation framework, some of the conditional expectations involved in the EM algorithm have natural probabilistic interpretations. Note that $ g_{i, s_{1}}(\mathfrak{I}_{j}) $ and $ \delta_{i, s_{1}\rightarrow s_{2}}(\mathfrak{I}_{j}) $ take values of 0 and 1 representing the unobserved true state occupation history. As a result, $ \tilde{\operatorname{E}}[g_{i, s_{1}}(\mathfrak{I}_{j})] $ can be interpreted as the probability of occupying state $ s_{1} $ immediately before entering interval $ \mathfrak{I}_{j} $, and $ \tilde{\operatorname{E}}[\delta_{i, s_{1}\rightarrow s_{2}}(\mathfrak{I}_{j})] $ can be interpreted as the probability of making the transition from state $ s_{1} $ to $ s_{2} $ during interval $ \mathfrak{I}_{j} $. We refer interested readers to the illustrative examples presented in Section S6, which may help provide a more intuitive understanding of the proposed method.

### 2.3. Dynamic predictions

#### 2.3.1. Computation of dynamic predictions

Suppose that a group of $ N^{\star} $ new individuals in the test dataset are being monitored for the risk of state transitions. Without introducing extra notations, we will index the individuals in the test data by $ i\,=\,N\,+\,1 , \ldots, N\,+\,N^{\star} $. Assume for now that the true state occupation process $ \{s_{i}(\mathfrak{I}_{j})\}_{j\,=\,1}^{N_{t}} $ is observed in the test dataset for evaluation. Predictions will be made based on the history of longitudinal data $ \mathcal{Y}_{i} $ and state occupation data $ \mathcal{S}_{i} $ up to the landmark time $ t_{\mathrm{L}} $ (ie $ T_{ij}\leq t_{\mathrm{L}} $ for $ j\,=\,1 , \ldots, n_{i} $; $ t_{ikj}\leq t_{\mathrm{L}} $ for $ k\,=\,1 , \ldots, N_{y} $ and $ j\,=\,1 , \ldots, n_{ik} $). We would like to predict the state occupation at time $ t_{\mathrm{LP}}=t_{\mathrm{L}}+t_{\mathrm{P}}\in(0 , \mathcal{T}] $. When $ t_{\mathrm{P}}\geq 0 $, $ t_{\mathrm{P}} $ is usually called prediction horizon (c.f., Blanche et al. 2015). In this manuscript, we will allow $ t_{\mathrm{P}} $ to be negative, which is useful when the event history is not fully observed and needs to be inferred. Due to the discrete approximations, we will translate $ t_{\mathrm{L}} $ and $ t_{\mathrm{P}} $ to intervals $ \mathfrak{I}_{\mathrm{L}} $ and $ \mathfrak{I}_{\mathrm{LP}} $ such that $ t_{\mathrm{L}}\in(\tau_{\mathrm{L}-1},\tau_{\mathrm{L}}]=\mathfrak{I}_{\mathrm{L}} $ and $ t_{\mathrm{LP}}\in(\tau_{\mathrm{LP}-1},\tau_{\mathrm{LP}}]=\mathfrak{I}_{\mathrm{LP}} $ for simplicity. Then, the probability of occupying state $ s_{1} $ at the end of $ \mathfrak{I}_{\mathrm{LP}} $ is


\begin{align*} p_{i, s_{1}}(t_{\mathrm{L}},t_{\mathrm{LP}})=\operatorname{P}(g_{i, s_ {1}}(\mathfrak{I}_{\mathrm{LP}+1})=1|\mathcal{S}_{i},\mathcal{Y}_{i})=\frac{\operatorname{E}_{\mathbf{b}_{i}}\big[\operatorname{P}_{\mathcal{A}_{i}}(g_{i, s_{1}}(\mathfrak{I}_{\mathrm{LP}+1})=1|\mathcal{S}_{i},\mathbf{b}_{i})f(\mathcal{S}_{i}|\mathbf{b}_{i})\big|\mathcal{Y}_{i}\big]}{\operatorname{E} _{\mathbf{b}_{i}}[f(\mathcal{S}_{i}|\mathbf{b}_{i})|\mathcal{Y}_{i}]},\end{align*}

where the formulas for $ \mathfrak{I}_{\mathrm{LP}}=\mathfrak{I}_{j}\subset(0, T_{in_{i}}] $ are given in (4) and (5) by replacing $ \mathfrak{I}_{j} $ with $ \mathfrak{I}_{\mathrm{LP}} $, and the formulas for $ \mathfrak{I}_{\mathrm{LP}}=\mathfrak{I}_{j}\subset(T_{in_{i}},\mathcal{T}] $ can be obtained by modifying $ \kappa^{g}_{i, j, s_{1}}(\mathbf{b}_{i}) $ to be


\begin{align*}\kappa^{g}_{i, j, s_{1}}(\mathbf{b}_{i}) =[\![\boldsymbol{1}]\!]_{S_{i0}}^{\mathsf{T}}\bigg[\prod\limits_{j=1}^{n_{i}}[\![\mathbf{P}_{i,(T_{i, j-1},T_{ij}]}(\mathbf{b}_{i};\boldsymbol{\Theta}_{\beta, h})]\!]_{S_{i, j-1},S_{ij}}\bigg][\![\mathbf{P}_{i,(T_{in_{i}},\tau_{\mathrm{LP}}]}(\mathbf{b}_{i};\boldsymbol{\Theta}_{\beta, h})]\!]_{S_{in_{i}},s_{1}}.\end{align*}

From our preliminary numerical experiments, we identified several cases where the numerical singularities may arise in the dynamic predictions and will discuss strategies for avoiding singularities below. The first case involves transitions in the test dataset that do not appear in the training dataset. For a simple example, consider an individual * i * in the test dataset with a transition from $ s_{1} $ to $ s_{2} $ ($ S_{i, j-1}=\{s_{1}\} $ and $ S_{ij}=\{s_{2}\} $). If no transitions from $ s_{1} $ to $ s_{2} $ are observed in the training dataset, the quantity $ [\![\mathbf{P}_{i,(T_{i, j-1},T_{ij}]}(\mathbf{b}_{i})]\!]_{S_{i, j-1},S_{ij}} $ will be 0 and singularities arise as the denominator of $ \operatorname{E}_{\mathcal{A}_{i}}[g_{i, s_{1}}(\mathfrak{I}_{j})|\mathcal{S}_{i},\mathbf{b}_{i}]=\kappa^{g}_{i, j, s_{1}}(\mathbf{b}_{i})/\kappa_{i}^{0}(\mathbf{b}_{i}) $ is 0. To prevent such singularities, we first recommend smoothing the estimated hazard rate using the kernel smoothing by modifying the method in Gray (1990)


\begin{align*}\tilde{h}_{0, s_{1}\rightarrow s_{2}}(\mathfrak{I}_{j})=\frac{\sum_{k=1}^{N_{t}}\hat{h}_{0, s_{1}\rightarrow s_{2}}(\mathfrak{I}_{k})K\big((\tilde{\tau}_{j} -\tilde{\tau}_{k})\big{/}w\big)}{\sum_{k=1}^{N_{t}}K\big((\tilde{\tau}_{j} -\tilde{\tau}_{k})\big{/}w\big)},\end{align*}

where * w * is a bandwidth parameter that controls the widths of data we would like to borrow for estimation, and the kernel function * K(t) * is a bell-shaped probability density function, smooth over its support and symmetric around 0. In this manuscript, we let $ K(t)=0.75(1-t^{2})\times I(t\in[-1,1]) $ known as the Epanechnikov kernel (c.f., Epanechnikov 1969). We will replace $ \hat{h}_{0, s_{1}\rightarrow s_{2}}(\mathfrak{I}_{j}) $ by $ \tilde{h}_{0, s_{1}\rightarrow s_{2}}(\mathfrak{I}_{j}) $ in the dynamic prediction algorithm. This modification reduces the chances of singularities, as $ \tilde{h}_{0, s_{1}\rightarrow s_{2}}(\mathfrak{I}_{j}) $ will be nonzero whenever an event is observed within the range of * w * in the training dataset. In addition to the smoothing method, we implemented a further measure by replacing the zero elements of $ [\![\mathbf{P}_{i,(T_{i, j-1},T_{ij}]}(\mathbf{b}_{i})]\!]_{S_{i, j-1},S_{ij}} $ with a negligibly small number ($ 10^{-5} $ in our case) for $ (s_{1},s_{2})\in\mathbb{E} $. This technique is analogous to the padding method commonly used in the machine learning literature, which is designed to minimally affect cases without singularities while effectively addressing cases where singularities may occur.

Another case of numerical singularity is related to the calculation of transition probability matrices in (2). When the sum $ \sum_{s_{2}:s_{2}\neq s_{1}}\operatorname{P}\big(\delta_{i, s_{1}\rightarrow s _{2}}(\mathfrak{I}_{j})=1\big|g_{i, s_{1}}(\mathfrak{I}_{j})=1\big) $ is larger than 1, the formula results in a negative value for $ \operatorname{P}_{i, \mathfrak{I}_{j}}(\mathbf{b}_{i}) $ in the $ (s_{1},s_{1}) $ entry. This typically happens when there are extreme values in the longitudinal or non-time-varying covariates $ \boldsymbol{\eta}_{i}(\mathfrak{I}_{j}) $ that fall outside the range of the distribution observed in the training data, which leads to large values of $\operatorname{P}\big(\delta_{i, s_{1}\rightarrow s_{2}}(\mathfrak{I}_{j})=1\big|g_{i, s_{1}}(\mathfrak{I}_{j})=1\big)=1-\exp\big\{-h_{0, s_{1}\rightarrow s_{2}}(\mathfrak{I}_{j})\exp\big[\boldsymbol{\beta}_{s_{1}\rightarrow s_ {2}}^{\mathsf{T}}\boldsymbol{\eta}_{i, s_{1}\rightarrow s_{2}}(\mathfrak{I}_{j})\big]\big\} $. Besides checking the test data for potential outliers, we can regularize the transition probabilities by replacing Formula (2) with


\begin{align*}\mathbf{P}_{i , \mathfrak{I}_{j}}(\mathbf{b}_{i})=\begin{cases}\mathrm{P}\Big(\delta_{i, s_{1}\rightarrow s_{2}}(\mathfrak{I}_{j})=1\Big|g_{i, s_{1}} (\mathfrak{I}_{j})=1\Big)\Big{/}F&s_{1}\neq s_{2}\\1-\sum\nolimits_{s_{2}:s_{2}\neq s_{1}}\operatorname{P}\Big(\delta_{i, s_{1}\rightarrow s_{2}}(\mathfrak{I}_{j})=1\Big|g_{i, s_{1}}(\mathfrak{I}_{j})=1\Big)\Big{/}F&s_{1}=s_{2}\end{cases}\end{align*}

where $ F=\max\big\{1 , \sum_{s_{2}:s_{2}\neq s_{1}}\operatorname{P}\big(\delta_{i, s _{1}\rightarrow s_{2}}(\mathfrak{I}_{j})=1\big|g_{i, s_{1}}(\mathfrak{I}_{j}) =1\big)\big\} $ is a regularization factor. This modification guarantees that all elements of $ \mathbf{P}_{i , \mathfrak{I}_{j}}(\mathbf{b}_{i}) $ are nonnegative.

#### 2.3.2. Evaluation of dynamic predictions

Evaluation of predictions based on time-to-event models in the presence of interval-censored data has been discussed in the literature, primarily using the dynamic receiver operating characteristics (ROC) curves (Díaz-Coto et al. 2020; Beyene and El Ghouch 2022; Wu and Cook 2022). Alternative metrics proposed in the literature include Brier scores (BS) (Blanche et al. 2015), prediction errors (Wu and Cook 2022), and process monitoring characteristics (Qiu et al. 2020). Next, we will discuss several such metrics for evaluating the prediction accuracy in the current context. When the primary interest is to predict the occupation of the terminal state $ s_{e} $, Blanche et al. (2015) proposed to use dynamic ROC curves and dynamic BS. The dynamic ROC curves for a landmark time $ t_{\mathrm{L}} $ and a prediction horizon $ t_{\mathrm{P}} $ is the graph of the map from the threshold * ρ * to the false positive rate (FPR) and true positive rate (TPR): $ \rho\mapsto(\mathrm{FPR}(\rho, t_{\mathrm{L}},t_{\mathrm{LP}}),\mathrm{TPR}(\rho, t_{\mathrm{L}},t_{\mathrm{LP}})) $, where $ \rho\in[0,1] $, and


\begin{align*}\mathrm{TPR}_{s_{e}}(\rho, t_{\mathrm{L}},t_{\mathrm{LP}}) &=\operatorname{P}\big(\hat{p}_{i, s_{e}}(t_{\mathrm{L}},t_{\mathrm{LP}}) \gt\rho\big|s_{i}(t_{\mathrm{L}})\neq s_{e},s_{i}(t_{\mathrm{LP}})=s_{e}\big)\\\mathrm{FPR}_{s_{e}}(\rho, t_{\mathrm{L}},t_{\mathrm{LP}}) &=\operatorname{P}\big(\hat{p}_{i, s_{e}}(t_{\mathrm{L}},t_{\mathrm{LP}}) \gt\rho\big|s_{i}(t_{\mathrm{L}})\neq s_{e},s_{i}(t_{\mathrm{LP}})\neq s_{e}\big),\end{align*}

where $ \hat{p}_{i, s}(t_{\mathrm{L}},t_{\mathrm{LP}}) $ denotes the predicted probability of occupying state * s * at $ t_{\mathrm{LP}} $ based on a landmark time $ t_{\mathrm{L}} $. It is generally desirable to have a higher TPR and a lower FPR across a range of thresholds. The dynamic area under the ROC curve (AUC) is a prediction metric to summarize the performance by balancing TPR and FPR, and it is defined by


\begin{align*} &\mathrm{AUC}_{s_{e}}(t_{\mathrm{L}},t_{\mathrm{LP}})=\int_{0}^{1}\mathrm{TPR}(\rho)\, d\mathrm{FPR}(\rho)\\&\quad=\operatorname{P}\big(\hat{p}_{i, s_{e}}(t_{\mathrm{L}},t_{\mathrm{LP}}) \gt\hat{p}_{l, s_{e}}(t_{\mathrm{L}},t_{\mathrm{LP}})\big|s_{i}(t_{\text {L}})\neq s_{e},s_{l}(t_{\mathrm{L}})\neq s_{e},s_{i}(t_{\mathrm{LP}})=s_{e},s_{l} (t_{\mathrm{LP}})\neq s_{e}\big),\end{align*}

where * l * denotes an individual independent of * i * ($ l\,=\,N\,+\,1 , \ldots, N\,+\,N^{\star} $, * l≠q i *). The dynamic BS is defined by


\begin{align*}\mathrm{BS}_{s_{e}}(t_{\mathrm{L}},t_{\mathrm{LP}})=\operatorname{E}\Big[\big(I(s_{i}(t_{\mathrm{LP}})=s_{e})-\hat{p}_{i, s_{e}}(t_{\mathrm{L}},t_{\mathrm{LP}})\big{)}^{2}\Big|s_{i}(t_{\mathrm{L}})\neq s_{e}\Big].\end{align*}

The above BS evaluates the accuracy of predicting the end state $ s_{e} $, with a lower BS indicating a higher alignment between the predicted probabilities of occupying state $ s_{e} $ and the actual status. In cases where the interest is to evaluate the prediction of all states, we can adopt the following adaption to the BS:


\begin{align*}\mathrm{BS}_{\mathbb{V}}(t_{\mathrm{L}},t_{\mathrm{LP}})=\operatorname{E}\bigg[\sum\limits_{s\in\mathbb{V}}\big(I(s_{i}(t_{\mathrm{LP}})=s)-\hat{p}_{i, s}(t_{\mathrm{L}},t_{\mathrm{LP}})\big)^{2}\bigg|s_{i}(t_{\mathrm{L}})\neq s_{e}\bigg],\end{align*}

where a lower BS indicates a higher alignment between the predicted state occupation probabilities and the actual state occupations for all states.

The above performance measures can be estimated using test data as follows:


\begin{align*}\hat{\mathrm{TPR}}_{s_{e}}(\rho, t_{\mathrm{L}},t_{\mathrm{LP}}) &=\frac{\sum_{i=N+1}^{N+N^{\star}}I\big(\hat{p}_{i, s_{e}}(t_{\mathrm{L}},t_{\mathrm{LP}}) \gt\rho\big)I\big(s_{i}(t_{\mathrm{L}})\neq s_{e},s_{i}(t_{\mathrm{LP}})=s_{e}\big)}{\sum_{i=N+1}^{N+N^{\star}}I\big(s_{i}(t_{\mathrm{L}})\neq s_{e},s_{i}(t_{\mathrm{LP}})=s_{e}\big)}\\\hat{\mathrm{FPR}}_{s_{e}}(\rho, t_{\mathrm{L}},t_{\mathrm{LP}}) &=\frac{\sum_{i=N+1}^{N+N^{\star}}I\big(\hat{p}_{i, s_{e}}(t_{\mathrm{L}},t_{\mathrm{LP}}) \gt\rho\big)I\big(s_{i}(t_{\mathrm{L}})\neq s_{e},s_{i}(t_{\mathrm{LP}})\neq s_{e}\big)}{\sum_{i=N+1}^{N+N^{\star}}I\big(s_{i}(t_{\mathrm{L}})\neq s_{e},s_{i}(t_{\mathrm{LP}})\neq s_{e}\big)}\\\hat{\mathrm{AUC}}_{s_{e}}(t_{\mathrm{L}},t_{\mathrm{LP}}) &=\int_{0}^{1}\hat{\mathrm{TPR}}(\rho)\, d\hat{\mathrm{FPR}}(\rho)\\\hat{\mathrm{BS}}_{s_{e}}(t_{\mathrm{L}},t_{\mathrm{LP}}) &=\frac{\sum_{i=N+1}^{N+N^{\star}}\big(I(s_{i}(t_{\mathrm{LP}})=s_ {e})-\hat{p}_{i, s_{e}}(t_{\mathrm{L}},t_{\mathrm{LP}})\big)^{2}I\big(s_{i}(t_{\mathrm{L}})\neq s_{e}\big)}{\sum_{i=N+1}^{N+N^{\star}}I\big(s_{i}(t_{\mathrm{L}})\neq s_{e}\big)}\\\hat{\mathrm{BS}}_{\mathbb{V}}(t_{\mathrm{L}},t_{\mathrm{LP}}) &=\frac{\sum_{i=N+1}^{N+N^{\star}}\sum_{s\in\mathbb{V}}\big(I(s_ {i}(t_{\mathrm{LP}})=s)-\hat{p}_{i, s}(t_{\mathrm{L}},t_{\mathrm{LP}})\big)^{2}I\big(s_{i}(t_{\mathrm{L}})\neq s_{e}\big)}{\sum_{i=N+1}^{N+N^{\star}}I\big{(}s_{i}(t_{\mathrm{L}})\neq s_{e}\big)}.\end{align*}

In practice, there could be cases where the true state occupation processes $ \{s_{i}(\mathfrak{I}_{j})\}_{j\,=\,1}^{N_{t}} $ are not completely observed as in the training dataset. Namely, in the test dataset, we observe the state occupation information $ S_{i1},\ldots, S_{in_{i}^{*}} $ at times $ T_{i0},\ldots, T_{in_{i}^{*}} $ and the * k *th longitudinal variable $ y_{ik1},\ldots, y_{ikn_{ik}^{*}} $ at times $ t_{ik1},\ldots, t_{ikn_{ik}^{*}} $, where the numbers of observations $ n_{i}^{*} $ and $ n_{ik}^{*} $ indicate that the observational time sequences are extended beyond the landmark time $ t_{\mathrm{L}} $ to include all available information in the test dataset and the collections of all longitudinal and state occupation data are denoted by $ \mathcal{S}_{i}^{*} $ and $ \mathcal{Y}_{i}^{*} $. In such cases, we adopt the method by Beyene and El Ghouch (2022) to adjust for interval censoring. Then, TPR, FPR, and AUC can be estimated by


\begin{align*}\tilde{\mathrm{TPR}}_{s_{e}}(\rho, t_{\mathrm{L}},t_{\mathrm{LP}}) &=\frac{\sum_{i=N+1}^{N+N^{\star}}I\big(\hat{p}_{i, s_{e}}(t_{\mathrm{L}},t_{\mathrm{LP}}) \gt\rho\big)\hat{\operatorname{P}}\big(s_{i}(t_{\mathrm{L}})\neq s_{e},s_{i}(t_{\mathrm{LP}})=s_{e}\big|\mathcal{S}_{i}^{*},\mathcal{Y}_{i}^{*}\big)}{\sum_{i=N+1}^{N+N^{\star}}\hat{\operatorname{P}}\big(s_{i}(t_{\mathrm{L}})\neq s_{e},s_{i}(t_{\mathrm{LP}})=s_{e}\big|\mathcal {S}_{i}^{*},\mathcal{Y}_{i}^{*}\big)}\\\tilde{\mathrm{FPR}}_{s_{e}}(\rho, t_{\mathrm{L}},t_{\mathrm{LP}}) &=\frac{\sum_{i=N+1}^{N+N^{\star}}I\big(\hat{p}_{i, s_{e}}(t_{\mathrm{L}},t_{\mathrm{LP}}) \gt\rho\big)\hat{\operatorname{P}}\big(s_{i}(t_{\mathrm{L}})\neq s_{e},s_{i}(t_{\mathrm{LP}})\neq s_{e}\big|\mathcal{S}_{i}^{*},\mathcal{Y}_{i}^{*}\big)}{\sum_{i=N+1}^{N+N^{\star}}\hat{\operatorname{P}}\big(s_{i}(t_{\mathrm{L}})\neq s_{e},s_{i}(t_{\mathrm{LP}})\neq s_{e}\big|\mathcal{S}_{i}^{*},\mathcal{Y}_{i}^{*}\big)}\\\tilde{\mathrm{AUC}}_{s_{e}}(t_{\mathrm{L}},t_{\mathrm{LP}}) &=\int_{0}^{1}\tilde{\mathrm{TPR}}(\rho)\, d\tilde{\mathrm{FPR}}(\rho),\end{align*}

where we use $ \hat{P} $ to denote the estimated conditional probabilities by the estimated parameters. We can see that the true positives are weighed by $ \hat{\operatorname{P}}\big(s_{i}(t_{\mathrm{L}})\neq s_{e},s_{i}(t_{\mathrm{LP}})=s_{e}\big|\mathcal{S}_{i}^{*},\mathcal{Y}_{i}^{*}\big) $ and the false positives are weighed by $ \hat{\operatorname{P}}\big(s_{i}(t_{\mathrm{L}})\neq s_{e},s_{i}(t_{\mathrm{LP}})\neq s_{e}\big|\mathcal{S}_{i}^{*},\mathcal{Y}_{i}^{*}\big) $, representing the probabilities of observing these cases. Similarly, BS can be estimated by


\begin{align*} &\tilde{\mathrm{BS}}_{s_{e}}(t_{\mathrm{L}},t_{\mathrm{LP}})=\sum\limits_{i=N+1}^{N+N^{\star}}\Big[\big(1-\hat{p}_{i, s_{e}}(t_{\mathrm{L}},t_{\mathrm{LP}})\big)^{2}\hat{\operatorname{P}}\big(s_{i}(t_{\mathrm{L}})\neq s_{e},s_{i}(t_ {\mathrm{LP}})=s_{e}\big|\mathcal{S}_{i}^{*},\mathcal{Y}_{i}^{*}\big)\\&\quad+\hat{p}_{i, s_{e}}(t_{\mathrm{L}},t_{\mathrm{LP}})^{2}\hat{\operatorname{P}}\big(s_{i}(t_{\mathrm{L}})\neq s_{e},s_{i}(t_{\mathrm{LP}})\neq s _{e}\big|\mathcal{S}_{i}^{*},\mathcal{Y}_{i}^{*}\big)\Big]\bigg{/}\sum\limits_{i=N+1}^{N+N^{\star}}\hat{\operatorname{P}}\big(s_{i}(t_{\mathrm{L}})\neq s_{e}\big|\mathcal{S}_{i}^{*},\mathcal{Y}_{i}^{*}\big)\\&\tilde{\mathrm{BS}}_{\mathbb{V}}(t_{\mathrm{L}},t_{\mathrm{LP}})=\sum\limits_{i=N+1}^{N+N^{\star}}\sum\limits_{s\in\mathbb{V}}\Big[\big(1-\hat{p}_{i, s}(t_{\mathrm{L}},t_{\mathrm{LP}})\big)^{2}\hat{\operatorname{P}}\big(s_{i}(t_{\text {L}})\neq s_{e},s_{i}(t_{\mathrm{LP}})=s\big|\mathcal{S}_{i}^{*},\mathcal{Y}_{i}^{*}\big)\\&\quad+\hat{p}_{i, s}(t_{\mathrm{L}},t_{\mathrm{LP}})^{2}\hat{\operatorname{P}}\big(s_{i}(t_{\mathrm{L}})\neq s_{e},s_{i}(t_{\mathrm{LP}})\neq s\big|\mathcal{S}_{i}^{*},\mathcal{Y}_{i}^{*}\big)\Big]\bigg{/}\sum\limits_{i=N+ 1}^{N+N^{\star}}\hat{\operatorname{P}}\big(s_{i}(t_{\mathrm{L}})\neq s_{e}\big|\mathcal{S}_{i}^{*},\mathcal{Y}_{i}^{*}\big),\end{align*}

## 3. Simulation studies

### 3.1. Estimation

We conducted some simulation studies to evaluate the performance of the proposed method. In the simulation studies, the design interval $ (0 , \mathcal{T}]=(0,1] $ is partitioned in to the union of $ N_{t}=200 $ intervals $ \cup_{j\,=\,1}^{N_{t}}\mathfrak{I}_{j}=\cup_{j\,=\,1}^{N_{t}}((j-1)/200, j/200] $. The sample size in the training dataset is $ N\,=\,500 $. The main analyses were conducted on a Linux-based supercomputing system with a 64-bit x86 architecture using Julia (version 1.6.7), while tables and summaries were generated on a local Windows 11 x64 machine using R (version 4.3.1).

**Figure 2:**
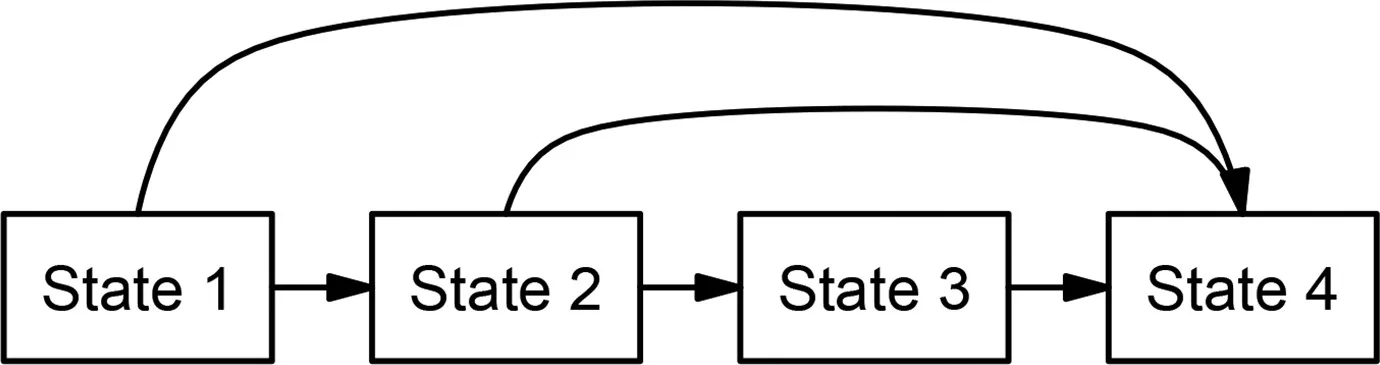
Multistate model for Simulation 1.

In Simulation 1, we considered a multistate model with $ N_{s}=4 $ states as illustrated in Fig. 2, where each nonterminal state can directly go to the terminal state (State 4). The model includes $ N_{y}=2 $ longitudinal and $ N_{z}=2 $ non-time-varying variables. The first dimension of longitudinal risk factor $ m_{i1}(t) $ follows a nonlinear trajectory simulated from B-splines of 6 degrees with equally spaced inner knots, while the second dimension $ m_{i2}(t) $ follows a linear trajectory. Parameters $ \mathbf{c}=(0.2,0.1,0,0,0,0,0.2,0.1)^{\mathsf{T}} $, $ \boldsymbol{\Sigma}_{a}=\operatorname{diag}\{1.0,0.5,0.4,0.3,0.2,0.1,1.0,0.5\} $, where $ \mathbf{B}_{k}(t) $ are Demmler-Reinsch orthogonalized B-spline and linear basis functions (c.f., Demmler and Reinsch 1975). The non-time-varying variables are simulated independently from the standard normal distribution. Transition times are simulated by discrete-time approximations detailed in Section S7 where the true values of the regression coefficients are given in Table 1. $ h_{0,1\rightarrow 2}(t)=\cos(t) $, $ h_{0,1\rightarrow 4}(t)=\sin(t) $, $ h_{0,2\rightarrow 3}(t)=\exp(-t/2) $, $ h_{0,2\rightarrow 4}(t)=\log(1+t) $, and $ h_{0,3\rightarrow 4}(t)=\sqrt{t} $. The differences between consecutive monitoring times $ T_{ij}-T_{i, j-1}=\Delta T_{ij}+0.04 $ where $ \Delta T_{ij} $ follow a Gamma distribution with the shape parameter and rate parameter being 2 and 0.01. When simulating censored states $ S_{ij} $, we first let $ S_{ij} $ include the true state occupied at $ T_{ij} $ (ie $ s_{i}(T_{ij})\in S_{ij} $), then we simulated the remaining components of $ S_{ij} $ by independently including each other state with a 5% probability. The simulation is repeated 500 times. The biases of parameter estimates are presented in Table 1. We compared the proposed method with the multistate model implemented in the R package msm (“MSM”). Both methods allow state occupation information to be censored. However, they use different representations of the longitudinal covariate process. In our proposed joint model, the transition process depends on the latent marker trajectory. In contrast, MSM does not incorporate a longitudinal submodel and the observed longitudinal risk factors were modeled as external piecewise-constant time-varying covariates. Therefore, the estimated covariate effects are not directly comparable.

**Table 1:** True values of regression coefficients $ \boldsymbol{\beta}_{s_{1}\rightarrow s_{2}} $ and the biases of estimated regression coefficients in Simulation 1, where $ \boldsymbol{\beta}_{s_{1}\rightarrow s_{2}} $ measures the strength of associations between risk factors and the transitions from $ s_{1} $ to $ s_{2} $.

Parameter	Truth	Proposed	MSM
$ [\![\boldsymbol{\beta}_{1\rightarrow 2}]\!]_{\{1\}} $	0.6	0.007 (0.003)	−0.166 (0.002)
$ [\![\boldsymbol{\beta}_{1\rightarrow 2}]\!]_{\{2\}} $	0.2	0.001 (0.003)	−0.106 (0.001)
$ [\![\boldsymbol{\beta}_{1\rightarrow 2}]\!]_{\{3\}} $	0.2	0.000 (0.003)	−0.104 (0.001)
$ [\![\boldsymbol{\beta}_{1\rightarrow 4}]\!]_{\{1\}} $	0.3	0.001 (0.005)	−0.143 (0.003)
$ [\![\boldsymbol{\beta}_{1\rightarrow 4}]\!]_{\{2\}} $	0.2	0.001 (0.005)	−0.139 (0.002)
$ [\![\boldsymbol{\beta}_{1\rightarrow 4}]\!]_{\{3\}} $	0.5	0.013 (0.005)	−0.312 (0.002)
$ [\![\boldsymbol{\beta}_{2\rightarrow 3}]\!]_{\{1\}} $	0.3	0.011 (0.004)	−0.078 (0.003)
$ [\![\boldsymbol{\beta}_{2\rightarrow 3}]\!]_{\{2\}} $	0.6	0.025 (0.005)	−0.241 (0.004)
$ [\![\boldsymbol{\beta}_{2\rightarrow 3}]\!]_{\{3\}} $	0.2	0.014 (0.004)	−0.104 (0.002)

Parameter	Truth	Proposed	MSM
$ [\![\boldsymbol{\beta}_{2\rightarrow 3}]\!]_{\{4\}} $	0.2	−0.001 (0.005)	−0.108 (0.002)
$ [\![\boldsymbol{\beta}_{2\rightarrow 4}]\!]_{\{1\}} $	0.3	0.010 (0.007)	−0.135 (0.005)
$ [\![\boldsymbol{\beta}_{2\rightarrow 4}]\!]_{\{2\}} $	0.3	−0.009 (0.007)	−0.134 (0.005)
$ [\![\boldsymbol{\beta}_{2\rightarrow 4}]\!]_{\{3\}} $	0.2	−0.005 (0.007)	−0.136 (0.003)
$ [\![\boldsymbol{\beta}_{2\rightarrow 4}]\!]_{\{4\}} $	0.5	0.030 (0.008)	−0.297 (0.003)
$ [\![\boldsymbol{\beta}_{3\rightarrow 4}]\!]_{\{1\}} $	0.3	0.037 (0.006)	−0.101 (0.004)
$ [\![\boldsymbol{\beta}_{3\rightarrow 4}]\!]_{\{2\}} $	0.3	0.024 (0.007)	−0.139 (0.004)
$ [\![\boldsymbol{\beta}_{3\rightarrow 4}]\!]_{\{3\}} $	0.5	0.036 (0.007)	−0.292 (0.003)
$ [\![\boldsymbol{\beta}_{3\rightarrow 4}]\!]_{\{4\}} $	0.5	0.045 (0.008)	−0.290 (0.003)

In Simulation 1, $ [\![\boldsymbol{\beta}_{s_{1}\rightarrow s_{2}}]\!]_{\{1\}} $ and $ [\![\boldsymbol{\beta}_{s_{1}\rightarrow s_{2}}]\!]_{\{2\}} $ correspond to the longitudinal risk factors, while $ [\![\boldsymbol{\beta}_{s_{1}\rightarrow s_{2}}]\!]_{\{3\}} $ and $ [\![\boldsymbol{\beta}_{s_{1}\rightarrow s_{2}}]\!]_{\{4\}} $ correspond to the non-time-varying risk factors.

**Figure 3:**
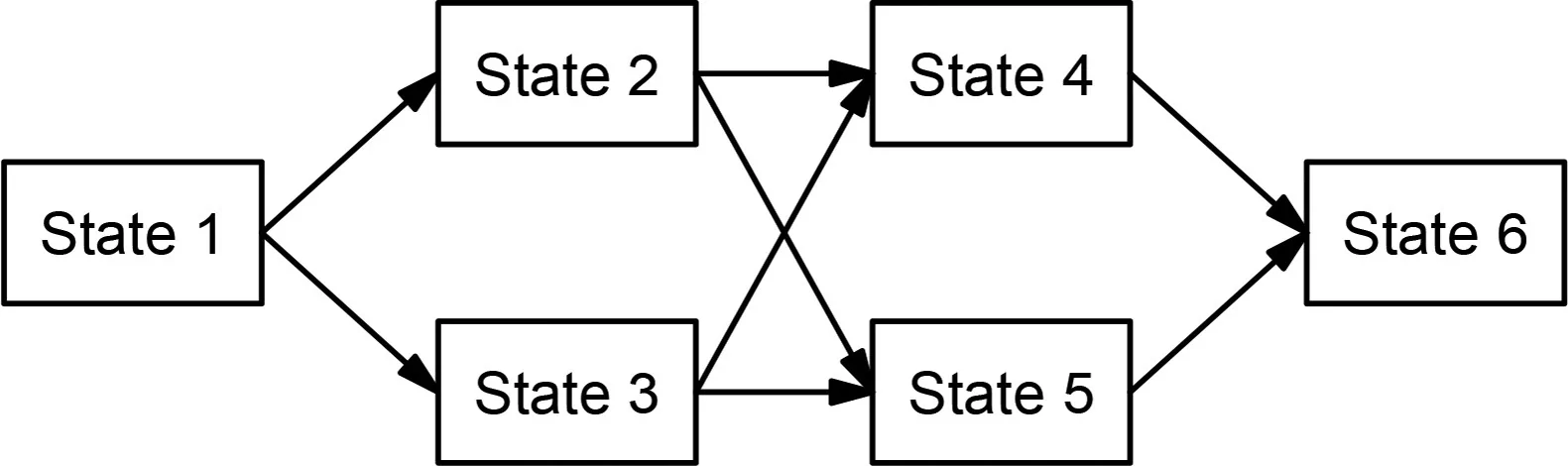
Multistate model for Simulation 2.

In Simulation 2, we considered a more complicated multistate model as illustrated in Fig. 3. The model includes $ N_{y}=2 $ longitudinal and $ N_{z}=2 $ non-time-varying variables. The first and second longitudinal dimensions are assumed to follow cubic and quadratic polynomial trajectories, respectively. We set $ \mathbf{c}=(0.3,0.2,0.1,0,0.3,0.2,0.1)^{\mathsf{T}} $, and and specify $ \boldsymbol{\Sigma}_{a} $ as a block diagonal matrix with diagonal matrix with a * 4× 4 * block $ \operatorname{diag}\{0.6,0.3,0.2,0.1\}+0.05\times\boldsymbol{1}_{4\times 4} $ for the first dimension of longitudinal variables, and $ \operatorname{diag}\{0.6,0.3,0.2\}+0.05\times\boldsymbol{1}_{3\times 3} $ for the second. The non-time-varying variables are simulated independently from the Bernoulli distribution with the probability parameter being 0.5. Transition times are simulated from the multistate model where the true values of the regression coefficients are given in Table 2, and $ h_{0, s_{1}\rightarrow s_{2}}(t)=0.6\,+\,0.1\sin(10t+s_{1}+s_{2}) $ for all $ (s_{1},s_{2})\in\mathbb{E} $. The differences between consecutive monitoring times $ T_{ij}-T_{i, j-1}=\Delta T_{ij}+0.04 $ where $ \Delta T_{ij} $ follows a Gamma distribution with shape and rate parameters 2 and 0.01. When simulating censored states, on top of the true state $ s_{i}(T_{ij}) $, $ S_{ij} $ includes other states with an independent 5% probability. The simulation is repeated 500 times. In Simulations 1 and 2, model convergence failed in 1 and 9 out of 500 repetitions, respectively. For these failed runs, refitting the model with an increased number of subintervals ($ N_{t}=400 $) resulted in successful convergence in all cases. The biases and root mean square errors (RMSE) of parameter estimates are presented in Table 2. The code for implementing the proposed method is available on GitHub at https://github.com/luyouepiusf/dynamic\_prediction\_uncertainties.

**Table 2:** True values of regression coefficients $ \boldsymbol{\beta}_{s_{1}\rightarrow s_{2}} $ and the biases of estimated regression coefficients in Simulation 2, where $ \boldsymbol{\beta}_{s_{1}\rightarrow s_{2}} $ measures the strength of associations between risk factors and the transitions from $ s_{1} $ to $ s_{2} $.

Parameter	Truth	Proposed	MSM
$ [\![\boldsymbol{\beta}_{1\rightarrow 2}]\!]_{\{1\}} $	0.6	0.012 (0.003)	−0.186 (0.002)
$ [\![\boldsymbol{\beta}_{1\rightarrow 2}]\!]_{\{2\}} $	0.4	0.008 (0.004)	−0.147 (0.003)
$ [\![\boldsymbol{\beta}_{1\rightarrow 2}]\!]_{\{3\}} $	0.5	0.002 (0.006)	−0.467 (0.000)
$ [\![\boldsymbol{\beta}_{1\rightarrow 2}]\!]_{\{4\}} $	0.3	−0.004 (0.007)	−0.281 (0.000)
$ [\![\boldsymbol{\beta}_{1\rightarrow 3}]\!]_{\{1\}} $	0.4	0.013 (0.003)	−0.132 (0.002)
$ [\![\boldsymbol{\beta}_{1\rightarrow 3}]\!]_{\{2\}} $	0.6	0.012 (0.003)	−0.227 (0.003)
$ [\![\boldsymbol{\beta}_{1\rightarrow 3}]\!]_{\{3\}} $	0.3	0.003 (0.006)	−0.281 (0.000)
$ [\![\boldsymbol{\beta}_{1\rightarrow 3}]\!]_{\{4\}} $	0.5	0.015 (0.007)	−0.467 (0.000)
$ [\![\boldsymbol{\beta}_{2\rightarrow 4}]\!]_{\{1\}} $	0.6	0.035 (0.006)	−0.180 (0.004)
$ [\![\boldsymbol{\beta}_{2\rightarrow 4}]\!]_{\{2\}} $	0.4	0.017 (0.006)	−0.139 (0.004)
$ [\![\boldsymbol{\beta}_{2\rightarrow 4}]\!]_{\{3\}} $	0.3	0.018 (0.012)	−0.282 (0.001)
$ [\![\boldsymbol{\beta}_{2\rightarrow 4}]\!]_{\{4\}} $	0.3	0.009 (0.012)	−0.281 (0.001)
$ [\![\boldsymbol{\beta}_{2\rightarrow 5}]\!]_{\{1\}} $	0.4	0.033 (0.006)	−0.113 (0.004)
$ [\![\boldsymbol{\beta}_{2\rightarrow 5}]\!]_{\{2\}} $	0.4	0.012 (0.006)	−0.145 (0.004)
$ [\![\boldsymbol{\beta}_{2\rightarrow 5}]\!]_{\{3\}} $	0.5	0.037 (0.012)	−0.466 (0.001)
$ [\![\boldsymbol{\beta}_{2\rightarrow 5}]\!]_{\{4\}} $	0.3	0.020 (0.012)	−0.280 (0.001)

Parameter	Truth	Proposed	MSM
$ [\![\boldsymbol{\beta}_{3\rightarrow 4}]\!]_{\{1\}} $	0.4	0.025 (0.006)	−0.113 (0.004)
$ [\![\boldsymbol{\beta}_{3\rightarrow 4}]\!]_{\{2\}} $	0.4	0.016 (0.007)	−0.141 (0.005)
$ [\![\boldsymbol{\beta}_{3\rightarrow 4}]\!]_{\{3\}} $	0.3	0.001 (0.012)	−0.281 (0.001)
$ [\![\boldsymbol{\beta}_{3\rightarrow 4}]\!]_{\{4\}} $	0.5	0.045 (0.012)	−0.466 (0.001)
$ [\![\boldsymbol{\beta}_{3\rightarrow 5}]\!]_{\{1\}} $	0.4	0.026 (0.006)	−0.118 (0.004)
$ [\![\boldsymbol{\beta}_{3\rightarrow 5}]\!]_{\{2\}} $	0.6	0.034 (0.007)	−0.222 (0.005)
$ [\![\boldsymbol{\beta}_{3\rightarrow 5}]\!]_{\{3\}} $	0.3	0.008 (0.012)	−0.282 (0.001)
$ [\![\boldsymbol{\beta}_{3\rightarrow 5}]\!]_{\{4\}} $	0.3	−0.000 (0.012)	−0.283 (0.001)
$ [\![\boldsymbol{\beta}_{4\rightarrow 6}]\!]_{\{1\}} $	0.6	0.026 (0.005)	−0.181 (0.003)
$ [\![\boldsymbol{\beta}_{4\rightarrow 6}]\!]_{\{2\}} $	0.4	0.018 (0.005)	−0.128 (0.003)
$ [\![\boldsymbol{\beta}_{4\rightarrow 6}]\!]_{\{3\}} $	0.3	0.014 (0.010)	−0.282 (0.001)
$ [\![\boldsymbol{\beta}_{4\rightarrow 6}]\!]_{\{4\}} $	0.5	0.015 (0.011)	−0.469 (0.001)
$ [\![\boldsymbol{\beta}_{5\rightarrow 6}]\!]_{\{1\}} $	0.4	0.028 (0.005)	−0.106 (0.003)
$ [\![\boldsymbol{\beta}_{5\rightarrow 6}]\!]_{\{2\}} $	0.6	0.034 (0.005)	−0.197 (0.004)
$ [\![\boldsymbol{\beta}_{5\rightarrow 6}]\!]_{\{3\}} $	0.5	0.028 (0.011)	−0.469 (0.001)
$ [\![\boldsymbol{\beta}_{5\rightarrow 6}]\!]_{\{4\}} $	0.3	0.025 (0.011)	−0.282 (0.001)

In Simulation 2, $ [\![\boldsymbol{\beta}_{s_{1}\rightarrow s_{2}}]\!]_{\{1\}} $ and $ [\![\boldsymbol{\beta}_{s_{1}\rightarrow s_{2}}]\!]_{\{2\}} $ correspond to the longitudinal risk factors, while $ [\![\boldsymbol{\beta}_{s_{1}\rightarrow s_{2}}]\!]_{\{3\}} $ and $ [\![\boldsymbol{\beta}_{s_{1}\rightarrow s_{2}}]\!]_{\{4\}} $ correspond to the non-time-varying risk factors.

From Tables 1 and 2, we can see that the biases of the proposed method for estimating the parameters are generally less than MSM.

Additional details on Simulations 1 and 2 can be found in Section S7. The data generation procedures are described in Section S7.1. Section S7.2 presents the estimates of the remaining model parameters. Overall, the biases in the estimated longitudinal model parameters are small (Tables S2 and S3). Section S7.3 provides additional comparisons between a likelihood-based approach and a bootstrap method for statistical inference on $ \boldsymbol{\beta}_{s_{1}\rightarrow s_{2}} $. The results indicate that the bootstrap approach generally provides more reliable statistical inference for $ \boldsymbol{\beta}_{s_{1}\rightarrow s_{2}} $ (Tables S4 and S5). Section S8 presents additional simulation studies involving more complex multistate models. Section S8.1 examines a 9-state multistate model structured like Fig. 1 to mimic the model used in the data application in Section 4. In addition, this simulation study investigates a scenario in which certain longitudinal variables enter the model as a risk factor only after individuals reach more advanced states, as in the data application. Some parameter estimates reported in Table S6 exhibit noticeable bias, suggesting that estimation may become challenging in complex models with limited sample sizes, particularly for parameters associated with transitions involving near-terminal states. Nevertheless, the proposed method generally yields more reliable estimates than MSM. Section S8.2 considers a 3-state model with loops, under which the proposed estimation method performs well and yields reasonably accurate parameter estimates (Tables S8 and S9).

### 3.2. Dynamic predictions

Simulation studies are performed to evaluate the dynamic prediction algorithm and the validity of the measures for assessing the predictions. We simulate the test datasets using the same settings as in Simulations 1 and 2. For each simulation, additional $ N^{*}=500 $ individuals are simulated. We set $ t_{\mathrm{L}} $ and $ t_{\mathrm{P}} $ to take values that are multiples of 0.25. As described in Section 2.3, the prediction metrics $ \hat{\mathrm{AUC}}_{s_{e}}(t_{\mathrm{L}},t_{\mathrm{LP}}) $, $ \hat{\mathrm{BS}}_{s_{e}}(t_{\mathrm{L}},t_{\mathrm{LP}}) $, and $ \hat{\mathrm{BS}}_{\mathbb{V}}(t_{\mathrm{L}},t_{\mathrm{LP}}) $ are estimated assuming that the complete history of state transitions $ s_{i}(t) $ is known in the test dataset ($ i\,=\,N\,+\,1 , \ldots, N\,+\,N^{*} $). On the other hand, the prediction metrics $ \tilde{\mathrm{AUC}}_{s_{e}}(t_{\mathrm{L}},t_{\mathrm{LP}}) $, $ \tilde{\mathrm{BS}}_{s_{e}}(t_{\mathrm{L}},t_{\mathrm{LP}}) $, and $ \tilde{\mathrm{BS}}_{\mathbb{V}}(t_{\mathrm{L}},t_{\mathrm{LP}}) $ are estimated by inferring $ s_{i}(t) $ based on the observed data. The estimated prediction metrics are presented in Table 3. It can be seen that when $ t_{\mathrm{LP}} $ is fixed, the prediction metrics will generally improve with $ t_{\mathrm{L}} $ increases, indicating that predictions can be improved with the accumulation of follow-up data. The values $ \tilde{\mathrm{AUC}}_{s_{e}}(t_{\mathrm{L}},t_{\mathrm{LP}}) $, $ \tilde{\mathrm{BS}}_{s_{e}}(t_{\mathrm{L}},t_{\mathrm{LP}}) $ and $ \tilde{\mathrm{BS}}_{\mathbb{V}}(t_{\mathrm{L}},t_{\mathrm{LP}}) $ generally provide a good approximation of the corresponding values $ \hat{\mathrm{AUC}}_{s_{e}}(t_{\mathrm{L}},t_{\mathrm{LP}}) $, $ \hat{\mathrm{BS}}_{s_{e}}(t_{\mathrm{L}},t_{\mathrm{LP}}) $ and $ \hat{\mathrm{BS}}_{\mathbb{V}}(t_{\mathrm{L}},t_{\mathrm{LP}}) $, demonstrating a good performance of the weighting method.

**Table 3:** Prediction metrics for evaluating the performance of dynamic predictions in Simulations 1 and 2.

$ t_{\mathrm{L}} $	$ t_{\mathrm{LP}} $	$ \hat{\mathrm{AUC}}_{s_{e}}(t_{\mathrm{L}},t_{\mathrm{LP}}) $	$ \tilde{\mathrm{AUC}}_{s_{e}}(t_{\mathrm{L}},t_{\mathrm{LP}}) $	$ \hat{\mathrm{BS}}_{s_{e}}(t_{\mathrm{L}},t_{\mathrm{LP}}) $	$ \tilde{\mathrm{BS}}_{s_{e}}(t_{\mathrm{L}},t_{\mathrm{LP}}) $	$ \hat{\mathrm{BS}}_{\mathbb{V}}(t_{\mathrm{L}},t_{\mathrm{LP}}) $	$ \tilde{\mathrm{BS}}_{\mathbb{V}}(t_{\mathrm{L}},t_{\mathrm{LP}}) $
**Simulation 1**							
0.25	0.50	0.691 (0.002)	0.692 (0.002)	0.0967 (0.0005)	0.0967 (0.0004)	0.4731 (0.0010)	0.4729 (0.0009)
0.25	0.75	0.698 (0.001)	0.698 (0.001)	0.1744 (0.0004)	0.1743 (0.0004)	0.6145 (0.0007)	0.6143 (0.0007)
0.25	1.00	0.705 (0.001)	0.706 (0.001)	0.2121 (0.0004)	0.2120 (0.0004)	0.6216 (0.0007)	0.6216 (0.0007)
0.50	0.75	0.692 (0.002)	0.692 (0.002)	0.1298 (0.0005)	0.1296 (0.0005)	0.4723 (0.0010)	0.4719 (0.0010)
0.50	1.00	0.700 (0.001)	0.700 (0.001)	0.1985 (0.0004)	0.1983 (0.0004)	0.5871 (0.0008)	0.5870 (0.0008)
0.75	1.00	0.684 (0.002)	0.685 (0.002)	0.1519 (0.0005)	0.1516 (0.0005)	0.4660 (0.0011)	0.4656 (0.0011)
**Simulation 2**							
0.25	0.50	0.863 (0.001)	0.863 (0.001)	0.0624 (0.0004)	0.0622 (0.0003)	0.5884 (0.0009)	0.5882 (0.0009)
0.25	0.75	0.792 (0.001)	0.791 (0.001)	0.1261 (0.0004)	0.1262 (0.0004)	0.7272 (0.0006)	0.7271 (0.0006)
0.25	1.00	0.725 (0.001)	0.725 (0.001)	0.2044 (0.0004)	0.2044 (0.0004)	0.7065 (0.0007)	0.7066 (0.0007)
0.50	0.75	0.823 (0.001)	0.822 (0.001)	0.0886 (0.0004)	0.0886 (0.0004)	0.5810 (0.0009)	0.5804 (0.0009)
0.50	1.00	0.739 (0.001)	0.739 (0.001)	0.1914 (0.0004)	0.1913 (0.0004)	0.6898 (0.0006)	0.6895 (0.0006)
0.75	1.00	0.798 (0.001)	0.798 (0.001)	0.1510 (0.0005)	0.1509 (0.0005)	0.5824 (0.0009)	0.5819 (0.0009)

Values in parentheses indicate the corresponding standard errors estimated from 500 simulation replicates.

## 4. Real data application

In this section, the proposed method is applied to data from the TEDDY study, which is a prospective cohort study following children with elevated risks of T1D. As described in Section 1, study participants were followed for the development of GADA, IA2A, IAA, and ZnT8A, and the current dataset includes 384 participants with GADA as the first appearing autoantibody. The clinical diagnosis of T1D occurs when classic symptoms lead to testing that meets laboratory criteria (such as elevated fasting glucose, A1c, or random glucose in the presence of symptoms). We removed all laboratory measures within 30 days of T1D onset to exclude the values directly used in establishing the T1D diagnosis. The primary aim of the analysis is to describe the risks of developing autoantibodies and T1D in this cohort and predict the risks for new participants following GADA positivity. We considered a directed graph as presented in Fig. 1, where States 1 to 8 are autoantibody states representing all possible combinations of confirmed autoantibodies, and State 9 ($ s_{e}=9 $) is the terminal state representing the development of T1D. Each thin edge in the graph corresponds to the appearance of a newly added autoantibody. All autoantibody states can transition to the T1D state which is represented by a bold arrow. As discussed earlier, the state occupation information is observed with uncertainties due to interval censoring or missing visits. Here we list 3 scenarios that are present in this dataset. (1) Some participants were not tested for ZnT8A at every visit. When ZnT8A was detected after a long period without ZnT8A testing, we do not know when ZnT8A became positive. (2) There were cases where more than one autoantibody was added from one visit to the next, without information on which added autoantibody appeared first. (3) Missing autoantibody tests and lapses in follow-up visits may occur. There were participants with an extended period of no autoantibody follow-up.

The data include $ N_{y}=2 $ longitudinal variables: hemoglobin A1c (HbA1c) tests and 2-h glucose levels from oral glucose tolerance tests. Both are continuous measures of glucose levels. HbA1c levels were measured following the appearance of the first autoantibody, while 2-h glucose levels were only measured after the appearance of the second autoantibody. Linear trajectories are assumed. $ N_{z}=2 $ non-time-varying covariates include age at GADA positivity and the presence of DQ2/8 heterozygous genotype. State transition times and observation times are measured from the time origin of GADA positivity. The design interval extends from the time origin of GADA positivity to the last observed transition time or observation times and is partitioned into monthly subintervals with a total of $ N_{t}=178 $ subintervals. The characteristics of participants, a summary of variables at first visit following GADA positivity, and the number of observed state transitions are presented in Section S9.

The parameter estimates are reported from the model fitted to the entire dataset, while the predictions are assessed using 10-fold cross-validation with models trained on randomly partitioned subsets of the data. The model fit of the entire data took 2.0 h on a Linux-based supercomputing system with a 64-bit x86 architecture using Julia (version 1.6.7), while on the subsets for cross-validation, the run times ranged from 1.7 to 4.2 h. The estimated coefficients are presented in Table 4, with 95% confidence intervals (CIs) computed using the bootstrap method described in Section S7.3 [c.f., Hsieh et al. (2006)]. From this table, we can see that younger age at GADA positivity tends to be associated with an increased risk of developing autoantibodies. The genotype DQ2/8 is associated with the development of IA2A following single GADA positivity and the development of IAA following GADA and IA2A positivity. Higher glucose and HbA1c measures are more likely to be associated with increased risks of T1D.

**Table 4:** Estimated coefficients $ \boldsymbol{\beta}_{s_{1}\rightarrow s_{2}} $ and the corresponding bootstrap 95% CI in the real data application.

Variable	Coefficient	95% CI
**GADA** * → * **GADA IA2A**		
HbA1c	−0.789	(−3.727,1.349)
Age	−0.115	(−0.239,0.007)
DQ2/8	0.517	(−0.378,1.217)
**GADA** * → * **GADA IAA**		
HbA1c	0.248	(−1.081,1.498)
Age	−0.159	(−0.240,−0.094)
DQ2/8	0.584	(0.218,1.099)
**GADA** * → * **GADA ZnT8A**		
HbA1c	−0.729	(−2.168,1.748)
Age	−0.119	(−0.232,−0.048)
DQ2/8	0.099	(−0.622,0.663)
**GADA** * → * **T1D**		
HbA1c	7.948	(1.416,35.578)
Age	−0.201	(−1.930,0.012)
DQ2/8	−1.303	(−29.159,−0.239)
**GADA IA2A** * → * **GADA IA2A IAA**		
Log glucose	−3.238	(−29.896,11.311)
HbA1c	4.118	(−4.683,18.759)
Age	−0.180	(−1.960,0.457)
DQ2/8	1.333	(−0.194,30.751)
**GADA IA2A** * → * **GADA IA2A ZnT8A**		
Log glucose	0.406	(−11.629,11.621)
HbA1c	1.070	(−6.796,11.487)
Age	0.065	(−0.532,0.615)
DQ2/8	0.370	(−2.233,3.678)
**GADA IA2A** * → * **T1D**		
Log glucose	4.040	(−15.654,15.729)
HbA1c	5.871	(−9.360,24.874)
Age	−0.891	(−37.971,1.012)
DQ2/8	2.603	(−20.304,67.214)
**GADA IAA** * → * **GADA IA2A IAA**		
Log glucose	−2.066	(−10.040,7.130)
HbA1c	2.929	(−0.493,7.522)
Age	−0.195	(−0.577,−0.054)
DQ2/8	−0.309	(−1.475,0.967)
**GADA IAA** * → * **GADA IAA ZnT8A**		
Log glucose	−0.216	(−8.820,9.457)
HbA1c	−1.272	(−4.375,3.386)
Age	0.089	(−0.092,0.258)
DQ2/8	−0.793	(−2.023,0.272)
**GADA IAA** * → * **T1D**		
Log glucose	6.472	(−4.064,15.627)
HbA1c	10.226	(4.034,25.843)
Age	−0.069	(−2.441,1.549)
DQ2/8	−0.163	(−5.749,29.658)

Variable	Coefficient	95% CI
**GADA ZnT8A** * → * **GADA IA2A ZnT8A**		
Log glucose	5.371	(2.277,8.465)
HbA1c	−3.117	(−5.284,−0.950)
Age	−0.046	(−0.172,0.079)
DQ2/8	0.146	(−0.599,0.891)
**GADA ZnT8A** * → * **GADA IAA ZnT8A**		
Log glucose	−1.930	(−8.455,4.595)
HbA1c	−3.092	(−7.055,0.871)
Age	0.055	(−0.217,0.326)
DQ2/8	0.295	(−1.108,1.699)
**GADA ZnT8A** * → * **T1D**		
Log glucose	1.547	(−5.067,8.161)
HbA1c	17.444	(2.341,32.546)
Age	0.649	(−0.642,1.939)
DQ2/8	3.764	(−0.632,8.161)
**GADA IA2A IAA** * → * **GADA IA2A IAA ZnT8A**		
Log glucose	−5.274	(−8.438,−2.110)
HbA1c	3.499	(1.188,5.811)
Age	0.073	(−0.094,0.241)
DQ2/8	0.251	(−0.676,1.178)
**GADA IA2A IAA** * → * **T1D**		
Log glucose	12.054	(6.714,17.394)
HbA1c	−3.804	(−7.748,0.141)
Age	−0.075	(−0.375,0.226)
DQ2/8	−2.432	(−3.847,−1.017)
**GADA IA2A ZnT8A** * → * **GADA IA2A IAA ZnT8A**		
Log glucose	4.405	(0.084,8.726)
HbA1c	−1.253	(−3.871,1.364)
Age	−0.072	(−0.267,0.122)
DQ2/8	0.967	(−0.175,2.110)
**GADA IA2A ZnT8A** * → * **T1D**		
Log glucose	13.099	(6.926,19.272)
HbA1c	4.758	(0.206,9.311)
Age	0.066	(−0.273,0.406)
DQ2/8	0.392	(−0.872,1.655)
**GADA IAA ZnT8A** * → * **GADA IA2A IAA ZnT8A**		
Log glucose	2.226	(−0.625,5.077)
HbA1c	−1.183	(−2.944,0.578)
Age	0.055	(−0.068,0.178)
DQ2/8	0.383	(−0.384,1.149)
**GADA IAA ZnT8A** * → * **T1D**		
Log glucose	3.524	(−3.503,10.551)
HbA1c	9.035	(3.350,14.719)
Age	−0.701	(−1.413,0.012)
DQ2/8	−0.788	(−3.261,1.684)
**GADA IA2A IAA ZnT8A** * → * **T1D**		
Log glucose	7.515	(5.374,9.656)
HbA1c	1.486	(0.264,2.709)
Age	0.009	(−0.105,0.123)
DQ2/8	−0.062	(−0.678,0.554)

**Figure 4:**
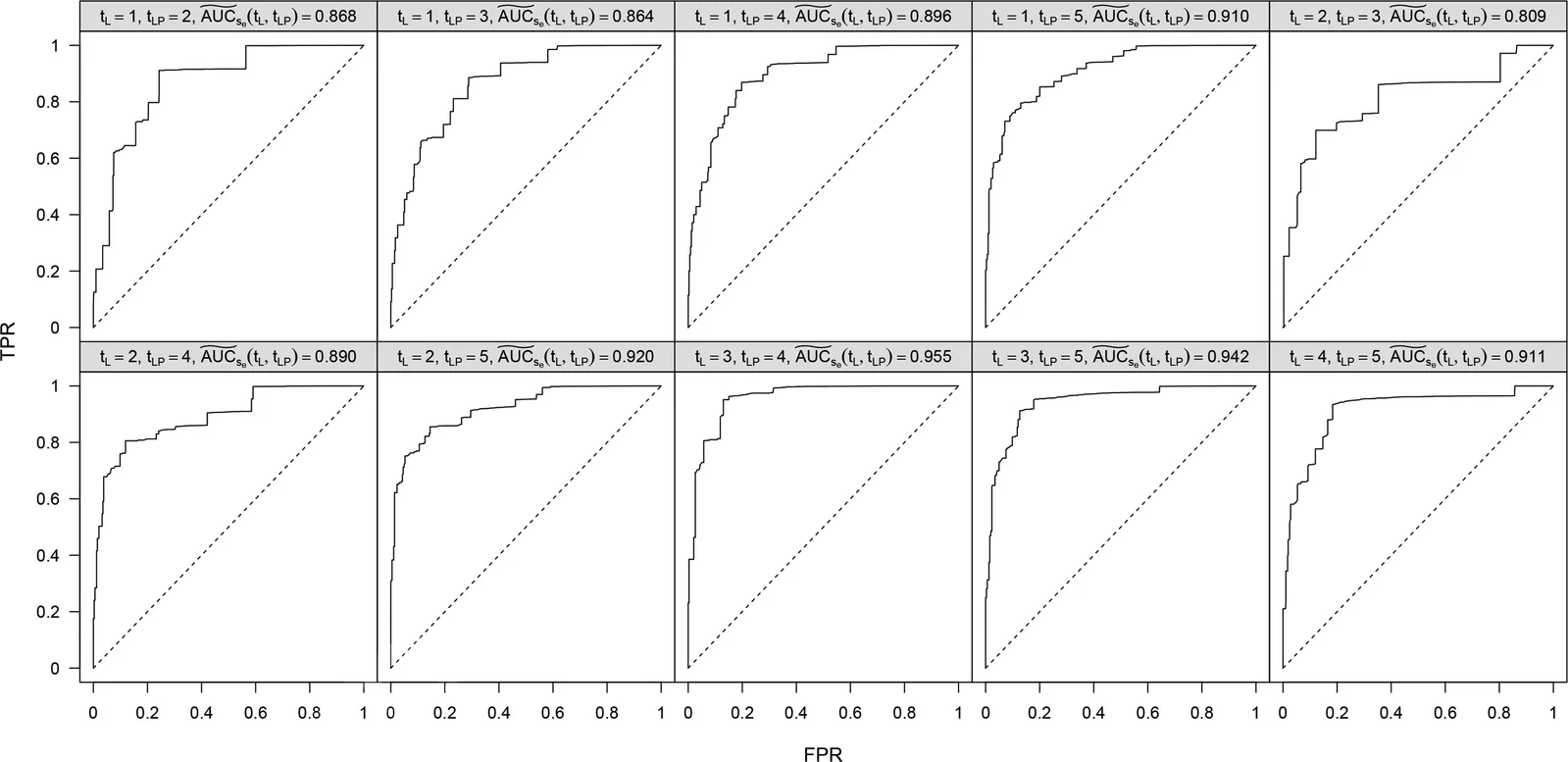
Dynamic ROC curves and the corresponding AUC in the TEDDY data application for various combinations of $ t_{\mathrm{L}} $ and $ t_{\mathrm{LP}} $ ($ s_{e}=9 $: T1D).

Next, we evaluated the dynamic prediction algorithm by cross-validation. A total of 384 participants were randomly divided into 10 folds with similar sizes for 10 iterations of evaluations. For each iteration, 1 fold was used as the test data for evaluating the predictions while the remaining 9 folds were used as training data for fitting the model. Figure 4 presents $ \tilde{\mathrm{ROC}}_{s_{e}}(t_{\mathrm{L}},t_{\mathrm{LP}}) $ and $ \tilde{\mathrm{AUC}}_{s_{e}}(t_{\mathrm{L}},t_{\mathrm{LP}}) $ for various combinations of $ t_{\mathrm{L}} $ and $ t_{\mathrm{LP}} $ where $ \tilde{\mathrm{AUC}}_{s_{e}}(t_{\mathrm{L}},t_{\mathrm{LP}}) $ ranged between 0.809 and 0.942. Table 5 presents $ \tilde{\mathrm{BS}}_{s_{e}}(t_{\mathrm{L}},t_{\mathrm{LP}}) $ and $ \tilde{\mathrm{BS}}(t_{\mathrm{L}},t_{\mathrm{LP}}) $ for various combinations of $ t_{\mathrm{L}} $ and $ t_{\mathrm{LP}} $, where $ t_{\mathrm{L}} $ is the landmark time and $ t_{\mathrm{P}}=t_{\mathrm{LP}}-t_{\mathrm{L}} $ is the prediction horizon. The results suggest that with a smaller landmark time $ t_{\mathrm{L}} $, the prediction tends to be compromised by insufficient observations. As $ t_{\mathrm{L}} $ increases, more historical observations are accumulated, and the predictions tend to improve. However, if $ t_{\mathrm{L}} $ becomes too large, we may miss the periods where most observations and events occur. When the prediction horizon $ t_{\mathrm{P}}=t_{\mathrm{LP}}-t_{\mathrm{L}} $ is small, the ROC curves tend to be less smooth for the limited numbers of disease outcomes within this short interval. However, extending the prediction horizon $ t_{\mathrm{P}} $ too far also makes predictions challenging. These evaluation metrics offer insights into the number of years of data needed to achieve reliable dynamic predictions and the extent to which the methods can produce meaningful forecasts. Based on the findings, we propose collecting 2 yr of data following GADA positivity to ensure more robust and accurate predictions from our model.

**Table 5:** Estimated dynamic BS in the TEDDY data application for various combinations of $ t_{\mathrm{L}} $ and $ t_{\mathrm{LP}} $ ($ s_{e}=9 $: T1D).

$ t_{\mathrm{L}} $	$ t_{\mathrm{LP}} $	$ \tilde{\mathrm{BS}}_{s_{e}}(t_{\mathrm{L}},t_{\mathrm{LP}}) $	$ \tilde{\mathrm{BS}}_{\mathbb{V}}(t_{\mathrm{L}},t_{\mathrm{LP}}) $
1	2	0.0297	0.425
1	3	0.0457	0.521
1	4	0.0593	0.548
1	5	0.0688	0.536
2	3	0.0243	0.283
2	4	0.0387	0.378
2	5	0.0510	0.408
3	4	0.0241	0.260
3	5	0.0466	0.324
4	5	0.0371	0.224

**Figure 5:**
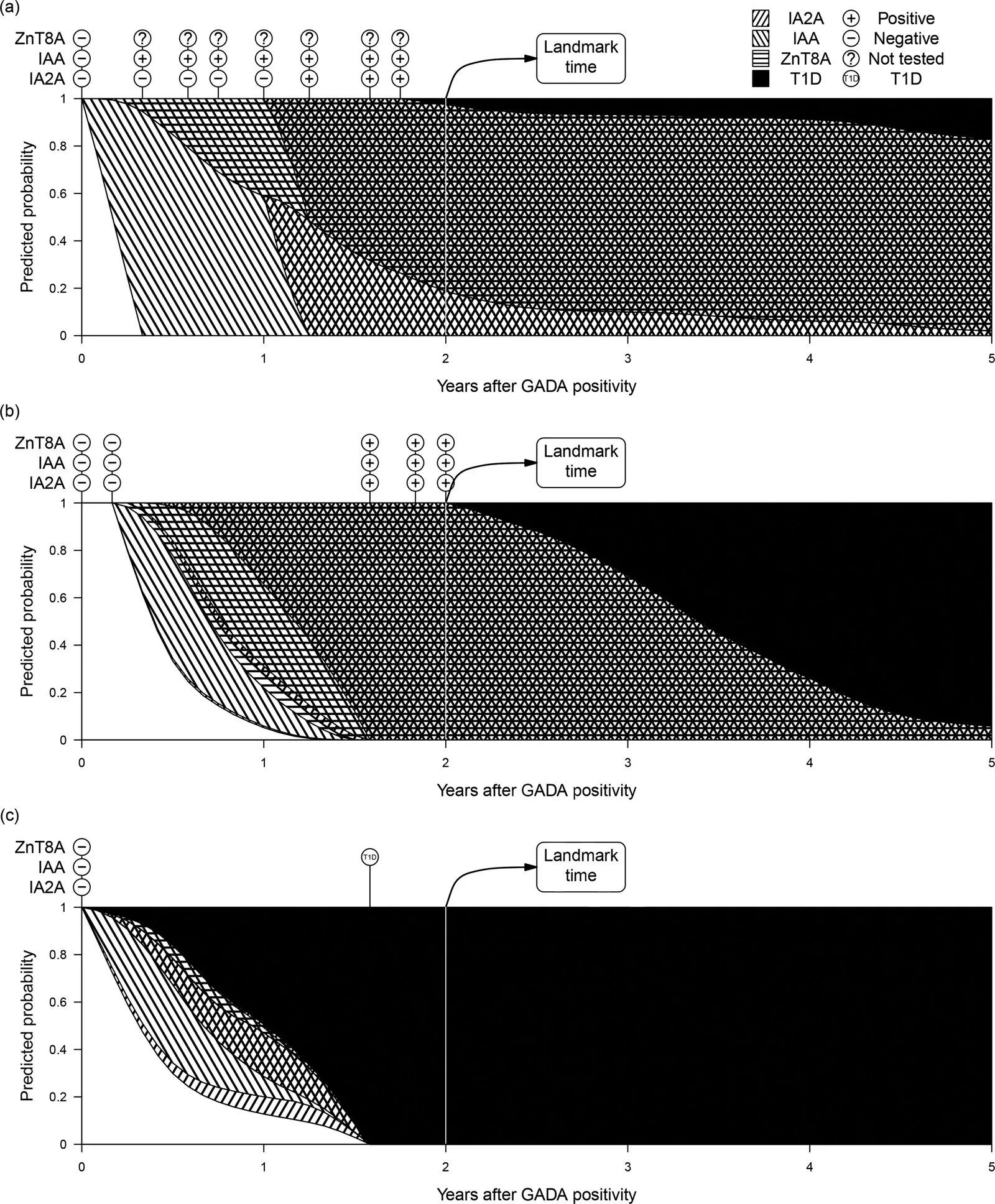
Three examples for illustrating the 3 scenarios in which the state occupation information was observed with uncertainty. Landmark time $ t_{L}=2 $ yr following GADA positivity. Probabilities refer to retrospective probabilities prior to 2 yr and predictive probabilities post 2 yr.

Finally, in Fig. 5, we will show some cases that illustrate the 3 scenarios in which the state occupation information was observed with uncertainty. The same graphs with longitudinal data points added are shown in Figs. S7 to S9. Data collected within the first 2 yr following GADA positivity were used for prediction of future state occupation between 2 and 5 yr ($ t_{\mathrm{L}}=2 $ and $ t_{\mathrm{LP}}\in(2,5] $). The dynamic predictions were made based on the models fitted with data from the other 9 folds that exclude these cases. Prior to the landmark time $ t_{\mathrm{L}} $, the probabilities represent the retrospective probabilities of past state occupation, while after the landmark time $ t_{\mathrm{L}} $, they represent the predictive probabilities of future state occupation. The first panel represents scenario (a). The participant was observed to have IA2A and IAA prior to the 2-yr landmark time, but ZnT8A was not tested at every visit. The algorithm suggested that the risk of having ZnT8A had been increasing due to IAA positivity, and by the landmark time, there was around 79% risk that ZnT8A had been positive. Future data confirmed that ZnT8A was tested positive around 2.5 yr after GADA positivity. This example also represents a case in which no longitudinal data were available for prediction at the landmark time $ t_{\mathrm{L}} $. Nevertheless, under the joint modeling framework, the model was able to make predictions of future state occupation based on the non-time-varying risk factors $ \mathbf{z}_{i} $, the observed state transitions, and the population-level longitudinal trajectory distribution. The second panel represents scenario (b). After the second visit following GADA positivity, there was a discontinuity in the participant’s follow-up for more than a year. When the participant returned to the study, the participant was additionally tested positive for IA2A, IAA, and ZnT8A, and we cannotfully determine which autoantibody occurred first. After fitting the dynamic prediction model with a 3-yr landmark time, the algorithm appeared to indicate a potential chronological sequence, with IAA most likely to occur first, followed by ZnT8A, and IA2A being the most probable to occur last. The third panel represents scenario (c). No autoantibody tests were conducted on the participant after GADA positivity. T1D diagnosis occurred roughly 1 yr and 7 mo afterward. The dynamic prediction model with a 2-yr landmark time suggested that there was a risk that the participant had a second autoantibody before T1D onset. At 6 mo, there was around 54% risk of multiple autoantibody positivity and 16% chance of T1D. At 1 yr, there was around 38% risk of multiple autoantibody positivity and 50% risk of T1D.

## 5. Discussion

In this manuscript, we presented a joint modeling approach for analyzing multivariate longitudinal and multistate data with an application to the characterization and prediction of autoantibody development concerned in the TEDDY study. Multiple challenges related to uncertainties in the observed data have been addressed, including measurement errors in longitudinal observations, incomplete state occupation information due to right censoring and interval censoring, and model performance evaluation in the presence of data uncertainties. Simulation studies demonstrate that the proposed method estimates the quantities of interest with reasonable precision. In the TEDDY application, the time-dependent AUC ranged between 0.809 and 0.942. Using a cohort of IA-positive children aged between 3 and 6 yr, a previous TEDDY model for T1D prediction based on logistic regression with only the baseline risk factors yielded an AUC of 0.80 (c.f., Jacobsen et al. 2019). Our results implied that improved prediction performance can be achieved by incorporating longitudinal data and history of autoantibody status. Incorporating additional genetic and metabolic risk factors has been shown in previous studies to improve performance in terms of AUC, though in different cohorts (eg Sosenko et al. 2013; Ferrat et al. 2020). Enhancing computational efficiency and expanding our model’s capacity to incorporate more predictors is therefore a promising direction to achieve more accurate predictions.

Our model is formulated in a general framework that accommodates a variety of multistate structures, and the proposed joint model falls under the category of current value parameterization. The model is constrained by the model assumptions, such as conditional independence of the longitudinal and multistate processes given the random effects, noninformative visit process, and a Markov assumption for the transition probabilities. The proposed statistical method still has room for improvement to enhance its reliability, practicality, and interpretability, as described briefly below. The multistate model we considered is a semiparametric model with a large number of parameters. Estimation of this model using the EM algorithm would be quite time-consuming when the directed graph $ (\mathbb{V},\mathbb{E}) $ of the model gets too complicated or when the dimension of covariates increases. It would be beneficial to explore methods to reduce the number of parameters without significantly compromising its flexibility. For example, the number of parameters in the coefficients $ \boldsymbol{\beta}_{s_{1}\rightarrow s_{2}} $ can be controlled by the least absolute shrinkage and selection operator by Tibshirani (1996) where nonessential coefficients can be shrunk to zero. The number of parameters in $ \mathbf{h}_{0, s_{1}\rightarrow s_{2}} $ can be reduced by approximating the functions with splines (c.f., Royston and Parmar 2002; Rizopoulos et al. 2009). The longitudinal model can also be made more flexible to accommodate specific requirements for modeling the trajectories. For example, Ferrer et al. (2016) considered a more flexible longitudinal model that allows the design matrices for the fixed and random effects to differ and allows the incorporation of additional covariates in modeling the longitudinal trajectories. Dantan et al. (2011) considered an application where change points can be present in the longitudinal trajectories. The EM algorithm described in Section 2.2 can be modified to fit such a model based on the estimation procedures in Ferrer et al. (2016). The proposed model is a shared-parameter model and may be biased when predictors are used for determining T1D diagnosis. Future work could develop more robust frameworks that explicitly model the diagnostic mechanism alongside the longitudinal processes to better account for these dependencies.

In the joint model, we assumed that all longitudinal and multistate data could be aligned to a unique time origin when occupying the initial state. In the TEDDY application, the time origin is the time of GADA positivity. In some other applications, the time origin may be obscured by delayed entry or left truncation. Although the framework could, in principle, be extended to accommodate left truncation, such an extension would be considerably more complex. In particular, conditioning on participants who remain event-free until study entry may induce selection on the random-effects distribution Van den Berg and Drepper (2016). The maximum likelihood procedure would also need to account for all possible initial states. These challenges substantially complicate the extension and warrant further investigation in future research. Also, alternative time origins can sometimes help better capture the risk of certain state transitions and trajectories of longitudinal risk factors. For instance, in a study on palliative care data by Li et al. (2013), the researchers chose to align longitudinal observations retrospectively to terminal events, while aligning survival times prospectively from the time of enrollment. Similarly, in analyses of HIV and stroke data (eg Foucher et al. 2005; Kapetanakis et al. 2013), a semi-Markov model is used, where transition risks are modeled based on the time elapsed since entering a new state. These different strategies for aligning observation times introduce additional complexities in model selection.

To facilitate statistical inference for the regression coefficients, we used a bootstrap procedure to obtain 95% CIs in the real data application, albeit at substantial computational cost. Because bootstrap methods may be impractical for large datasets, alternative approaches are needed. Prior research has documented underestimation of standard errors using partial likelihood (c.f., Hsieh et al. 2006), while inferences based on observed likelihood (c.f., You et al. 2024b) are also complicated by the form of observed likelihood. Developing or assessing alternative methods for obtaining standard errors remains an important direction for future research. Another limitation in our data application is that the T1D endpoint can be diagnosed based on the covariates (eg glucose and HbA1c), which may introduce bias according to Thomadakis et al. (2019). Methods to address informative dropouts can be considered to mitigate the biases in the estimation. Finally, the proposed method lacks diagnostic tools to assess model adequacy, as standard approaches typically require comparing observed and expected transitions (c.f., Titman and Sharples 2010). Because interval censoring and censored states obscure the true transition paths, these diagnostics cannot be directly applied. Developing diagnostic procedures tailored to this setting is therefore an important direction for future research.

In Section 2.3, we presented several metrics for evaluating the accuracy of dynamic predictions. In cases where the test dataset is also subject to interval censoring, we used the model-based approach by Beyene and El Ghouch (2022) to estimate these metrics. However, as noted by Blanche et al. (2015), a model-free approach is generally preferred when comparing different methods as the evaluations will not depend on the unbiasedness of estimates. Currently, there is still a lack of literature regarding model-free approaches for evaluating predictions of interval-censored data. Developing model-free techniques for estimation of the metrics will be an area of future research efforts. In addition, the proposed prediction metrics primarily focus on predicting entry into the end state, whereas there is increasing interest in evaluating prediction performance across a sequence of intermediate states using extensions of the AUC, particularly in T1D research, where disease onset is often characterized as progression from Stage 1 to Stage 3 T1D. Exploring such multistate prediction metrics represents an important direction for future work.

## Supplementary Material

kxag018_Supplementary_Data

## Data Availability

Data from The Environmental Determinants of Diabetes in the Young (https://doi.org/10.58020/y3jk-x087) reported here will be made available for request at the NIDDK Central Repository (NIDDK-CR) website, Resources for Research (R4R), https://repository.niddk.nih.gov/.
