# Supplementary material for: Risk estimation and dynamic prediction using discrete-time joint models for longitudinal and multistate data with interval and state censoring
Source: Biostatistics. 2026 Jul 6;27(1):kxag018. doi: 10.1093/biostatistics/kxag018 (PMC13337610; doi:10.1093/biostatistics/kxag018)
Supplement: kxag018_Supplementary_Data [file kxag018_supplementary_data.pdf]

Supplementary Materials for "Disease risk estimation and dynamic prediction based on joint modeling of multivariate longitudinal data and multistate data with uncertainties"

Contents

S1 Model Assumptions **3**

S1.1 Longitudinal model assumptions . . . 3

S1.2 Multistate model assumptions . . . 3

S1.3 Distribution assumptions on censoring and observation times . . . 4

S1.4 Assumptions on the discrete approximation . . . 5

S2 Full Details on Model Estimation **6**

S2.1 EM algorithm . . . 6

S2.2 Model convergence and diagnosis of non-convergence . . . 12

S2.2.1 Technique 1. Filtering Monte Carlo draws leading to zero/negative likelihood 13

S2.2.2 Technique 2. Refinement of the time discretization . . . 14

S2.2.3 Technique 3. On-the-fly interval splitting within the E-step . . . 14

S3 Additional Details on Dynamic Predictions **15**

S4 Considerations on Discrete Approximation **16**

S4.1 General consideration . . . 16

S4.2 Additional simulation studies to evaluate the impact of approximation on model estimation . . . 17

S5 Computational Considerations **18**

S5.1 Simplifying expressions for $\kappa^{g^\delta}_{i,j,s_1,s_2}(\mathbf{b}_i)$, $\kappa^{g}_{i,j,s_1}(\mathbf{b}_i)$, and $\kappa^{0}_{i}(\mathbf{b}_i)$ . . . 18

S5.2 A dynamic strategy to accelerate the estimation . . . 19

S6 Illustrating Examples **20**

S6.1 Illustrating Example 1 20

S6.2 Illustrating Example 2 22

S7 Additional Details and Results on Simulation Studies **23**

S7.1 Details on the generation of simulation data 23

S7.2 Additional results for model parameter estimates 23

S7.3 Methods for statistical inference on $\boldsymbol{\beta}_{s_1 \to s_2}$ 23

S8 Additional simulation studies **29**

S8.1 Simulation 3 29

S8.2 Simulation 4 31

S9 Additional Details on Real Data Application **33**

S9.1 Descriptive summary of TEDDY dataset 33

S9.2 Additional information on the results 34

S1 Model Assumptions

This section outlines all assumptions adopted for the model formulation as well as the discrete approximation scheme.

S1.1 Longitudinal model assumptions

In the longitudinal mixed effects model

$$\mathbf{y}_{ik} = \begin{pmatrix} m_{ik}(t_{ik1}) \\ \vdots \\ m_{ik}(t_{ikn_{ik}}) \end{pmatrix} + \begin{pmatrix} \epsilon_{ik1} \\ \vdots \\ \epsilon_{ikn_{ik}} \end{pmatrix} = \mathbf{X}_{ik}\mathbf{b}_{ik} + \boldsymbol{\epsilon}_{ik} = \mathbf{X}_{ik}\mathbf{c}_k + \mathbf{X}_{ik}\mathbf{a}_{ik} + \boldsymbol{\epsilon}_{ik},$$

ϵ_{ikj} is random noise following $N(0, \sigma_k^2)$ distribution. $\mathbf{a}_i = \left(\mathbf{a}_{i1}^\mathsf{T}, \ldots, \mathbf{a}_{iN_y}^\mathsf{T}\right)^\mathsf{T}$ follows a multivariate normal distribution $N(\mathbf{0}, \boldsymbol{\Sigma}_a)$. Basis functions $B_{kl}(t)$ and the longitudinal processes $\mathbf{m}_i(t)$ are Lipschitz continuous functions with bounded second derivatives in $[0, \mathcal{T}]$.

S1.2 Multistate model assumptions

The state occupation process of the ith individual $s_i(t)$ is modeled by the instantaneous risk of transitions. We assume that the instantaneous risk of transitioning from s_1 to s_2 ($s_1 \neq s_2$) is

$$Q_{i,s_1\to s_2}(t) = \lim_{\Delta t\to 0} P\big(s_i(t+\Delta t) = s_2 | s_i(t) = s_1\big)/\Delta t = h_{0,s_1\to s_2}(t) \exp\left\{\boldsymbol{\beta}_{s_1\to s_2}^\mathsf{T} \boldsymbol{\eta}_{i,s_1\to s_2}(t)\right\}$$

for $(s_1, s_2) \in \mathbb{E}$, and $Q_{i,s_1\to s_2}(t) = 0$ otherwise. By the definition of transition-risk matrices, we let $Q_{i,s_1\to s_1}(t) = -\sum_{s_2:s_2\neq s_1} Q_{i,s_1\to s_2}(t)$ denote the instantaneous risk of transitioning out of state s_1, so that each row of the matrix $\mathbf{Q}_i(t) = \{Q_{i,s_1\to s_2}(t)\}_{s_1=1\ldots,N_s,s_2=1,\ldots,N_s}$ sums up to 0. In this manuscript specifically, we assume that all individuals start in a common state (indexed by 1) at time 0. $h_{0,s_1\to s_2}(t)$ are positive and Lipschitz continuous functions bounded away from 0 on $[0, \mathcal{T}]$. The non-time-varying covariates $\mathbf{z}_i$ in the multistate model are independent and identically distributed with a finite moment $\mathrm{E}[\|\mathbf{z}_i\|^k]$ for $k \in [1, \infty)$. The distribution of the times entering the end state E_i is determined by the multistate model assumptions.

S1.3 Distribution assumptions on censoring and observation times

In this subsection, we introduce the distribution assumptions on censoring and observation times (visiting processes), including right-censored state occupation observations, interval-censored state occupation observations, and censored states.

The visiting processes generally follow the "visiting completely at random" assumption in Pullenayegum and Lim (2016). More specifically, let $N_i^{\{T_{ij}\}}(t)$ be the counting process of the state transition observation time sequences $T_{i1} < T_{i2} < T_{i3} < \ldots$, and $N_{ik}^{\{t_{ikj}\}}(t)$ be the counting process of the longitudinal observation time sequences $t_{ik1} < t_{ik2} < t_{ik3} < \ldots$. The actual observation time sequences are truncated by the last follow-up times O_i, which is usually the minimum of the censoring time C_i and the time entering an end state E_i. In other words, the observation time sequence $T_{i1} < T_{i2} < \ldots < T_{in_i}$ and $t_{ik1} < t_{ik2} < \ldots < t_{ikn_{ik}}$ are defined by the counting processes $N_i^{\{T_{ij}\}}(t \wedge O_i)$ and $N_{ik}^{\{t_{ikj}\}}(t \wedge O_i)$. We assume that $N_i^{\{T_{ij}\}}(t)$ are independent and identically distributed with intensity function $\lambda^{\{T_{ij}\}}(t) = \lim_{\Delta t \to 0} \mathrm{E}\left[N_i^{\{T_{ij}\}}(t+\Delta t) - N_i^{\{T_{ij}\}}(t)\right]\Big/\Delta t$ and $N_{ik}^{\{t_{ikj}\}}(t)$ are independent and identically distributed with intensity functions $\lambda_k^{\{t_{ikj}\}}(t) = \lim_{\Delta t \to 0} \mathrm{E}\left[N_{i,k}^{\{t_{ikj}\}}(t+\Delta t) - N_{i,k}^{\{t_{ikj}\}}(t)\right]\Big/\Delta t$ and $N_{ik}^{\{t_{ikj}\}}(t)$. $\lambda^{\{T_{ij}\}}(t)$ and $\lambda_k^{\{t_{ikj}\}}(t)$ are positive, continuous, and bounded away from 0 and ∞ in the design interval $[0, \mathcal{T}]$. $N_i^{\{T_{ij}\}}(t)$ and $N_{ik}^{\{t_{ikj}\}}(t)$ are non-informative of longitudinal and state occupation information, such that $N_i^{\{T_{ij}\}}(t)$ and $N_{ik}^{\{t_{ikj}\}}(t)$ are independent of longitudinal and state occupation data as well as O_i, C_i and E_i, and do not depend on the parameters underlying the generation of longitudinal and state occupation data, (non-informative visiting time processes assumption). We also assume that state censoring is non-informative such that conditional on the true state occupation $s_i(T_{ij})$, the probability of observing S_{ij} depends only on whether S_{ij} contains $s_i(T_{ij})$ and does not otherwise depend on other parameters and observations (non-informative state censoring)

$$\mathrm{P}\left(S_{ij} \middle| \{s_i(t)\}_{t\in[0,\mathcal{T}]}; \boldsymbol{\Theta}_\sigma, \boldsymbol{\Theta}_{\beta,h}, \boldsymbol{\Theta}_{c,\Sigma}\right) = \mathrm{P}\left(S_{ij} \middle| s_i(T_{ij}) \in S_{ij}\right)$$

or equivalently, based on the discrete approximation

$$\mathrm{P}\left(S_{ij} \middle| \mathcal{A}_i; \boldsymbol{\Theta}_\sigma, \boldsymbol{\Theta}_{\beta,h}, \boldsymbol{\Theta}_{c,\Sigma}\right) = \mathrm{P}\left(S_{ij} \middle| s_i(T_{ij}) \in S_{ij}\right).$$

S1.4 Assumptions on the discrete approximation

In this manuscript, a discrete approximation scheme is implemented to facilitate model estimation. We assume that there exists a partition of the design interval $(0, \mathcal{T}] = \bigcup_{j=1}^{N_t} \mathfrak{I}_j$ where $\mathfrak{I}_1 = (\tau_0, \tau_1], \mathfrak{I}_2 = (\tau_1, \tau_2], \ldots, \mathfrak{I}_{N_t} = (\tau_{N_t-1}, \tau_{N_t}]$. Let $\mathbb{T} = \{\tau_0, \ldots, \tau_{N_t}\}$ be the time sequence of all splitting points and $\widetilde{\tau}_q = (\tau_q + \tau_{q-1})/2$ be the mid-point of the interval $\mathfrak{I}_q$. We expect that $\mathbb{T}$ is sufficiently dense in the interval $(0, \mathcal{T}]$ (i.e., $|\tau_j - \tau_{j-1}|$ is small enough) such that the following approximations will not result in large biases in the estimates:

- State transition observation times T_{ij} can be approximated by certain $T_{ij}^{\circ} \in \mathbb{T}$, such that $|T_{ij}^{\circ} - T_{ij}|$ is sufficiently small.
- $\mathbf{m}_i(t)$ can be approximated by $\mathbf{m}_i(\widetilde{\tau}_j) = \mathbf{m}_i(\mathfrak{I}_j)$ in the interval $\mathfrak{I}_j$. By the Lipschitz continuity of $\mathbf{m}_i(t)$, this requires $|\tau_j - \tau_{j-1}|$ to be sufficiently small.
- For $(s_1, s_2) \in \mathbb{E}$,

$$\begin{aligned}\mathrm{P}\left(s_i(\tau_j) = s_2 \middle| s_i(\tau_{j-1}) = s_1\right) &= \mathrm{P}\left(\delta_{i,s_1\to s_2}(\mathfrak{I}_j) = 1 \middle| g_{i,s_1}(\mathfrak{I}_j) = 1\right) \\ &\approx 1 - \exp\Big\{ - h_{0,s_1\to s_2}(\mathfrak{I}_j) \exp\left[\boldsymbol{\beta}_{s_1\to s_2}^{\mathsf{T}} \boldsymbol{\eta}_{i,s_1\to s_2}(\mathfrak{I}_j)\right]\Big\},\end{aligned}$$

  where the parameters $h_{0,s_1\to s_2}(\mathfrak{I}_j)$ can be interpreted as a discrete parameterization of $h_{0,s_1\to s_2}(t)$ in the interval $\mathfrak{I}_j$. Let $\mathbf{P}_{i,\mathfrak{I}_j} = \big\{ \mathrm{P}\left(s_i(\tau_j) = s_2 | s_i(\tau_{j-1}) = s_1\right)\big\}_{s_1=1\ldots,n_s, s_2=1\ldots,n_s}$ be the transition matrix in the interval $\mathfrak{I}_j$ for the ith individual. By noting that

$$\mathbf{P}_{i,\mathfrak{I}_j} \approx \mathbf{I} + (\tau_j - \tau_{j-1})\mathbf{Q}_i(t) + O(|\tau_j - \tau_{j-1}|^2),$$

  and that

$$\begin{aligned}&1 - \exp\Big\{ - h_{0,s_1\to s_2}(\mathfrak{I}_j) \exp\left[\boldsymbol{\beta}_{s_1\to s_2}^{\mathsf{T}} \boldsymbol{\eta}_{i,s_1\to s_2}(\mathfrak{I}_j)\right]\Big\} \\ &\qquad \approx h_{0,s_1\to s_2}(\mathfrak{I}_j) \exp\left[\boldsymbol{\beta}_{s_1\to s_2}^{\mathsf{T}} \boldsymbol{\eta}_{i,s_1\to s_2}(\mathfrak{I}_j)\right] + O\Big(\left|h_{0,s_1\to s_2}(\mathfrak{I}_j)\right|^2\Big)\end{aligned}$$

  The approximation suggests the following interpretation of the parameters

$$h_{0,s_1\to s_2}(\mathfrak{I}_j) \approx (\tau_j - \tau_{j-1}) h_{0,s_1\to s_2}(t)$$

  and can hold true when $|\tau_j - \tau_{j-1}|$ is sufficiently small.

S2 Full Details on Model Estimation

S2.1 EM algorithm

In this section, we present the full details of the model estimation, including the derivation of the formulas. Although some of this material overlaps with the main manuscript, we repeat the steps here for completeness, as the main text was constrained by length.

Without the data augmentation using $\mathcal{A}_i$, the probability density/mass functions of data are written as

$$L(\boldsymbol{\Theta}_\sigma, \boldsymbol{\Theta}_{\beta,h}, \boldsymbol{\Theta}_{c,\Sigma}) = \prod_{i=1}^{N} \int_{\Omega_{\mathbf{b}_i}} f\big(\mathcal{S}_i \big| \mathbf{b}_i; \boldsymbol{\Theta}_{\beta,h}\big) f\big(\mathcal{Y}_i \big| \mathbf{b}_i; \boldsymbol{\Theta}_\sigma\big) f\big(\mathbf{b}_i \big| \boldsymbol{\Theta}_{c,\Sigma}\big)\, d\mathbf{b}_i.$$

where

$$\log f(\mathcal{Y}_i|\mathbf{b}_i;\boldsymbol{\Theta}_\sigma) = \sum_{k=1}^{N_y}\left[-\frac{1}{2\sigma_k^2}(\mathbf{y}_{ik} - \mathbf{X}_{ik}\mathbf{b}_{ik})^\intercal(\mathbf{y}_{ik} - \mathbf{X}_{ik}\mathbf{b}_{ik}) - \frac{n_{ik}}{2}\log\{2\pi\sigma_k^2\}\right]$$
$$\log f(\mathbf{b}_i|\boldsymbol{\Theta}_{c,\Sigma}) = -\frac{1}{2}(\mathbf{b}_i - \mathbf{c})^\intercal \boldsymbol{\Sigma}_a^{-1}(\mathbf{b}_i - \mathbf{c}) - \frac{1}{2}\log\det\{2\pi\boldsymbol{\Sigma}_a\}.$$

and

$$\log f(\mathcal{S}_i|\mathbf{b}_i;\boldsymbol{\Theta}_{\beta,h}) = \log\left\{[\![\mathbf{1}]\!]_{S_{i0}}^\intercal\left[\prod_{j=1}^{n_i}[\![\mathbf{P}_{i,(T_{i,j-1},T_{ij}]}(\mathbf{b}_i;\boldsymbol{\Theta}_{\beta,h})]\!]_{S_{i,j-1},S_{ij}}\right][\![\mathbf{1}]\!]_{S_{in_i}}\right\},$$

where $\mathbf{1}$ is the 1-vector of length N_s, $\prod$ denotes matrix multiplication defaulting to left-to-right order, the (s_1, s_2)-th entry of $\mathbf{P}_{i,\mathfrak{I}_j}(\mathbf{b}_i;\boldsymbol{\Theta}_{\beta,h})$ is

$$[\![\mathbf{P}_{i,\mathfrak{I}_j}(\mathbf{b}_i;\boldsymbol{\Theta}_{\beta,h})]\!]_{\{s_1\},\{s_2\}} = \begin{cases} \mathrm{P}\left(\delta_{i,s_1\to s_2}(\mathfrak{I}_j) = 1 \middle| g_{i,s_1}(\mathfrak{I}_j) = 1\right) & s_1 \neq s_2 \\ 1 - \sum_{s_2:s_2\neq s_1}\mathrm{P}\left(\delta_{i,s_1\to s_2}(\mathfrak{I}_j) = 1 \middle| g_{i,s_1}(\mathfrak{I}_j) = 1\right) & s_1 = s_2 \end{cases}, \quad \text{(S1)}$$

and $\mathbf{P}_{i,(t_1,t_2]}(\mathbf{b}_i) = \prod_{\mathfrak{I}_j\subset(t_1,t_2]}\mathbf{P}_{i,\mathfrak{I}_j}(\mathbf{b}_i)$. Under this specification, it is quite difficult to compute the derivatives of $\log f(\mathcal{S}_i|\mathbf{b}_i;\boldsymbol{\Theta}_{\beta,h})$ with respect to parameters. We remark that since we let $\mathcal{S}_i$ and $\mathcal{Y}_i$ include observation times T_{ij} and t_{ikj}, $\log f(\mathcal{Y}_i|\mathbf{b}_i;\boldsymbol{\Theta}_\sigma)$ and $\log f(\mathcal{S}_i|\mathbf{b}_i;\boldsymbol{\Theta}_{\beta,h})$ should be calculated more precisely to include probability densities for $\{T_{ij}\}$ and t_{ikj}, i.e.,

$$\log f(\mathcal{Y}_i|\mathbf{b}_i;\boldsymbol{\Theta}_\sigma) = \log f\left(\{y_{ikj}\}_{k=1}^{N_y}{}_{j=1}^{n_{ik}}\middle|\{t_{ikj}\}_{k=1}^{N_y}{}_{j=1}^{n_{ik}}, \mathbf{b}_i;\boldsymbol{\Theta}_\sigma\right) + \log f\big(\{t_{ikj}\}_{k=1}^{N_y}{}_{j=1}^{n_{ik}}\big)$$
$$\log f(\mathcal{S}_i|\mathbf{b}_i;\boldsymbol{\Theta}_{\beta,h}) = \log f\left(\{S_{ij}\}_{j=1}^{n_i}\middle|\{T_{ij}\}_{j=1}^{n_i}, \mathbf{b}_i;\boldsymbol{\Theta}_{\beta,h}\right) + \log f\big(\{T_{ij}\}_{j=1}^{n_i}\big)$$

where these equations hold under the non-informative and independent visiting time assumptions outlined in Section S1.3 of the Supplementary Materials and $\log f\left(\{t_{ikj}\}_{k=1}^{N_y}{}_{j=1}^{n_{ik}}\right)$ and $\log f\left(\{T_{ij}\}_{j=1}^{n_i}\right)$ should not depend on any parameters also by the same assumptions. Therefore, it is safe to omit the quantities and treat them as constants in our presentation.

As described in the manuscript, the inference using the EM algorithm is primarily based on the following complete log-likelihood function with the augmented data $\mathcal{A}_i$

$$l_c(\mathbf{\Theta}_\sigma, \mathbf{\Theta}_{\beta,h}, \mathbf{\Theta}_{c,\Sigma}) = \sum_{i=1}^{N} \Big[\log f\big(\mathcal{A}_i, \mathcal{S}_i \big| \mathbf{b}_i; \mathbf{\Theta}_{\beta,h}\big) + \log f\big(\mathcal{Y}_i \big| \mathbf{b}_i; \mathbf{\Theta}_\sigma\big) + \log f\big(\mathbf{b}_i \big| \mathbf{\Theta}_{c,\Sigma}\big) \Big].$$

where

$$\begin{aligned}
&\log f(\mathcal{A}_i, \mathcal{S}_i | \mathbf{b}_i; \mathbf{\Theta}_{\beta,h}) = \log f(\mathcal{A}_i | \mathbf{b}_i; \mathbf{\Theta}_{\beta,h}) + \log f(\mathcal{S}_i | \mathcal{A}_i, \mathbf{b}_i) = \\
&\sum_{j=1}^{N_t} \sum_{(s_1,s_2)\in\mathbb{E}} g_{i,s_1}(\mathfrak{I}_j) \Bigg[\delta_{i,s_1\to s_2}(\mathfrak{I}_j) \log \Big\{ \exp\big\{ h_{0,s_1\to s_2}(\mathfrak{I}_j) \exp\big(\boldsymbol{\beta}_{s_1\to s_2}^{\mathsf{T}} \boldsymbol{\eta}_{i,s_1\to s_2}(\mathfrak{I}_j) \big) \big\} - 1 \Big\} \\
&- h_{0,s_1\to s_2}(\mathfrak{I}_j) \exp\big\{ \boldsymbol{\beta}_{s_1\to s_2}^{\mathsf{T}} \boldsymbol{\eta}_{i,s_1\to s_2}(\mathfrak{I}_j) \big\} \Bigg] + \log f(\mathcal{S}_i | \mathcal{A}_i, \mathbf{b}_i)
\end{aligned}$$

and $f(\mathcal{S}_i|\mathcal{A}_i, \mathbf{b}_i)$ does not contain information about any parameters by the non-informative censored state assumption. You et al. (2024) adopted the following first-order approximation to $\log f(\mathcal{A}_i, \mathcal{S}_i|\mathbf{b}_i; \mathbf{\Theta}_{\beta,h})$

$$\begin{aligned}
&\log f^{(1)}(\mathcal{A}_i, \mathcal{S}_i | \mathbf{b}_i; \mathbf{\Theta}_{\beta,h}) = \\
&\sum_{j=1}^{N_t} \sum_{(s_1,s_2)\in\mathbb{E}} g_{i,s_1}(\mathfrak{I}_j) \Bigg[\delta_{i,s_1\to s_2}(\mathfrak{I}_j) \big\{ \log h_{0,s_1\to s_2}(\mathfrak{I}_j) + \boldsymbol{\beta}_{s_1\to s_2}^{\mathsf{T}} \boldsymbol{\eta}_{i,s_1\to s_2}(\mathfrak{I}_j) \big\} \\
&- h_{0,s_1\to s_2}(\mathfrak{I}_j) \big(1 - \delta_{i,s_1\to s_2}(\mathfrak{I}_j)/2\big) \exp\big\{ \boldsymbol{\beta}_{s_1\to s_2}^{\mathsf{T}} \boldsymbol{\eta}_{i,s_1\to s_2}(\mathfrak{I}_j) \big\} \Bigg] + \log f(\mathcal{S}_i | \mathcal{A}_i, \mathbf{b}_i).
\end{aligned}$$

and the complete log-likelihood can be rewritten as

$$l_c^{(1)}(\mathbf{\Theta}_\sigma, \mathbf{\Theta}_{\beta,h}, \mathbf{\Theta}_{c,\Sigma}) = \sum_{i=1}^{N} \Big[\log f^{(1)}\big(\mathcal{A}_i, \mathcal{S}_i \big| \mathbf{b}_i; \mathbf{\Theta}_{\beta,h}\big) + \log f\big(\mathcal{Y}_i \big| \mathbf{b}_i; \mathbf{\Theta}_\sigma\big) + \log f\big(\mathbf{b}_i \big| \mathbf{\Theta}_{c,\Sigma}\big) \Big]. \quad \text{(S2)}$$

You et al. (2024) further shows that the nuisance parameters $h_{0,s_1\to s_2}(\mathfrak{I}_j)$ can be profiled out and a

profile likelihood specification can be obtained. Note that when $l_c^{(1)}(\boldsymbol{\Theta}_\sigma, \boldsymbol{\Theta}_{\beta,h}, \boldsymbol{\Theta}_{c,\Sigma})$ is maximized,

$$\begin{aligned}
0 &= \frac{\partial l_c^{(1)}(\boldsymbol{\Theta}_\sigma, \boldsymbol{\Theta}_{\beta,h}, \boldsymbol{\Theta}_{c,\Sigma})}{\partial h_{0,s_1\to s_2}(\Im_j)} \\
&= \frac{\sum_{i=1}^N g_{i,s_1}(\Im_j)\delta_{i,s_1\to s_2}(\Im_j)}{h_{0,s_1\to s_2}(\Im_j)} - \sum_{i=1}^N \big(1-\delta_{i,s_1\to s_2}(\Im_j)/2\big)\exp\big\{\boldsymbol{\beta}_{s_1\to s_2}^\mathsf{T}\boldsymbol{\eta}_{i,s_1\to s_2}(\Im_j)\big\}
\end{aligned}$$

and thus

$$h_{0,s_1\to s_2}(\Im_j) = \frac{\sum_{i=1}^N g_{i,s_1}(\Im_j)\delta_{i,s_1\to s_2}(\Im_j)}{\sum_{i=1}^N \big(1-\delta_{i,s_1\to s_2}(\Im_j)/2\big)\exp\big\{\boldsymbol{\beta}_{s_1\to s_2}^\mathsf{T}\boldsymbol{\eta}_{i,s_1\to s_2}(\Im_j)\big\}} \tag{S3}$$

By substituting (S3) to (S2), the first term of $l_c^{(1)}(\boldsymbol{\Theta}_\sigma, \boldsymbol{\Theta}_{\beta,h}, \boldsymbol{\Theta}_{c,\Sigma})$ can be rewritten as a partial likelihood when summed over all individuals:

$$\begin{aligned}
&\sum_{i=1}^N \log f^{(1)}(\mathcal{A}_i, \mathcal{S}_i|\mathbf{b}_i;\boldsymbol{\Theta}_{\beta,h}) = \\
&\quad \sum_{i=1}^N\sum_{j=1}^{N_t}\sum_{(s_1,s_2)\in\mathbb{E}} g_{i,s_1}(\Im_j)\Big[\delta_{i,s_1\to s_2}(\Im_j)\big\{\log h_{0,s_1\to s_2}(\Im_j) + \boldsymbol{\beta}_{s_1\to s_2}^\mathsf{T}\boldsymbol{\eta}_{i,s_1\to s_2}(\Im_j)\big\} \\
&\quad - h_{0,s_1\to s_2}(\Im_j)\big(1-\delta_{i,s_1\to s_2}(\Im_j)/2\big)\exp\big\{\boldsymbol{\beta}_{s_1\to s_2}^\mathsf{T}\boldsymbol{\eta}_{i,s_1\to s_2}(\Im_j)\big\}\Big] + \sum_{i=1}^N f(\mathcal{S}_i|\mathcal{A}_i,\mathbf{b}_i) \\
&= pl^{(1)}(\boldsymbol{\Theta}_{\beta,h}) = \sum_{i=1}^N\sum_{j=1}^{N_t}\sum_{(s_1,s_2)\in\mathbb{E}} g_{i,s_1}(\Im_j)\delta_{i,s_1\to s_2}(\Im_j)\Big\{\boldsymbol{\beta}_{s_1\to s_2}^\mathsf{T}\boldsymbol{\eta}_{i,s_1\to s_2}(\Im_j) \\
&\quad - \log\Big(\sum_{l=1}^N g_{l,s_1}(\Im_j)\big(1-\delta_{l,s_1\to s_2}(\Im_j)/2\big)\exp\big\{\boldsymbol{\beta}_{s_1\to s_2}^\mathsf{T}\boldsymbol{\eta}_{l,s_1\to s_2}(\Im_j)\big\}\Big)\Big\},
\end{aligned} \tag{S4}$$

We will omit the term $f(\mathcal{S}_i|\mathcal{A}_i,\mathbf{b}_i)$ when there is no ambiguity, as by the assumption of non-informative censoring, it does not involve any parameters.

By the theory of the EM algorithm, the parameters need to be updated iteratively to maximize the expected complete log-likelihood $\widetilde{\mathrm{E}}[l_c^{(1)}(\boldsymbol{\Theta}_\sigma, \boldsymbol{\Theta}_{\beta,h}, \boldsymbol{\Theta}_{c,\Sigma})]$ that can be written as follows

$$\begin{aligned}
&\widetilde{\mathrm{E}}[l_c^{(1)}(\boldsymbol{\Theta}_\sigma, \boldsymbol{\Theta}_{\beta,h}, \boldsymbol{\Theta}_{c,\Sigma})] \\
&\quad = \widetilde{\mathrm{E}}\Big[\sum_{i=1}^N \log f^{(1)}\big(\mathcal{A}_i, \mathcal{S}_i\big|\mathbf{b}_i;\boldsymbol{\Theta}_{\beta,h}\big)\Big] + \widetilde{\mathrm{E}}\Big[\sum_{i=1}^N \log f\big(\mathcal{Y}_i\big|\mathbf{b}_i;\boldsymbol{\Theta}_\sigma\big)\Big] + \widetilde{\mathrm{E}}\Big[\sum_{i=1}^N \log f\big(\mathbf{b}_i\big|\boldsymbol{\Theta}_{c,\Sigma}\big)\Big],
\end{aligned}$$

$$
\begin{aligned}
&\widetilde{\mathrm{E}}\Bigg[\sum_{i=1}^{N}\log f^{(1)}(\mathcal{A}_i,\mathcal{S}_i|\mathbf{b}_i;\boldsymbol{\Theta}_{\beta,h})\Bigg]\\
&\quad=\sum_{i=1}^{N}\sum_{j=1}^{N_t}\sum_{(s_1,s_2)\in\mathbb{E}}\Big[\widetilde{\mathrm{E}}_i[g_{i,s_1}(\mathfrak{I}_j)\delta_{i,s_1\to s_2}(\mathfrak{I}_j)]\log h_{0,s_1\to s_2}(\mathfrak{I}_j)\\
&\qquad+\boldsymbol{\beta}_{s_1\to s_2}^{\mathsf{T}}\widetilde{\mathrm{E}}_i[g_{i,s_1}(\mathfrak{I}_j)\delta_{i,s_1\to s_2}(\mathfrak{I}_j)\boldsymbol{\eta}_{i,s_1\to s_2}(\mathfrak{I}_j)]\\
&\qquad-\widetilde{\mathrm{E}}_i\big[g_{i,s_1}(\mathfrak{I}_j)h_{0,s_1\to s_2}(\mathfrak{I}_j)\big(1-\delta_{i,s_1\to s_2}(\mathfrak{I}_j)/2\big)\exp\big\{\boldsymbol{\beta}_{s_1\to s_2}^{\mathsf{T}}\boldsymbol{\eta}_{i,s_1\to s_2}(\mathfrak{I}_j)\big\}\big]\Big]
\end{aligned}
$$

$$
\widetilde{\mathrm{E}}\Bigg[\sum_{i=1}^{N}\log f(\mathcal{Y}_i|\mathbf{b}_i;\boldsymbol{\Theta}_{\sigma})\Bigg]=\sum_{i=1}^{N}\sum_{k=1}^{N_y}\Bigg[-\frac{1}{2\sigma_k^2}\widetilde{\mathrm{E}}_i\big[(\mathbf{y}_{ik}-\mathbf{X}_{ik}\mathbf{b}_{ik})^{\mathsf{T}}(\mathbf{y}_{ik}-\mathbf{X}_{ik}\mathbf{b}_{ik})\big]-\frac{n_{ik}}{2}\log\{2\pi\sigma_k^2\}\Bigg],
$$

and

$$
\widetilde{\mathrm{E}}\Bigg[\sum_{i=1}^{N}\log f(\mathbf{b}_i|\boldsymbol{\Theta}_{c,\Sigma})\Bigg]=\sum_{i=1}^{N}\Bigg[-\frac{1}{2}\widetilde{\mathrm{E}}_i\big[(\mathbf{b}_i-\mathbf{c})^{\mathsf{T}}\boldsymbol{\Sigma}_a^{-1}(\mathbf{b}_i-\mathbf{c})\big]-\frac{1}{2}\log\det\{2\pi\boldsymbol{\Sigma}_a\}\Bigg].
$$

By differentiating $\widetilde{\mathrm{E}}[l_c^{(1)}(\boldsymbol{\Theta}_{\sigma},\boldsymbol{\Theta}_{\beta,h},\boldsymbol{\Theta}_{c,\Sigma})]$ with respect to the parameters, we can obtain the following parameter updating procedure:

$$
\begin{aligned}
\mathbf{c}&\leftarrow\sum_{i=1}^{N}\widetilde{\mathrm{E}}_i[\mathbf{b}_i]/N\\
\boldsymbol{\Sigma}_a&\leftarrow\sum_{i=1}^{N}\widetilde{\mathrm{E}}_i[(\mathbf{b}_i-\mathbf{c})^{\otimes 2}]/N\\
\sigma_k^2&\leftarrow\sum_{i=1}^{N}\sum_{j=1}^{n_{ik}}\widetilde{\mathrm{E}}_i[(y_{ikj}-\mathbf{b}_{ik}^{\mathsf{T}}\mathbf{B}_k(t_{ikj}))^2]\Big/\sum_{i=1}^{N}n_{ik}\\
\boldsymbol{\beta}_{s_1\to s_2}&\leftarrow\boldsymbol{\beta}_{s_1\to s_2}-\left[\frac{\partial^2\widetilde{epl}}{(\partial\boldsymbol{\beta}_{s_1\to s_2})^2}\right]^{-1}\left[\frac{\partial\widetilde{epl}}{\partial\boldsymbol{\beta}_{s_1\to s_2}}\right]\\
h_{0,s_1\to s_2}(\mathfrak{I}_j)&\leftarrow\sum_{i=1}^{N}\widetilde{\mathrm{E}}_i[g_{i,s_1}(\mathfrak{I}_j)\delta_{i,s_1\to s_2}(\mathfrak{I}_j)]\Big/A_{s_1\to s_2}(\mathfrak{I}_j),
\end{aligned}
$$

where $\widetilde{epl}$ is the expected partial log-likelihood based on (S4), and

$$
\begin{aligned}
A_{s_1\to s_2}(\mathfrak{I}_j) &= \sum_{i=1}^{N} \widetilde{\mathrm{E}}_i\left[g_{i,s_1}(\mathfrak{I}_j)\big(1-\delta_{i,s_1\to s_2}(\mathfrak{I}_j)/2\big)\exp\left\{\boldsymbol{\beta}_{s_1\to s_2}^{\mathsf{T}}\boldsymbol{\eta}_{i,s_1\to s_2}(\mathfrak{I}_j)\right\}\right]\\
\widetilde{epl} &= \sum_{(s_1,s_2)\in\mathbb{E}}\sum_{j=1}^{N_t}\sum_{i=1}^{N}\Big[\widetilde{\mathrm{E}}_i\big[g_{i,s_1}(\mathfrak{I}_j)\delta_{i,s_1\to s_2}(\mathfrak{I}_j)\boldsymbol{\beta}_{s_1\to s_2}^{\mathsf{T}}\boldsymbol{\eta}_{i,s_1\to s_2}(\mathfrak{I}_j)\big]\\
&\quad - \widetilde{\mathrm{E}}_i[g_{i,s_1}(\mathfrak{I}_j)\delta_{i,s_1\to s_2}(\mathfrak{I}_j)]\log\{A_{s_1\to s_2}(\mathfrak{I}_j)\}\Big]\\
\frac{\partial \widetilde{epl}}{\partial\boldsymbol{\beta}_{s_1\to s_2}} &= \sum_{j=1}^{N_t}\sum_{i=1}^{N}\Big[\widetilde{\mathrm{E}}_i\big[g_{i,s_1}(\mathfrak{I}_j)\delta_{i,s_1\to s_2}(\mathfrak{I}_j)\boldsymbol{\eta}_{i,s_1\to s_2}(\mathfrak{I}_j)\big]\\
&\quad - \widetilde{\mathrm{E}}_i[g_{i,s_1}(\mathfrak{I}_j)\delta_{i,s_1\to s_2}(\mathfrak{I}_j)]\frac{1}{A_{s_1\to s_2}(\mathfrak{I}_j)}\frac{\partial A_{s_1\to s_2}(\mathfrak{I}_j)}{\partial\boldsymbol{\beta}_{s_1\to s_2}}\Big]\\
\frac{\partial^2 \widetilde{epl}}{(\partial\boldsymbol{\beta}_{s_1\to s_2})^2} &= \sum_{j=1}^{N_t}\sum_{i=1}^{N}\widetilde{\mathrm{E}}_i[g_{i,s_1}(\mathfrak{I}_j)\delta_{i,s_1\to s_2}(\mathfrak{I}_j)]\Big[\frac{1}{A_{s_1\to s_2}(\mathfrak{I}_j)^2}\left(\frac{\partial A_{s_1\to s_2}(\mathfrak{I}_j)}{\partial\boldsymbol{\beta}_{s_1\to s_2}}\right)^{\otimes 2}\\
&\quad - \frac{1}{A_{s_1\to s_2}(\mathfrak{I}_j)}\frac{\partial^2 A_{s_1\to s_2}(\mathfrak{I}_j)}{(\partial\boldsymbol{\beta}_{s_1\to s_2})^2}\Big].
\end{aligned}
$$

In the above procedure, we will need to evaluate several conditional expectations $\widetilde{\mathrm{E}}[\cdot]$ that take the two forms $\widetilde{\mathrm{E}}_i[\psi(\mathbf{b}_i)]$ and $\widetilde{\mathrm{E}}_i[\phi(\mathcal{A}_i)\psi(\mathbf{b}_i)]$, where ϕ and ψ are certain functions of $\mathcal{S}_i$ and $\mathbf{b}_i$. We can rewrite the conditional expectations in terms of $\mathrm{E}_{\mathbf{b}_i}[\cdot|\mathcal{Y}_i]$ and $\mathrm{E}_{\mathcal{A}_i}[\cdot|\mathcal{S}_i,\mathbf{b}_i]$

$$
\begin{aligned}
\widetilde{\mathrm{E}}_i[\psi(\mathbf{b}_i)] &= \frac{\int \psi(\mathbf{b}_i)f(\mathcal{S}_i|\mathbf{b}_i)f(\mathbf{b}_i|\mathcal{Y}_i)\,d\mathbf{b}_i}{\int f(\mathcal{S}_i|\mathbf{b}_i)f(\mathbf{b}_i|\mathcal{Y}_i)\,d\mathbf{b}_i} = \frac{\mathrm{E}_{\mathbf{b}_i}[\psi(\mathbf{b}_i)f(\mathcal{S}_i|\mathbf{b}_i)|\mathcal{Y}_i]}{\mathrm{E}_{\mathbf{b}_i}[f(\mathcal{S}_i|\mathbf{b}_i)|\mathcal{Y}_i]}\\
\widetilde{\mathrm{E}}_i[\phi(\mathcal{A}_i)\psi(\mathbf{b}_i)] &= \frac{\int\int \phi(\mathcal{A}_i)f(\mathcal{A}_i,\mathcal{S}_i|\mathbf{b}_i)\psi(\mathbf{b}_i)f(\mathbf{b}_i|\mathcal{Y}_i)\,d\mathcal{A}_i d\mathbf{b}_i}{\int\int f(\mathcal{A}_i,\mathcal{S}_i|\mathbf{b}_i)f(\mathbf{b}_i|\mathcal{Y}_i)\,d\mathcal{A}_i d\mathbf{b}_i}\\
&= \frac{\mathrm{E}_{\mathbf{b}_i}\left[\,\mathrm{E}_{\mathcal{A}_i}[\phi(\mathcal{A}_i)|\mathcal{S}_i,\mathbf{b}_i]\psi(\mathbf{b}_i)f(\mathcal{S}_i|\mathbf{b}_i)\big|\mathcal{Y}_i\right]}{\mathrm{E}_{\mathbf{b}_i}[f(\mathcal{S}_i|\mathbf{b}_i)|\mathcal{Y}_i]}.
\end{aligned}
$$

$\mathrm{E}_{\mathcal{A}_i}[\cdot|\mathcal{S}_i,\mathbf{b}_i]$ can be evaluated correspondingly as linear combinations of the following two terms

$$
\begin{aligned}
\mathrm{E}_{\mathcal{A}_i}[g_{i,s_1}(\mathfrak{I}_j)\delta_{i,s_1\to s_2}(\mathfrak{I}_j)|\mathcal{S}_i,\mathbf{b}_i] &= \mathrm{P}_{\mathcal{A}_i}\left(g_{i,s_1}(\mathfrak{I}_j)\delta_{i,s_1\to s_2}(\mathfrak{I}_j)=1\big|\mathcal{S}_i,\mathbf{b}_i\right) = \kappa_{i,j,s_1,s_2}^{g\delta}(\mathbf{b}_i)/\kappa_i^0(\mathbf{b}_i)\\
\mathrm{E}_{\mathcal{A}_i}[g_{i,s_1}(\mathfrak{I}_j)|\mathcal{S}_i,\mathbf{b}_i] &= \mathrm{P}_{\mathcal{A}_i}\left(g_{i,s_1}(\mathfrak{I}_j)=1\big|\mathcal{S}_i,\mathbf{b}_i\right) = \kappa_{i,j,s_1}^{g}(\mathbf{b}_i)/\kappa_i^0(\mathbf{b}_i)
\end{aligned}
$$

where, for each j such that $\mathfrak{I}_j \subset (T_{i,J-1}, T_{iJ}]$,

$$\kappa^{g\delta}_{i,j,s_1,s_2}(\mathbf{b}_i) = [\![\mathbf{1}]\!]^{\mathsf{T}}_{S_{i0}} \left[\prod_{j=1}^{J-1} [\![\mathbf{P}_{i,(T_{i,j-1},T_{ij}]}(\mathbf{b}_i)]\!]_{S_{i,j-1},S_{ij}} \right]$$
$$\times [\![\mathbf{P}_{i,(T_{i,J-1},\tau_{j-1}]}(\mathbf{b}_i)]\!]_{S_{i,J-1},s_1} [\![\mathbf{P}_{i,(\tau_{j-1},\tau_j]}(\mathbf{b}_i)]\!]_{s_1,s_2} [\![\mathbf{P}_{i,(\tau_j,T_{iJ}]}(\mathbf{b}_i)]\!]_{s_2,S_{i,J}}$$
$$\times \left[\prod_{j=J+1}^{n_i} [\![\mathbf{P}_{i,(T_{i,j-1},T_{ij}]}(\mathbf{b}_i)]\!]_{S_{i,j-1},S_{ij}} \right] [\![\mathbf{1}]\!]_{S_{in_i}}$$

$$\kappa^{g}_{i,j,s_1}(\mathbf{b}_i) = [\![\mathbf{1}]\!]^{\mathsf{T}}_{S_{i0}} \left[\prod_{j=1}^{J-1} [\![\mathbf{P}_{i,(T_{i,j-1},T_{ij}]}(\mathbf{b}_i)]\!]_{S_{i,j-1},S_{ij}} \right]$$
$$\times [\![\mathbf{P}_{i,(T_{i,J-1},\tau_{j-1}]}(\mathbf{b}_i)]\!]_{S_{i,J-1},s_1} [\![\mathbf{P}_{i,(\tau_{j-1},T_{iJ}]}(\mathbf{b}_i)]\!]_{s_1,S_{iJ}}$$
$$\times \left[\prod_{j=J+1}^{n_i} [\![\mathbf{P}_{i,(T_{i,j-1},T_{ij}]}(\mathbf{b}_i)]\!]_{S_{i,j-1},S_{ij}} \right] [\![\mathbf{1}]\!]_{S_{in_i}}$$

$$\kappa^{0}_{i}(\mathbf{b}_i) = [\![\mathbf{1}]\!]^{\mathsf{T}}_{S_{i0}} \left[\prod_{j=1}^{n_i} [\![\mathbf{P}_{i,(T_{i,j-1},T_{ij}]}(\mathbf{b}_i)]\!]_{S_{i,j-1},S_{ij}} \right] [\![\mathbf{1}]\!]_{S_{in_i}},$$

Note that

$$\log f(\mathbf{b}_i|\mathcal{Y}_i) = \log f(\mathcal{Y}_i|\mathbf{b}_i) + \log f(\mathbf{b}_i) - \log f(\mathcal{Y}_i).$$

Since both $\log f(\mathcal{Y}_i|\mathbf{b}_i)$ and $\log f(\mathbf{b}_i)$ are quadratic forms of $\mathbf{b}_i$, $f(\mathbf{b}_i|\mathcal{Y}_i)$ is also a probability density function for a multivariate normal distribution. $\mathrm{E}_{\mathbf{b}_i}[\cdot|\mathcal{Y}_i]$ can therefore be evaluated by Monte Carlo samples from a multivariate normal distribution with mean $\mathrm{E}[\mathbf{b}_i|\mathcal{Y}_i]$ and variance $\mathrm{Var}(\mathbf{b}_i|\mathcal{Y}_i)$. By taking derivatives of $\log f(\mathbf{b}_i|\mathcal{Y}_i)$ with respect to $\mathbf{b}_i$, we can find the corresponding mean and variance

$$\mathrm{E}[\mathbf{b}_i|\mathcal{Y}_i] = \mathbf{c} + \left[\boldsymbol{\Sigma}_a^{-1} + \sum_{k=1}^{N_y} (\mathbf{X}_{ik}\mathbf{I}_k)^{\mathsf{T}} (\mathbf{X}_{ik}\mathbf{I}_k)/\sigma_k^2 \right]^{-1} \sum_{k=1}^{N_y} (\mathbf{X}_{ik}\mathbf{I}_k)^{\mathsf{T}} (\mathbf{y}_{ik} - \mathbf{X}_{ik}\mathbf{c}_k)/\sigma_k^2$$

$$\mathrm{Var}(\mathbf{b}_i|\mathcal{Y}_i) = \left[\boldsymbol{\Sigma}_a^{-1} + \sum_{k=1}^{N_y} (\mathbf{X}_{ik}\mathbf{I}_k)^{\mathsf{T}} (\mathbf{X}_{ik}\mathbf{I}_k)/\sigma_k^2 \right]^{-1}$$

where $\mathbf{I}_k$ is a row subview of the identity matrix $\mathbf{I}_{N_a \times N_a}$ corresponding to the dimensions of $\mathbf{b}_{ik}$ in $\mathbf{b}_i = \left(\mathbf{b}_{i1}^{\mathsf{T}}, \ldots, \mathbf{b}_{iN_y}^{\mathsf{T}}\right)^{\mathsf{T}}$, such that $\mathbf{b}_{ik} = \mathbf{I}_k \mathbf{b}_i$. Let $\{\breve{\mathbf{b}}_i^{(j)}\}_{j=1}^{N_{\mathrm{mc}}}$ be N_{mc} Monte Carlo samples (or Gaussian quadrature points, or quasi-random sequences) with weights $\{\breve{w}_i^{(j)}\}_{j=1}^{N_{\mathrm{mc}}}$ from a multivariate normal distribution with mean $\mathrm{E}[\mathbf{b}_i|\mathcal{Y}_i]$ and variance $\mathrm{Var}(\mathbf{b}_i|\mathcal{Y}_i)$ (c.f., Wulfsohn and Tsiatis 1997;

Rizopoulos 2012), then we can use the following approximation

$$\widetilde{\mathrm{E}}_i[\psi(\mathbf{b}_i)] = \frac{\sum_{j=1}^{N_{\mathrm{mc}}} \breve{w}_i^{(j)} \psi(\breve{\mathbf{b}}_i^{(j)}) f(\mathcal{S}_i|\breve{\mathbf{b}}_i^{(j)})}{\sum_{j=1}^{N_{\mathrm{mc}}} \breve{w}_i^{(j)} f(\mathcal{S}_i|\breve{\mathbf{b}}_i^{(j)})}$$

$$\widetilde{\mathrm{E}}_i[\phi(\mathcal{A}_i)\psi(\mathbf{b}_i)] = \frac{\sum_{j=1}^{N_{\mathrm{mc}}} \breve{w}_i^{(j)} \,\mathrm{E}_{\mathcal{A}_i}[\phi(\mathcal{A}_i)|\mathcal{S}_i, \breve{\mathbf{b}}_i^{(j)}] \psi(\breve{\mathbf{b}}_i^{(j)}) f(\mathcal{S}_i|\breve{\mathbf{b}}_i^{(j)})}{\sum_{j=1}^{N_{\mathrm{mc}}} \breve{w}_i^{(j)} f(\mathcal{S}_i|\breve{\mathbf{b}}_i^{(j)})}.$$

Typically, $\breve{w}_i^{(j)} = 1/N_{\mathrm{mc}}$ when Monte Carlo samples or quasi-random sequences are used. When using multivariate Gaussian quadrature, we refer readers to Jäckel (2005) for determining the sample points and weights.

In this work, when the dimension of the numerical integration was low enough for the number of quadrature points to be computationally manageable, Gaussian quadrature was used. When the dimension of the numerical integration was high and the number of required quadrature points exceeded 1000, we used quasi-random sequences.

S2.2 Model convergence and diagnosis of non-convergence

In this manuscript, we assess the convergence of the algorithm by monitoring the change in $\widehat{\boldsymbol{\beta}}_{s_1\to s_2}$. Let $\widehat{\boldsymbol{\beta}}_{s_1\to s_2}^{(g)}$ and $\widehat{\boldsymbol{\beta}}_{s_1\to s_2}^{(g+1)}$ denote the estimated coefficients at consecutive iterations. Convergence was declared when $\max_{(s_1,s_2)\in\mathbb{E}} \left\|\widehat{\boldsymbol{\beta}}_{s_1\to s_2}^{(g+1)} - \widehat{\boldsymbol{\beta}}_{s_1\to s_2}^{(g)}\right\|_\infty < 0.0001$. To avoid premature convergence due to differences in the scales/ranges of covariates, we standardized covariates to have means of 0 and standard deviations of 1 when their scales/ranges are very different. Reported estimates of $\widehat{\boldsymbol{\beta}}_{s_1\to s_2}$ were transformed to match the original scale.

Numerical singularities may occasionally arise and lead to non-convergence. Our simulations indicated that most cases were associated with negative values in the diagonal elements of the transition probability matrices

$$[\![\mathbf{P}_{i,\mathfrak{I}_j}(\mathbf{b}_i;\boldsymbol{\Theta}_{\beta,h})]\!]_{\{s_1\},\{s_2\}} = \begin{cases} \mathrm{P}\left(\delta_{i,s_1\to s_2}(\mathfrak{I}_j) = 1 \middle| g_{i,s_1}(\mathfrak{I}_j) = 1\right) & s_1 \neq s_2 \\ 1 - \sum_{s_2:s_2\neq s_1} \mathrm{P}\left(\delta_{i,s_1\to s_2}(\mathfrak{I}_j) = 1 \middle| g_{i,s_1}(\mathfrak{I}_j) = 1\right) & s_1 = s_2 \end{cases}. \tag{S5}$$

We note that with our approximation,

$$\mathrm{P}\left(\delta_{i,s_1\to s_2}(\mathfrak{I}_j) = 1 \middle| g_{i,s_1}(\mathfrak{I}_j) = 1\right) \approx 1 - \exp\left\{ - h_{0,s_1\to s_2}(\mathfrak{I}_j) \exp\left[\boldsymbol{\beta}_{s_1\to s_2}^{\mathsf{T}} \boldsymbol{\eta}_{i,s_1\to s_2}(\mathfrak{I}_j)\right]\right\}.$$

Therefore $P\Big(s_i(\tau_j) = s_2 \Big| s_i(\tau_{j-1}) = s_1\Big)$ are not bounded from above and can be large when $h_{0,s_1\to s_2}(\mathfrak{I}_j)$ is large, and thus singularities may occur when $1-\sum_{s_2:s_2\neq s_1} \mathrm{P}\left(\delta_{i,s_1\to s_2}(\mathfrak{I}_j) = 1 \Big| g_{i,s_1}(\mathfrak{I}_j) = 1\right) < 0$. This singularity usually suggests that the number of intervals N_t is chosen too small, and can be resolved by partitioning $\mathfrak{I}_j$ into more subintervals or increasing the number of N_t. Here, we describe three complementary strategies for a systematic treatment of the problem.

S2.2.1 Technique 1. Filtering Monte Carlo draws leading to zero/negative likelihood

This serves as the first layer of safeguard in our algorithm. Although it was originally designed to handle cases where $h_{0,s_1\to s_2}(\mathfrak{I}_j)$ is close to zero, a closer inspection of our implementation shows that it also mitigates issues arising from extreme draws of $\mathbf{b}_i$ that produce ill-posed transition matrices.

By construction, all entries of the transition matrix $\mathbf{P}_{i,\mathfrak{I}_j}(\mathbf{b}_i)$ should lie in $[0, 1]$. Thus, violations typically manifest as negative diagonal entries or zero/negative off-diagonal entries, indicating that given the sampled $\mathbf{b}_i$, it is unlikely to observe individual i remained in the corresponding state in the interval $\mathfrak{I}_j$. For each Monte Carlo draw of $\mathbf{b}$ (from the distribution of $\mathbf{b}_i \mid \mathcal{Y}_i$), we will check the likelihood contribution

$$f(\mathcal{S}_i|\mathbf{b}_i) = [\![\mathbf{1}]\!]^\intercal_{S_{i0}} \left[\prod_{j=1}^{n_i} [\![\mathbf{P}_{i,(T_{i,j-1},T_{ij}]}(\mathbf{b}_i)]\!]_{S_{i,j-1},S_{ij}}\right] [\![\mathbf{1}]\!]_{S_{in_i}}.$$

When invalid entries are present, this quantity may become negative or zero, indicating that the observed transitions are not likely with the given draw. In practice, we can conservatively truncate negative entries in $\mathbf{P}_{i,\mathfrak{I}_j}(\mathbf{b}_i)$ to 0 and compute $f(\mathcal{S}_i|\mathbf{b}_i)$. If the resulting value is 0, it implies that the observed data given $\breve{\mathbf{b}}_i$ is unlikely, and $f(\mathcal{S}_i|\mathbf{b}_i)$ should be treated as 0. Under the Monte Carlo E-step,

$$\widetilde{\mathrm{E}}_i[\psi(\mathbf{b}_i)] = \frac{\sum_{j=1}^{N_{\mathrm{mc}}} \breve{w}_i^{(j)} \psi(\breve{\mathbf{b}}_i^{(j)}) f(\mathcal{S}_i|\breve{\mathbf{b}}_i^{(j)})}{\sum_{j=1}^{N_{\mathrm{mc}}} \breve{w}_i^{(j)} f(\mathcal{S}_i|\breve{\mathbf{b}}_i^{(j)})}$$

$$\widetilde{\mathrm{E}}_i[\phi(\mathcal{A}_i)\psi(\mathbf{b}_i)] = \frac{\sum_{j=1}^{N_{\mathrm{mc}}} \breve{w}_i^{(j)} \mathrm{E}_{\mathcal{A}_i}[\phi(\mathcal{A}_i)|\mathcal{S}_i, \breve{\mathbf{b}}_i^{(j)}] \psi(\breve{\mathbf{b}}_i^{(j)}) f(\mathcal{S}_i|\breve{\mathbf{b}}_i^{(j)})}{\sum_{j=1}^{N_{\mathrm{mc}}} \breve{w}_i^{(j)} f(\mathcal{S}_i|\breve{\mathbf{b}}_i^{(j)})},$$

so $f(\mathcal{S}_i|\mathbf{b}_i) = 0$ implies that this draw of $\mathbf{b}_i$ will not contribute to the weighted average, and equivalently we can exclude this draw from the Monte Carlo E-step.

This approach avoids imposing artificial corrections on the transition matrices and relies only on validity checks of computed quantities. However, it may be insufficient when invalid draws occur frequently, in which case additional measures are needed.

S2.2.2 Technique 2. Refinement of the time discretization

In both simulations and real data analyses, when convergence fails under a given partition $\mathfrak{I}_j$, we refine the discretization by increasing the number of intervals. Specifically, we replace N_t with $2N_t$ by splitting each interval $\mathfrak{I}_j$ into two subintervals and refit the model. If numerical issues persist, we further increase the resolution (e.g., to $3N_t$).

While effective in practice, this approach has two drawbacks: it requires repeated model fitting and increases the number of nuisance parameters $h_{0,s_1\to s_2}(\mathfrak{I}_j)$, thereby raising computational cost.

S2.2.3 Technique 3. On-the-fly interval splitting within the E-step

To more fully address the reviewer's concern, we propose an additional technique that systematically regularizes ill-posed transition matrices without requiring model refitting. Suppose that for a given draw $\breve{\mathbf{b}}_i^{(j)}$, the matrix $\mathbf{P}_{i,\mathfrak{I}_j}(\breve{\mathbf{b}}_i^{(j)})$ is ill-posed. This typically occurs when the cumulative hazard in the interval $\mathfrak{I}_j$ $h_{i,s_1\to s_2}(\mathfrak{I}_j) = h_{0,s_1\to s_2}(\mathfrak{I}_j)\exp\{\boldsymbol{\beta}_{s_1\to s_2}^\top \boldsymbol{\eta}_{i,s_1\to s_2}(\mathfrak{I}_j)\}$ is not sufficiently small. Motivated by Technique 2, we locally refine the interval $\mathfrak{I}_j$ by subdividing it into Q equal auxiliary subintervals $\mathfrak{I}_j^1,\dots,\mathfrak{I}_j^Q$ assuming constant hazard within $\mathfrak{I}_j$. For illustrative purposes, we denote the corresponding hazard function in these auxiliary intervals by $h_{i,s_1\to s_2}(\mathfrak{I}_j^q) = h_{i,s_1\to s_2}(\mathfrak{I}_j)/Q$, and the event processes by $\delta_{i,s_1\to s_2}(\mathfrak{I}_j^q)$. By properties of the complementary log-log link,

$$\exp\{-h_{i,s_1\to s_2}(\mathfrak{I}_j)\} = \left[\exp\{-h_{i,s_1\to s_2}(\mathfrak{I}_j)/Q\}\right]^Q = \prod_{q=1}^{Q}\exp\{-h_{i,s_1\to s_2}(\mathfrak{I}_j^q)\}$$

which has a clear interpretation that the probability of $\delta_{i,s_1\to s_2}(\mathfrak{I}_j)=1$ is exactly the probability of $\max_{q=1,\dots,Q}\delta_{i,s_1\to s_2}(\mathfrak{I}_j^q)=1$, and therefore, implies that the representation is fully consistent with our model formulation. More importantly, this construction ensures that the complete likelihood

can still be written in terms of $\delta_{i,s_1\to s_2}(\mathfrak{I}_j)$ as follows

$$\begin{aligned}&\log f(\mathcal{A}_i,\mathcal{S}_i|\mathbf{b}_i;\boldsymbol{\Theta}_{\beta,h})=\log f(\mathcal{A}_i|\mathbf{b}_i;\boldsymbol{\Theta}_{\beta,h})+\log f(\mathcal{S}_i|\mathcal{A}_i,\mathbf{b}_i)=\\&\sum_{j=1}^{N_t}\sum_{(s_1,s_2)\in\mathbb{E}}g_{i,s_1}(\mathfrak{I}_j)\Big[\delta_{i,s_1\to s_2}(\mathfrak{I}_j)\log\Big\{\exp\left\{h_{0,s_1\to s_2}(\mathfrak{I}_j)\exp\left(\boldsymbol{\beta}_{s_1\to s_2}^{\mathsf{T}}\boldsymbol{\eta}_{i,s_1\to s_2}(\mathfrak{I}_j)\right)\right\}-1\Big\}\\&-h_{0,s_1\to s_2}(\mathfrak{I}_j)\exp\left\{\boldsymbol{\beta}_{s_1\to s_2}^{\mathsf{T}}\boldsymbol{\eta}_{i,s_1\to s_2}(\mathfrak{I}_j)\right\}\Big]+\log f(\mathcal{S}_i|\mathcal{A}_i,\mathbf{b}_i)\end{aligned}$$

and the first order approximation $\log f^{(1)}(\mathcal{A}_i,\mathcal{S}_i|\mathbf{b}_i;\boldsymbol{\Theta}_{\beta,h})$ can still hold true. As a result, the M-step of the EM algorithm can still proceed without modification. The adjustment is only implemented in the E-step. More specifically, we replace the transition probability matrix $\mathbf{P}_{i,\mathfrak{I}_j}(\breve{\mathbf{b}}_i^{(j)})$ by the product of transition probability matrices over the subintervals $\prod_{q=1}^{Q}\mathbf{P}_{i,\mathfrak{I}_j^q}(\breve{\mathbf{b}}_i^{(j)})$, where

$$[\![\mathbf{P}_{i,\mathfrak{I}_j^q}(\breve{\mathbf{b}}_i^{(j)})]\!]_{\{s_1\},\{s_2\}}=\begin{cases}1-\exp\{-h_{i,s_1\to s_2}(\mathfrak{I}_j^q)\}=1-\exp\{-h_{i,s_1\to s_2}(\mathfrak{I}_j)/Q\} & s_1\neq s_2\\ 1-\sum_{s_2:s_2\neq s_1}[\![\mathbf{P}_{i,\mathfrak{I}_j^q}(\breve{\mathbf{b}}_i^{(j)})]\!]_{\{s_1\},\{s_2\}} & s_1=s_2\end{cases},$$

The number of subintervals Q is chosen to be the smallest positive integer that makes the matrix $\mathbf{P}_{i,\mathfrak{I}_j^q}(\breve{\mathbf{b}}_i^{(j)})$ become a valid transition matrix.

This approach ensures numerical stability of the E-step while preserving the original likelihood structure, and avoids any ad hoc truncation or modification of transition probabilities.

S3 Additional Details on Dynamic Predictions

Dynamic predictions are calculated based on the probability of occupying state s_1 given the observed data. The formula for dynamic predictions has been provided in the manuscript as follows

$$p_{i,s_1}(t_{\mathrm{L}},t_{\mathrm{LP}})=\mathrm{P}(g_{i,s_1}(\mathfrak{I}_{\mathrm{LP}+1})=1|\mathcal{S}_i,\mathcal{Y}_i)=\frac{\mathrm{E}_{\mathbf{b}_i}\left[\mathrm{P}_{\mathcal{A}_i}(g_{i,s_1}(\mathfrak{I}_{\mathrm{LP}+1})=1|\mathcal{S}_i,\mathbf{b}_i)f(\mathcal{S}_i|\mathbf{b}_i)|\mathcal{Y}_i\right]}{\mathrm{E}_{\mathbf{b}_i}[f(\mathcal{S}_i|\mathbf{b}_i)|\mathcal{Y}_i]}.$$

We note that the above quantity can also be written as $\widetilde{\mathrm{E}}_i[g_{i,s_1}(\mathfrak{I}_{\mathrm{LP}+1})]$, so the technique used in the E-step of the EM algorithm can be exactly applied here to calculate dynamic predictions.

In the evaluation of dynamic predictions, when the testing dataset is observed without interval censoring, the quantities $\widehat{\mathrm{TPR}}_{s_e}(\rho,t_{\mathrm{L}},t_{\mathrm{LP}})$, $\widehat{\mathrm{FPR}}_{s_e}(\rho,t_{\mathrm{L}},t_{\mathrm{LP}})$, $\widehat{\mathrm{AUC}}_{s_e}(t_{\mathrm{L}},t_{\mathrm{LP}})$, $\widehat{\mathrm{BS}}_{s_e}(t_{\mathrm{L}},t_{\mathrm{LP}})$, and $\widehat{\mathrm{BS}}_{\mathbb{V}}(t_{\mathrm{L}},t_{\mathrm{LP}})$ can all be directly computed based on $\widehat{p}_{i,s_1}(t_{\mathrm{L}},t_{\mathrm{LP}})$. However, in the computation

of $\widetilde{\mathrm{TPR}}_{s_e}(\rho, t_{\mathrm{L}}, t_{\mathrm{LP}})$, $\widetilde{\mathrm{FPR}}_{s_e}(\rho, t_{\mathrm{L}}, t_{\mathrm{LP}})$, $\widetilde{\mathrm{AUC}}_{s_e}(t_{\mathrm{L}}, t_{\mathrm{LP}})$, $\widetilde{\mathrm{BS}}_{s_e}(t_{\mathrm{L}}, t_{\mathrm{LP}})$, and $\widetilde{\mathrm{BS}}_{\mathbb{V}}(t_{\mathrm{L}}, t_{\mathrm{LP}})$, we need to additionally compute the conditional joint probabilities $\widehat{\mathrm{P}}\big(s_i(t_{\mathrm{L}}) \neq s_e, s_i(t_{\mathrm{LP}}) \neq s_e \big| \mathcal{S}_i^*, \mathcal{Y}_i^*\big)$ and $\widehat{\mathrm{P}}\big(s_i(t_{\mathrm{L}}) \neq s_e, s_i(t_{\mathrm{LP}}) = s_e \big| \mathcal{S}_i^*, \mathcal{Y}_i^*\big)$. We can still apply the techniques used in the E-step to compute these quantities by noting that

$$\widehat{\mathrm{P}}\big(s_i(t_{\mathrm{L}}) \neq s_e, s_i(t_{\mathrm{LP}}) \neq s_e \big| \mathcal{S}_i^*, \mathcal{Y}_i^*\big) = \sum_{s_1 \neq s_e} \sum_{s_2 \neq s_e} \widetilde{\mathrm{E}}_i[g_{i,s_1}(\mathfrak{I}_{\mathrm{L}+1}) g_{i,s_2}(\mathfrak{I}_{\mathrm{LP}+1})]$$
$$\widehat{\mathrm{P}}\big(s_i(t_{\mathrm{L}}) \neq s_e, s_i(t_{\mathrm{LP}}) = s_e \big| \mathcal{S}_i^*, \mathcal{Y}_i^*\big) = \sum_{s_1 \neq s_e} \widetilde{\mathrm{E}}_i[g_{i,s_1}(\mathfrak{I}_{\mathrm{L}+1}) g_{i,s_e}(\mathfrak{I}_{\mathrm{LP}+1})].$$

S4 Considerations on Discrete Approximation

S4.1 General consideration

In this section, we will present some considerations on discrete approximation. The main consideration is to translate the observation times of state transitions $T_{i0} < T_{i1} < \ldots < T_{in_i}$ into prespecified subintervals, $\mathfrak{I}_1 = (\tau_0, \tau_1], \mathfrak{I}_2 = (\tau_1, \tau_2], \ldots, \mathfrak{I}_{N_t} = (\tau_{N_t-1}, \tau_{N_t}]$, and balance estimation accuracy and computational efficiency. In Gu et al. (2024), a method without approximation was adopted, where the time sequence $\tau_0 < \ldots < \tau_N$ is selected to include all unique observation times. However, when the number of subintervals increases with the number of unique observations, the method will become computationally intensive. A simple discrete approximation to reduce the number of unique observation times is to choose τ_g as integer multiples of a small time unit ω (i.e., let $\tau_g = g\omega$), and round T_{ij} to the nearest values of τ_g. Consequently, the number of subintervals will be controlled by the size of ω, and a balance between computational efficiency and estimation accuracy can be controlled by a proper selection of ω.

Also, we usually need to check if the discrete approximation scheme is admissible. For j_1 and j_2 such that $1 \leq j_1 < j_2 \leq n_i$, we say S_{i,j_1} and S_{i,j_2} are attainable within n transitions if there exists a state $s_1 \in S_{i,j_1}$ a state $s_2 \in S_{i,j_2}$, and a directed path on the graph $(\mathbb{V}, \mathbb{E})$ connecting s_1 and s_2, such that the length of the directed path is less than n. Let $n_{(T_{ij_1}, T_{ij_2}]}$ be the number of subintervals in $(T_{ij_1}, T_{ij_2}]$, (i.e., $n_{(T_{ij_1}, T_{ij_2}]} = |\{\mathfrak{I}_g : \mathfrak{I}_g \subset (T_{ij_1}, T_{ij_2}]\}|$), the discrete approximation is admissible if any S_{i,j_1} and S_{i,j_2} are attainable within $n_{(T_{ij_1}, T_{ij_2}]}$.

For clarity and simplicity, in our current manuscript, we used equally spaced time intervals $\tau_j = j\omega$, where ω is the length of the intervals. The observed transition times are rounded to the nearest τ_j.

S4.2 Additional simulation studies to evaluate the impact of approximation on model estimation

As noted previously, the implementation of the proposed model involves the selection of the number of subintervals $\mathfrak{I}_1 = (\tau_0, \tau_1], \mathfrak{I}_2 = (\tau_1, \tau_2], \ldots, \mathfrak{I}_{N_t} = (\tau_{N_t-1}, \tau_{N_t}]$, which should be chosen to balance estimation efficiency and computational burden. In our current work, we used equally spaced intervals $\tau_j = j\omega$, and the number of intervals N_t is the only design parameter that needs to be determined. In this subsection, we will use a simple multistate model as presented in Figure S1 to investigate how the choice of N_t will impact our results. We performed 500 simulations with sample sizes $m = 1000$, 2000, and 3000. The design time interval was partitioned into 10, 25, 50, 75, 100, 200, 500, or 1000 subintervals. $h_{0,s_1 \to s_2}(t) = 2$, $h_{0,s_1 \to s_3}(t) = 0.5$ and $h_{0,s_2 \to s_3}(t) = 2$. For simplicity, we only included one non-time-varying risk factor $\mathbf{z}_i = (z_{i1})$ following standard normal distributions. The biases and overall mean-squared errors (MSE) for the estimates of coefficients are presented in Table S1. From this table, we can see that the biases and overall MSE tend to be large when N_t is unreasonably small. In this example, the overall MSEs are generally comparable when $N_t \geq 75$. When the sample size is sufficiently large ($m = 3000$), it is quite clear that a larger N_t gives better estimation. However, when $m = 1000$, we notice that a large $N_t = 1000$ may sometimes give a larger overall MSE compared to other choices of smaller N_t. We hypothesize that this is a coincidence where small sample size biases happen to cancel out the biases in the discrete approximation. In our simulation studies, N_t ranged from 100 to 200. For practical applications, we suggest choosing N_t to be of the same order as the sample size and caution against using excessively small values.

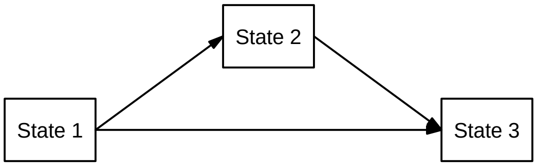

Figure S1: A simple three-state model for evaluating the impact of discrete approximation.

Table S1: Estimated regression coefficients in the three-state model by varying the sample size m and the number of intervals N_t.

m	N_t	$\beta_{1\to 2} = 0.3$	$\beta_{1\to 3} = 0.1$	$\beta_{2\to 3} = 0.5$	Overall MSE
1000	10	0.245, bias: -0.055, se: 0.002	0.274, bias: 0.174, se: 0.004	0.531, bias: 0.031, se: 0.003	0.0476, se: 0.0014
1000	25	0.272, bias: -0.028, se: 0.002	0.196, bias: 0.096, se: 0.004	0.502, bias: 0.002, se: 0.002	0.0209, se: 0.0008
1000	50	0.292, bias: -0.008, se: 0.002	0.136, bias: 0.036, se: 0.004	0.507, bias: 0.007, se: 0.002	0.0151, se: 0.0007
1000	75	0.297, bias: -0.003, se: 0.002	0.116, bias: 0.016, se: 0.004	0.508, bias: 0.008, se: 0.002	0.0146, se: 0.0007
1000	100	0.299, bias: -0.001, se: 0.002	0.109, bias: 0.009, se: 0.004	0.508, bias: 0.008, se: 0.002	0.0147, se: 0.0007
1000	200	0.302, bias: 0.002, se: 0.002	0.097, bias: -0.003, se: 0.005	0.509, bias: 0.009, se: 0.002	0.0150, se: 0.0008
1000	500	0.304, bias: 0.004, se: 0.002	0.087, bias: -0.013, se: 0.005	0.510, bias: 0.010, se: 0.002	0.0152, se: 0.0008
1000	1000	0.305, bias: 0.005, se: 0.002	0.082, bias: -0.018, se: 0.005	0.510, bias: 0.010, se: 0.002	0.0156, se: 0.0008
2000	10	0.243, bias: -0.057, se: 0.001	0.279, bias: 0.179, se: 0.003	0.535, bias: 0.035, se: 0.002	0.0429, se: 0.0010
2000	25	0.270, bias: -0.030, se: 0.001	0.202, bias: 0.102, se: 0.002	0.502, bias: 0.002, se: 0.002	0.0165, se: 0.0006
2000	50	0.288, bias: -0.012, se: 0.001	0.149, bias: 0.049, se: 0.003	0.505, bias: 0.005, se: 0.002	0.0092, se: 0.0004
2000	75	0.294, bias: -0.006, se: 0.001	0.131, bias: 0.031, se: 0.003	0.506, bias: 0.006, se: 0.002	0.0076, se: 0.0004
2000	100	0.296, bias: -0.004, se: 0.001	0.122, bias: 0.022, se: 0.003	0.506, bias: 0.006, se: 0.002	0.0073, se: 0.0003
2000	200	0.299, bias: -0.001, se: 0.001	0.109, bias: 0.009, se: 0.003	0.507, bias: 0.007, se: 0.002	0.0069, se: 0.0003
2000	500	0.301, bias: 0.001, se: 0.001	0.100, bias: 0.000, se: 0.003	0.507, bias: 0.007, se: 0.002	0.0068, se: 0.0003
2000	1000	0.302, bias: 0.002, se: 0.001	0.097, bias: -0.003, se: 0.003	0.507, bias: 0.007, se: 0.002	0.0070, se: 0.0003
3000	10	0.244, bias: -0.056, se: 0.001	0.276, bias: 0.176, se: 0.002	0.528, bias: 0.028, se: 0.002	0.0393, se: 0.0008
3000	25	0.270, bias: -0.030, se: 0.001	0.202, bias: 0.102, se: 0.002	0.499, bias: -0.001, se: 0.001	0.0148, se: 0.0005
3000	50	0.288, bias: -0.012, se: 0.001	0.149, bias: 0.049, se: 0.003	0.502, bias: 0.002, se: 0.001	0.0071, se: 0.0003
3000	75	0.294, bias: -0.006, se: 0.001	0.128, bias: 0.028, se: 0.003	0.503, bias: 0.003, se: 0.001	0.0056, se: 0.0003
3000	100	0.296, bias: -0.004, se: 0.001	0.121, bias: 0.021, se: 0.003	0.503, bias: 0.003, se: 0.001	0.0053, se: 0.0003
3000	200	0.299, bias: -0.001, se: 0.001	0.107, bias: 0.007, se: 0.003	0.504, bias: 0.004, se: 0.001	0.0049, se: 0.0002
3000	500	0.301, bias: 0.001, se: 0.001	0.099, bias: -0.001, se: 0.003	0.504, bias: 0.004, se: 0.001	0.0049, se: 0.0002
3000	1000	0.302, bias: 0.002, se: 0.001	0.095, bias: -0.005, se: 0.003	0.504, bias: 0.004, se: 0.001	0.0050, se: 0.0002

S5 Computational Considerations

S5.1 Simplifying expressions for $\kappa^{g\delta}_{i,j,s_1,s_2}(\mathbf{b}_i)$, $\kappa^{g}_{i,j,s_1}(\mathbf{b}_i)$, and $\kappa^{0}_{i}(\mathbf{b}_i)$

In this subsection, we will introduce a trick to simplify computations of the iterated product in $\kappa^{g\delta}_{i,j,s_1,s_2}(\mathbf{b}_i)$, $\kappa^{g}_{i,j,s_1}(\mathbf{b}_i)$, and $\kappa^{0}_{i}(\mathbf{b}_i)$. Suppose that for certain j_1 and j_2 ($0 \leq j_1 < j_2 \leq n_i$), $|S_{ij_1}| = |S_{ij_2}| = 1$. For $\mathfrak{I}_j \subset (T_{i,J-1}, T_{iJ}] \subset (T_{ij_1}, T_{ij_2}]$, we can notice that several terms in the iterated product can be canceled out when evaluating $\kappa^{g\delta}_{i,j,s_1,s_2}(\mathbf{b}_i)/\kappa^{0}_{i}(\mathbf{b}_i)$ $\kappa^{g}_{i,j,s_1}(\mathbf{b}_i)/\kappa^{0}_{i}(\mathbf{b}_i)$.

Therefore,

$$
\begin{aligned}
\kappa^{g\delta}_{i,j,s_1,s_2}(\mathbf{b}_i) &= [\![\mathbf{1}]\!]^{\intercal}_{S_{ij_1}} \left[\prod_{j=j_1}^{J-1} [\![\mathbf{P}_{i,(T_{i,j-1},T_{ij}]}(\mathbf{b}_i)]\!]_{S_{i,j-1},S_{ij}} \right] \\
&\quad \times [\![\mathbf{P}_{i,(T_{i,J-1},\tau_{j-1}]}(\mathbf{b}_i)]\!]_{S_{i,J-1},s_1} [\![\mathbf{P}_{i,(\tau_{j-1},\tau_j]}(\mathbf{b}_i)]\!]_{s_1,s_2} [\![\mathbf{P}_{i,(\tau_j,T_{iJ}]}(\mathbf{b}_i)]\!]_{s_2,S_{i,J}} \\
&\quad \times \left[\prod_{j=J+1}^{j_2} [\![\mathbf{P}_{i,(T_{i,j-1},T_{ij}]}(\mathbf{b}_i)]\!]_{S_{i,j-1},S_{ij}} \right] [\![\mathbf{1}]\!]_{S_{ij_2}} \\
\kappa^{g}_{i,j,s_1}(\mathbf{b}_i) &= [\![\mathbf{1}]\!]^{\intercal}_{S_{ij_1}} \left[\prod_{j=j_1}^{J-1} [\![\mathbf{P}_{i,(T_{i,j-1},T_{ij}]}(\mathbf{b}_i)]\!]_{S_{i,j-1},S_{ij}} \right] \\
&\quad \times [\![\mathbf{P}_{i,(T_{i,J-1},\tau_{j-1}]}(\mathbf{b}_i)]\!]_{S_{i,J-1},s_1} [\![\mathbf{P}_{i,(\tau_{j-1},T_{iJ}]}(\mathbf{b}_i)]\!]_{s_1,S_{iJ}} \\
&\quad \times \left[\prod_{j=J+1}^{j_2} [\![\mathbf{P}_{i,(T_{i,j-1},T_{ij}]}(\mathbf{b}_i)]\!]_{S_{i,j-1},S_{ij}} \right] [\![\mathbf{1}]\!]_{S_{ij_2}} \\
\kappa^{0}_{i}(\mathbf{b}_i) &= [\![\mathbf{1}]\!]^{\intercal}_{S_{ij_1}} \left[\prod_{j=j_1}^{j_2} [\![\mathbf{P}_{i,(T_{i,j-1},T_{ij}]}(\mathbf{b}_i)]\!]_{S_{i,j-1},S_{ij}} \right] [\![\mathbf{1}]\!]_{S_{ij_2}},
\end{aligned}
$$

Additionally, we note that based on the properties of the directed graph $(\mathbb{V}, \mathbb{E})$, it can also be inferred that $\kappa^{g\delta}_{i,j,s_1,s_2}(\mathbf{b}_i)$ and $\kappa^{g}_{i,j,s_1}(\mathbf{b}_i)$ shall be 0 for certain combinations of s_1 and s_2. When $(\mathbb{V}, \mathbb{E})$ is a directed graph with no loops, a naive case that can significantly reduce computations is to check for cases where $S_{ij_1} = S_{ij_2} = \{s'\}$. In this case, $\kappa^{g}_{i,j,s_1} = 0$ when $s_1 \neq s'$, and $\kappa^{g\delta}_{i,j,s_1,s_2}(\mathbf{b}_i) = 0$ when either s_1 or s_2 is not s'.

S5.2 A dynamic strategy to accelerate the estimation

The most computationally intensive step in the estimation procedure is the approximation of numerical integrations and expectations using Monte Carlo samples $\big(\breve{\mathbf{b}}_i^{(j)}, \breve{w}_i^{(j)}\big)_{j=1}^{N_{\mathrm{mc}}}$. In the computational program, we used a dynamic technique to accelerate the model estimation, especially in the initial iterations. Basically, we allow N_{mc} to be a variable that depends on the number of iterations, and increase it gradually toward convergence. When the number of parameters in the model is large, and N_{mc} is small, sometimes numerical singularities will occur as parameters are updated based on numerical integrations that are not fully converged. To alleviate computational singularity while maintaining computational efficiency, sometimes a smaller Newton-Raphson step in updating $\boldsymbol{\beta}_{s_1 \to s_2}$ can be helpful. In our computer program, we also allow users to specify a dynamic step size

parameter σ that increases in the interval $(0,1]$ as the number of iterations increases

$$\boldsymbol{\beta}_{s_1\to s_2} \leftarrow \boldsymbol{\beta}_{s_1\to s_2} - \sigma\left[\frac{\partial^2 \widetilde{epl}}{(\partial\boldsymbol{\beta}_{s_1\to s_2})^2}\right]^{-1}\left[\frac{\partial \widetilde{epl}}{\partial\boldsymbol{\beta}_{s_1\to s_2}}\right].$$

S6 Illustrating Examples

In this section, we will present several examples to illustrate the proposed method, especially for more intuitive interpretations of the observed data, data augmentation parameters, and design parameters in the multistate models.

S6.1 Illustrating Example 1

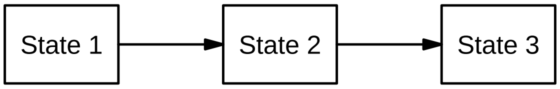

(a) The multistate model in Illustrating Example 1

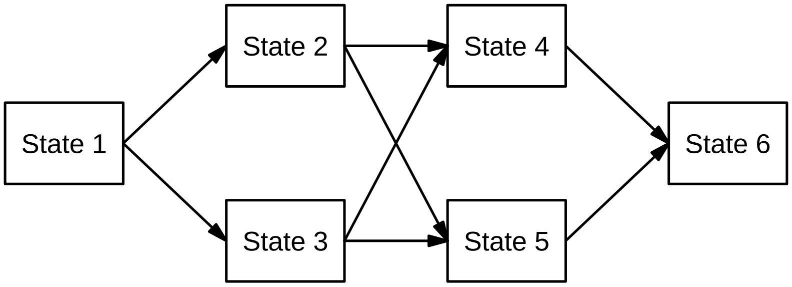

(b) The multistate model in Illustrating Example 2

We first consider a very simple 3-state model as illustrated in Figure S2a. Two individuals (Panels (a) and (b) in Figure S3) in the simulated dataset are presented along with the quantities of $\mathfrak{I}_j$, τ_j, $s_i(\mathfrak{I}_j)$, $g_{i,1}(\mathfrak{I}_j)$, $g_{i,2}(\mathfrak{I}_j)$, $g_{i,3}(\mathfrak{I}_j)$, $\delta_{i,1\to2}(\mathfrak{I}_j)$, $\delta_{i,2\to3}(\mathfrak{I}_j)$, S_{ij}, T_{ij}, $\widetilde{\mathrm{E}}[g_{i,1}(\mathfrak{I}_j)]$, $\widetilde{\mathrm{E}}[g_{i,2}(\mathfrak{I}_j)]$, $\widetilde{\mathrm{E}}[g_{i,3}(\mathfrak{I}_j)]$, $\widetilde{\mathrm{E}}[\delta_{i,1\to2}(\mathfrak{I}_j)]$, $\widetilde{\mathrm{E}}[\delta_{i,2\to3}(\mathfrak{I}_j)]$ from top to bottom, where the quantities of $\widetilde{\mathrm{E}}[g_{i,3}(\mathfrak{I}_j)]$, $\widetilde{\mathrm{E}}[\delta_{i,1\to2}(\mathfrak{I}_j)]$, $\widetilde{\mathrm{E}}[\delta_{i,2\to3}(\mathfrak{I}_j)]$ are the estimated quantities at convergence.

In this example, $N_t = 20$, $N_y = 1$, $N_z = 1$, $h_{0,1\to2}(t) = \exp(-t/2)$, $h_{0,2\to3} = \log(1+t)$, $\mathbf{z}_i$

(a)

Design intervals: I_j = $I_1, \ldots, I_{20}$; τ_j = $\tau_0, \tau_1, \ldots, \tau_{20}$

	I_1	I_2	I_3	I_4	I_5	I_6	I_7	I_8	I_9	I_{10}	I_{11}	I_{12}	I_{13}	I_{14}	I_{15}	I_{16}	I_{17}	I_{18}	I_{19}	I_{20}
**Unobserved Truth**																				
$s_i(I_j)$	1	1	1	1	1	1	1	2	2	2	2	2	2	2	2	2	3	3	3	3
$g_{i,1}(I_j)$	1	1	1	1	1	1	1	0	0	0	0	0	0	0	0	0	0	0	0	0
$g_{i,2}(I_j)$	0	0	0	0	0	0	0	1	1	1	1	1	1	1	1	1	0	0	0	0
$g_{i,3}(I_j)$	0	0	0	0	0	0	0	0	0	0	0	0	0	0	0	0	1	1	1	1
$\delta_{i,1\to2}(I_j)$	0	0	0	0	0	0	1	0	0	0	0	0	0	0	0	0	0	0	0	0
$\delta_{i,2\to3}(I_j)$	0	0	0	0	0	0	0	0	0	0	0	0	0	0	0	1	0	0	0	0
**Estimated Information**																				
$\tilde{E}_i[g_{i,1}(I_j)]$	1.00	1.00	0.77	0.56	0.37	0.23	0.05	0.00	0.00	0.00	0.00	0.00	0.00	0.00	0.00	0.00	0.00	0.00	0.00	0.00
$\tilde{E}_i[g_{i,2}(I_j)]$	0.00	0.00	0.23	0.44	0.63	0.77	0.95	1.00	1.00	1.00	1.00	1.00	0.84	0.72	0.69	0.63	0.43	0.30	0.20	0.13
$\tilde{E}_i[g_{i,3}(I_j)]$	0.00	0.00	−0.00	−0.00	0.00	0.00	0.00	0.00	0.00	0.00	0.00	0.00	0.16	0.28	0.31	0.37	0.57	0.70	0.80	0.87
$\tilde{E}_i[\delta_{i,1\to2}(I_j)]$	0.00	0.23	0.21	0.19	0.14	0.18	0.05	0.00	0.00	0.00	0.00	0.00	0.00	0.00	0.00	0.00	0.00	0.00	0.00	0.00
$\tilde{E}_i[\delta_{i,2\to3}(I_j)]$	0.00	0.00	0.00	0.00	0.00	0.00	0.00	0.00	0.00	0.00	0.00	0.16	0.12	0.03	0.06	0.21	0.12	0.10	0.07	0.13

Observed Data (S_{ij}, T_{ij}):
- T_{i0} (at τ_0): State 1, $S_{i0} = \{1\}$
- T_{i1} (at τ_1): State 1, $S_{i1} = \{1\}$
- T_{i2} (at τ_7): State 2, $S_{i2} = \{2\}$
- T_{i3} (at τ_{11}): State 2, $S_{i3} = \{2\}$
- T_{i4} (at τ_{17}): State 2 or 3, $S_{i4} = \{2,3\}$
- T_{i5} (at τ_{20}): State 3, $S_{i5} = \{3\}$

(b)

Design intervals: I_j = $I_1, \ldots, I_{20}$; τ_j = $\tau_0, \tau_1, \ldots, \tau_{20}$

	I_1	I_2	I_3	I_4	I_5	I_6	I_7	I_8	I_9	I_{10}	I_{11}	I_{12}	I_{13}	I_{14}	I_{15}	I_{16}	I_{17}	I_{18}	I_{19}	I_{20}
**Unobserved Truth**																				
$s_i(I_j)$	1	1	1	1	1	1	1	1	1	1	1	1	1	1	2	2	3	3	3	3
$g_{i,1}(I_j)$	1	1	1	1	1	1	1	1	1	1	1	1	1	1	0	0	0	0	0	0
$g_{i,2}(I_j)$	0	0	0	0	0	0	0	0	0	0	0	0	0	0	1	1	0	0	0	0
$g_{i,3}(I_j)$	0	0	0	0	0	0	0	0	0	0	0	0	0	0	0	0	1	1	1	1
$\delta_{i,1\to2}(I_j)$	0	0	0	0	0	0	0	0	0	0	0	0	0	1	0	0	0	0	0	0
$\delta_{i,2\to3}(I_j)$	0	0	0	0	0	0	0	0	0	0	0	0	0	0	0	1	0	0	0	0
**Estimated Information**																				
$\tilde{E}_i[g_{i,1}(I_j)]$	1.00	1.00	1.00	1.00	1.00	1.00	1.00	1.00	1.00	1.00	1.00	1.00	1.00	1.00	0.51	0.17	0.07	0.00	0.00	0.00
$\tilde{E}_i[g_{i,2}(I_j)]$	0.00	0.00	0.00	0.00	0.00	0.00	0.00	0.00	0.00	0.00	0.00	0.00	0.00	0.00	0.49	0.76	0.47	0.27	0.00	0.00
$\tilde{E}_i[g_{i,3}(I_j)]$	0.00	0.00	0.00	0.00	0.00	0.00	0.00	0.00	0.00	0.00	0.00	0.00	0.00	0.00	0.00	0.07	0.46	0.73	1.00	1.00
$\tilde{E}_i[\delta_{i,1\to2}(I_j)]$	0.00	0.00	0.00	0.00	0.00	0.00	0.00	0.00	0.00	0.00	0.00	0.00	0.00	0.49	0.34	0.11	0.07	0.00	0.00	0.00
$\tilde{E}_i[\delta_{i,2\to3}(I_j)]$	0.00	0.00	0.00	0.00	0.00	0.00	0.00	0.00	0.00	0.00	0.00	0.00	0.00	0.00	0.07	0.39	0.27	0.27	0.00	0.00

Observed Data (S_{ij}, T_{ij}):
- T_{i0} (at τ_0): State 1, $S_{i0} = \{1\}$
- T_{i1} (at τ_{13}): State 1, $S_{i1} = \{1\}$
- T_{i2} (at τ_{18}): State 3, $S_{i2} = \{3\}$

Figure S3: Parameters of two individuals in Illustrating Example 1.

follows an independent standard normal distribution, $B_{kl}(t)$ are linear basis functions, $\mathbf{c} = (0, 0)^\mathsf{T}$, and $\mathbf{\Sigma}_a = \begin{pmatrix} 1 & 0 \\ 0 & 0.2 \end{pmatrix}$.

The first individual transitions from State 1 to State 2 in $\mathfrak{I}_6$, and then from State 2 to State 3 in $\mathfrak{I}_{15}$. The true state transitions are only observed at certain timepoints. For example, at T_{i1}, we observe that the individual is in State 1, so S_{i1} is a set of single element $\{1\}$. At T_{i4}, we only know that the individual is in one of State 2 and State 3, so $S_{i4} = \{2, 3\}$ by our notations. The unobserved truth $g_{i,1}(\mathfrak{I}_j)$, $g_{i,2}(\mathfrak{I}_j)$, $g_{i,3}(\mathfrak{I}_j)$, $\delta_{i,1\to2}(\mathfrak{I}_j)$, $\delta_{i,2\to3}(\mathfrak{I}_j)$ are inferred in the estimation procedure by the quantities $\widetilde{\mathrm{E}}[g_{i,1}(\mathfrak{I}_j)]$, $\widetilde{\mathrm{E}}[g_{i,2}(\mathfrak{I}_j)]$, $\widetilde{\mathrm{E}}[g_{i,3}(\mathfrak{I}_j)]$, $\widetilde{\mathrm{E}}[\delta_{i,1\to2}(\mathfrak{I}_j)]$, $\widetilde{\mathrm{E}}[\delta_{i,2\to3}(\mathfrak{I}_j)]$. Based on the observed information, we know that the individual can only be in State 1 between T_{i0} and T_{i1} ($\widetilde{\mathrm{E}}[g_{i,1}(\mathfrak{I}_j)] = 1$), can only be in State 2 between T_{i2} to T_{i3} ($\widetilde{\mathrm{E}}[g_{i,2}(\mathfrak{I}_j)] = 1$). Between T_{i1} and T_{i2}, there are possibilities in either State 1 and State 2, so $\widetilde{\mathrm{E}}[g_{i,1}(\mathfrak{I}_j)]$ decreases to 0 while $\widetilde{\mathrm{E}}[g_{i,2}(\mathfrak{I}_j)]$ increases to 1. We can also observe the following relationship: $\widetilde{\mathrm{E}}[g_{i,2}(\mathfrak{I}_{j+1})] - \widetilde{\mathrm{E}}[g_{i,2}(\mathfrak{I}_j)] = \widetilde{\mathrm{E}}[\delta_{i,1\to2}(\mathfrak{I}_j)]$. This is because $\widetilde{\mathrm{E}}[g_{i,2}(\mathfrak{I}_{j+1})]$ is the probability of being in State 2 right before the next interval, $\widetilde{\mathrm{E}}[g_{i,2}(\mathfrak{I}_j)]$ is the probability of being in State 2 right before the current interval, their difference is naturally because of the transitions occurring in the current interval $\widetilde{\mathrm{E}}[\delta_{i,1\to2}(\mathfrak{I}_j)]$.

The second individual transitions from State 1 to State 2 in $\mathfrak{I}_{14}$ and from State 2 to State 3 in $\mathfrak{I}_{16}$. However, we only know that the transitions occurred between T_{i1} and T_{i2}. Similarly, the unobserved truth $g_{i,1}(\mathfrak{I}_j)$, $g_{i,2}(\mathfrak{I}_j)$, $g_{i,3}(\mathfrak{I}_j)$, $\delta_{i,1\to2}(\mathfrak{I}_j)$, $\delta_{i,2\to3}(\mathfrak{I}_j)$ are inferred in the estimation procedure by the quantities $\widetilde{\mathrm{E}}[g_{i,1}(\mathfrak{I}_j)]$, $\widetilde{\mathrm{E}}[g_{i,2}(\mathfrak{I}_j)]$, $\widetilde{\mathrm{E}}[g_{i,3}(\mathfrak{I}_j)]$, $\widetilde{\mathrm{E}}[\delta_{i,1\to2}(\mathfrak{I}_j)]$, $\widetilde{\mathrm{E}}[\delta_{i,2\to3}(\mathfrak{I}_j)]$.

S6.2 Illustrating Example 2

Here, we first consider a more complicated 6-state model as illustrated in Figure S2b. Two individuals (Figure S4 and Figure S5) in the simulated dataset are presented along with the quantities of $\mathfrak{I}_j$, τ_j, $s_i(\mathfrak{I}_j)$, $g_{i,1}(\mathfrak{I}_j)$, $g_{i,2}(\mathfrak{I}_j)$, $g_{i,3}(\mathfrak{I}_j)$, $\delta_{i,1\to2}(\mathfrak{I}_j)$, $\delta_{i,2\to3}(\mathfrak{I}_j)$, S_{ij}, T_{ij}, $\widetilde{\mathrm{E}}[g_{i,1}(\mathfrak{I}_j)]$, $\widetilde{\mathrm{E}}[g_{i,2}(\mathfrak{I}_j)]$, $\widetilde{\mathrm{E}}[g_{i,3}(\mathfrak{I}_j)]$, $\widetilde{\mathrm{E}}[\delta_{i,1\to2}(\mathfrak{I}_j)]$, $\widetilde{\mathrm{E}}[\delta_{i,2\to3}(\mathfrak{I}_j)]$ from top to bottom, where the quantities of $\widetilde{\mathrm{E}}[g_{i,3}(\mathfrak{I}_j)]$, $\widetilde{\mathrm{E}}[\delta_{i,1\to2}(\mathfrak{I}_j)]$, $\widetilde{\mathrm{E}}[\delta_{i,2\to3}(\mathfrak{I}_j)]$ are the estimated quantities at convergence.

In this example, $N_t = 20$, $N_y = 1$, $N_z = 1$, $h_{0,s_1\to s_2}(t) = 0.5 + (s_2 - s_1)\sin(10 \times t + s_1 + s_2)$, $\mathbf{z}_i$ follow independent standard normal distribution, $B_{kl}(t)$ are linear basis functions, $\mathbf{c} = (0, 0)^\mathsf{T}$, and

$$\boldsymbol{\Sigma}_a = \begin{pmatrix} 1 & 0 \\ 0 & 0.2 \end{pmatrix}.$$

S7 Additional Details and Results on Simulation Studies

S7.1 Details on the generation of simulation data

In the simulation studies, the multistate data were generated using the discrete-time method. We let $\mathbb{T} = \{\tau_0, \ldots, \tau_{N_t}\}$ be equally spaced timepoints in the design interval $[0, \mathcal{T}]$. After simulating the quantities of $\boldsymbol{\eta}_{i,s_1 \to s_2}(\mathfrak{I}_j)$, the processes $s_i(t)$ are governed by the following equation

$$\mathrm{P}\left(s_i(\tau_{j+1}) = s_2 \middle| s_i(\tau_j) = s_1\right) = 1 - \exp\left\{ -(\tau_{j+1} - \tau_j) h_{0,s_1 \to s_2}(\tau_j) \exp\left[\boldsymbol{\beta}_{s_1 \to s_2}^{\mathsf{T}} \boldsymbol{\eta}_{i,s_1 \to s_2}(\tau_j)\right] \right\}$$

when $s_1 \neq s_2$. If we let $\max_{|\tau_{j+1} - \tau_j|} \to 0$, the processes $s_i(t)$ can be viewed as realizations of the following transition rate matrix

$$\begin{cases} h_{0,s_1 \to s_2}(t) \exp\left[\boldsymbol{\beta}_{s_1 \to s_2}^{\mathsf{T}} \boldsymbol{\eta}_{i,s_1 \to s_2}(t)\right] & \text{when } s_1 \neq s_2 \\ -\sum_{s: s \neq s_1} h_{0,s_1 \to s}(t) \exp\left[\boldsymbol{\beta}_{s_1 \to s}^{\mathsf{T}} \boldsymbol{\eta}_{i,s_1 \to s}(t)\right] & \text{when } s_1 = s_2. \end{cases}$$

S7.2 Additional results for model parameter estimates

Next, we present other estimated parameters in the simulation studies. The estimated $\mathbf{c}$ and σ_k^2 in Simulations 1 and 2 are provided in Table S2. The estimated $\boldsymbol{\Sigma}_a$ are presented in Table S3.

S7.3 Methods for statistical inference on $\boldsymbol{\beta}_{s_1 \to s_2}$

In many applications, there is interest in making inferences on the coefficients $\boldsymbol{\beta}_{s_1 \to s_2}$. In this subsection, we present two methods that have been explored in this research.

The first method is to use the information matrix obtained from the second derivatives of $\widetilde{\mathrm{E}}[l_c^{(1)}]$ (c.f., You et al. 2024). We refer to this approach hereafter as the profile-likelihood method. The information matrix for $\boldsymbol{\beta}_{s_1 \to s_2}$ can be obtained by

$$\mathbf{I}_c^{(1)}(\boldsymbol{\beta}_{s_1 \to s_2}) = -\frac{\partial^2 \widetilde{\mathrm{E}}[l_c^{(1)}]}{(\partial \boldsymbol{\beta}_{s_1 \to s_2})^2}.$$

Design intervals: τ_0, τ_1, τ_2, τ_3, τ_4, τ_5, τ_6, τ_7, τ_8, τ_9, τ_{10}, τ_{11}, τ_{12}, τ_{13}, τ_{14}, τ_{15}, τ_{16}, τ_{17}, τ_{18}, τ_{19}, τ_{20} (τ_j)

Observed Data: S_{ij}: State 1 $S_{i0} = \{1\}$ (at τ_0); State 1 $S_{i1} = \{1\}$ (at τ_4); State 6 $S_{i2} = \{6\}$ (at τ_{12}). T_{ij}: T_{i0} (at τ_0), T_{i1} (at τ_4), T_{i2} (at τ_{12}).

	I_j	I_1	I_2	I_3	I_4	I_5	I_6	I_7	I_8	I_9	I_{10}	I_{11}	I_{12}	I_{13}	I_{14}	I_{15}	I_{16}	I_{17}	I_{18}	I_{19}	I_{20}
Unobserved Truth	$s_i(I_j)$	1	1	1	1	(1−>2) 2	2	2	2	2	(2−>5) 5	5	(5−>6) 6	6	6	6	6	6	6	6	6
	$g_{i,1}(I_j)$	1	1	1	1	1	0	0	0	0	0	0	0	0	0	0	0	0	0	0	0
	$g_{i,2}(I_j)$	0	0	0	0	0	1	1	1	1	1	0	0	0	0	0	0	0	0	0	0
	$g_{i,3}(I_j)$	0	0	0	0	0	0	0	0	0	0	0	0	0	0	0	0	0	0	0	0
	$g_{i,4}(I_j)$	0	0	0	0	0	0	0	0	0	0	0	0	0	0	0	0	0	0	0	0
	$g_{i,5}(I_j)$	0	0	0	0	0	0	0	0	0	0	1	1	0	0	0	0	0	0	0	0
	$g_{i,6}(I_j)$	0	0	0	0	0	0	0	0	0	0	0	0	1	1	1	1	1	1	1	1
	$\delta_{i,1\to2}(I_j)$	0	0	0	0	1	0	0	0	0	0	0	0	0	0	0	0	0	0	0	0
	$\delta_{i,1\to3}(I_j)$	0	0	0	0	0	0	0	0	0	0	0	0	0	0	0	0	0	0	0	0
	$\delta_{i,2\to4}(I_j)$	0	0	0	0	0	0	0	0	0	0	0	0	0	0	0	0	0	0	0	0
	$\delta_{i,2\to5}(I_j)$	0	0	0	0	0	0	0	0	0	1	0	0	0	0	0	0	0	0	0	0
	$\delta_{i,3\to4}(I_j)$	0	0	0	0	0	0	0	0	0	0	0	0	0	0	0	0	0	0	0	0
	$\delta_{i,3\to5}(I_j)$	0	0	0	0	0	0	0	0	0	0	0	0	0	0	0	0	0	0	0	0
	$\delta_{i,4\to6}(I_j)$	0	0	0	0	0	0	0	0	0	0	0	1	0	0	0	0	0	0	0	0
	$\delta_{i,5\to6}(I_j)$	0	0	0	0	0	0	0	0	0	0	0	1	0	0	0	0	0	0	0	0
Estimated Information	$\tilde{E}_i[g_{i,1}(I_j)]$	1.00	1.00	1.00	1.00	1.00	0.64	0.23	0.13	0.00	0.00	0.00	0.00	0.00	0.00	0.00	0.00	0.00	0.00	0.00	0.00
	$\tilde{E}_i[g_{i,2}(I_j)]$	0.00	0.00	0.00	0.00	0.00	0.31	0.51	0.56	0.69	0.69	0.00	0.00	0.00	0.00	0.00	0.00	0.00	0.00	0.00	0.00
	$\tilde{E}_i[g_{i,3}(I_j)]$	0.00	0.00	0.00	0.00	0.00	0.04	0.04	0.09	0.00	0.00	0.00	0.00	0.00	0.00	0.00	0.00	0.00	0.00	0.00	0.00
	$\tilde{E}_i[g_{i,4}(I_j)]$	0.00	0.00	0.00	0.00	0.00	0.00	0.00	0.00	0.00	0.00	0.00	0.00	0.00	0.00	0.00	0.00	0.00	0.00	0.00	0.00
	$\tilde{E}_i[g_{i,5}(I_j)]$	0.00	0.00	0.00	0.00	0.00	0.00	0.21	0.21	0.31	0.31	1.00	0.00	0.00	0.00	0.00	0.00	0.00	0.00	0.00	0.00
	$\tilde{E}_i[g_{i,6}(I_j)]$	0.00	0.00	0.00	0.00	0.00	0.01	0.01	0.01	0.00	0.00	0.00	1.00	1.00	1.00	1.00	1.00	1.00	1.00	1.00	1.00
	$\tilde{E}_i[\delta_{i,1\to2}(I_j)]$	0.00	0.00	0.00	0.00	0.31	0.41	0.05	0.13	0.00	0.00	0.00	0.00	0.00	0.00	0.00	0.00	0.00	0.00	0.00	0.00
	$\tilde{E}_i[\delta_{i,1\to3}(I_j)]$	0.00	0.00	0.00	0.00	0.04	0.00	0.05	0.00	0.00	0.00	0.00	0.00	0.00	0.00	0.00	0.00	0.00	0.00	0.00	0.00
	$\tilde{E}_i[\delta_{i,2\to4}(I_j)]$	0.00	0.00	0.00	0.00	0.00	0.00	0.00	0.00	0.00	0.00	0.00	0.00	0.00	0.00	0.00	0.00	0.00	0.00	0.00	0.00
	$\tilde{E}_i[\delta_{i,2\to5}(I_j)]$	0.00	0.00	0.00	0.00	0.00	0.21	0.00	0.00	0.00	0.69	0.00	0.00	0.00	0.00	0.00	0.00	0.00	0.00	0.00	0.00
	$\tilde{E}_i[\delta_{i,3\to4}(I_j)]$	0.00	0.00	0.00	0.00	0.00	0.00	0.00	0.00	0.00	0.00	0.00	0.00	0.00	0.00	0.00	0.00	0.00	0.00	0.00	0.00
	$\tilde{E}_i[\delta_{i,3\to5}(I_j)]$	0.00	0.00	0.00	0.00	0.00	0.00	0.00	0.09	0.00	0.00	0.00	0.00	0.00	0.00	0.00	0.00	0.00	0.00	0.00	0.00
	$\tilde{E}_i[\delta_{i,4\to6}(I_j)]$	0.00	0.00	0.00	0.00	0.00	0.00	0.00	0.00	0.00	0.00	0.00	0.00	0.00	0.00	0.00	0.00	0.00	0.00	0.00	0.00
	$\tilde{E}_i[\delta_{i,5\to6}(I_j)]$	0.00	0.00	0.00	0.00	0.00	0.00	0.00	0.00	0.00	0.00	1.00	0.00	0.00	0.00	0.00	0.00	0.00	0.00	0.00	0.00

Figure S4: Parameters of the first individual in Illustrating Example 2.

Design intervals

τ_j: τ_0, τ_1, τ_2, τ_3, τ_4, τ_5, τ_6, τ_7, τ_8, τ_9, τ_{10}, τ_{11}, τ_{12}, τ_{13}, τ_{14}, τ_{15}, τ_{16}, τ_{17}, τ_{18}, τ_{19}, τ_{20}

Unobserved Truth

I_j	I_1	I_2	I_3	I_4	I_5	I_6	I_7	I_8	I_9	I_{10}	I_{11}	I_{12}	I_{13}	I_{14}	I_{15}	I_{16}	I_{17}	I_{18}	I_{19}	I_{20}
$s_i(I_j)$	(1−>2) 2	2	(2−>5) 5	(5−>6) 6	6	6	6	6	6	6	6	6	6	6	6	6	6	6	6	6
$g_{i,1}(I_j)$	1	0	0	0	0	0	0	0	0	0	0	0	0	0	0	0	0	0	0	0
$g_{i,2}(I_j)$	0	1	1	0	0	0	0	0	0	0	0	0	0	0	0	0	0	0	0	0
$g_{i,3}(I_j)$	0	0	0	0	0	0	0	0	0	0	0	0	0	0	0	0	0	0	0	0
$g_{i,4}(I_j)$	0	0	0	0	0	0	0	0	0	0	0	0	0	0	0	0	0	0	0	0
$g_{i,5}(I_j)$	0	0	0	1	0	0	0	0	0	0	0	0	0	0	0	0	0	0	0	0
$g_{i,6}(I_j)$	0	0	0	0	1	1	1	1	1	1	1	1	1	1	1	1	1	1	1	1
$\delta_{i,1\to2}(I_j)$	1	0	0	0	0	0	0	0	0	0	0	0	0	0	0	0	0	0	0	0
$\delta_{i,1\to3}(I_j)$	0	0	0	0	0	0	0	0	0	0	0	0	0	0	0	0	0	0	0	0
$\delta_{i,2\to4}(I_j)$	0	0	0	0	0	0	0	0	0	0	0	0	0	0	0	0	0	0	0	0
$\delta_{i,2\to5}(I_j)$	0	0	1	0	0	0	0	0	0	0	0	0	0	0	0	0	0	0	0	0
$\delta_{i,3\to4}(I_j)$	0	0	0	0	0	0	0	0	0	0	0	0	0	0	0	0	0	0	0	0
$\delta_{i,3\to5}(I_j)$	0	0	0	0	0	0	0	0	0	0	0	0	0	0	0	0	0	0	0	0
$\delta_{i,4\to6}(I_j)$	0	0	0	1	0	0	0	0	0	0	0	0	0	0	0	0	0	0	0	0
$\delta_{i,5\to6}(I_j)$	0	0	0	1	0	0	0	0	0	0	0	0	0	0	0	0	0	0	0	0

Observed Data

S_{ij}: State 1, $S_{i0} = \{1\}$ (at τ_0); State 6, $S_{i1} = \{6\}$ (at τ_6)

T_{ij}: T_{i0} (at τ_0); T_{i1} (at τ_6)

Estimated Information

	I_1	I_2	I_3	I_4	I_5	I_6	I_7	I_8	I_9	I_{10}	I_{11}	I_{12}	I_{13}	I_{14}	I_{15}	I_{16}	I_{17}	I_{18}	I_{19}	I_{20}
$\tilde{E}_i[g_{i,1}(I_j)]$	1.00	0.90	0.08	0.02	0.00	0.00	0.00	0.00	0.00	0.00	0.00	0.00	0.00	0.00	0.00	0.00	0.00	0.00	0.00	0.00
$\tilde{E}_i[g_{i,2}(I_j)]$	0.00	0.10	0.87	0.35	0.07	0.00	0.00	0.00	0.00	0.00	0.00	0.00	0.00	0.00	0.00	0.00	0.00	0.00	0.00	0.00
$\tilde{E}_i[g_{i,3}(I_j)]$	0.00	0.00	0.00	0.00	0.00	0.00	0.00	0.00	0.00	0.00	0.00	0.00	0.00	0.00	0.00	0.00	0.00	0.00	0.00	0.00
$\tilde{E}_i[g_{i,4}(I_j)]$	0.00	0.00	0.05	0.04	0.00	0.00	0.00	0.00	0.00	0.00	0.00	0.00	0.00	0.00	0.00	0.00	0.00	0.00	0.00	0.00
$\tilde{E}_i[g_{i,5}(I_j)]$	0.00	0.00	0.00	0.59	0.53	0.60	0.00	0.00	0.00	0.00	0.00	0.00	0.00	0.00	0.00	0.00	0.00	0.00	0.00	0.00
$\tilde{E}_i[g_{i,6}(I_j)]$	0.00	−0.00	0.00	0.00	0.40	0.40	1.00	1.00	1.00	1.00	1.00	1.00	1.00	1.00	1.00	1.00	1.00	1.00	1.00	1.00
$\tilde{E}_i[\delta_{i,1\to2}(I_j)]$	0.10	0.81	0.07	0.02	0.00	0.00	0.00	0.00	0.00	0.00	0.00	0.00	0.00	0.00	0.00	0.00	0.00	0.00	0.00	0.00
$\tilde{E}_i[\delta_{i,1\to3}(I_j)]$	0.00	0.00	0.00	0.00	0.00	0.00	0.00	0.00	0.00	0.00	0.00	0.00	0.00	0.00	0.00	0.00	0.00	0.00	0.00	0.00
$\tilde{E}_i[\delta_{i,2\to4}(I_j)]$	0.00	0.05	0.00	0.00	0.00	0.00	0.00	0.00	0.00	0.00	0.00	0.00	0.00	0.00	0.00	0.00	0.00	0.00	0.00	0.00
$\tilde{E}_i[\delta_{i,2\to5}(I_j)]$	0.00	0.00	0.59	0.30	0.07	0.00	0.00	0.00	0.00	0.00	0.00	0.00	0.00	0.00	0.00	0.00	0.00	0.00	0.00	0.00
$\tilde{E}_i[\delta_{i,3\to4}(I_j)]$	0.00	0.00	0.00	0.00	0.00	0.00	0.00	0.00	0.00	0.00	0.00	0.00	0.00	0.00	0.00	0.00	0.00	0.00	0.00	0.00
$\tilde{E}_i[\delta_{i,3\to5}(I_j)]$	0.00	0.00	0.00	0.00	0.00	0.00	0.00	0.00	0.00	0.00	0.00	0.00	0.00	0.00	0.00	0.00	0.00	0.00	0.00	0.00
$\tilde{E}_i[\delta_{i,4\to6}(I_j)]$	0.00	0.00	0.01	0.04	0.00	0.00	0.00	0.00	0.00	0.00	0.00	0.00	0.00	0.00	0.00	0.00	0.00	0.00	0.00	0.00
$\tilde{E}_i[\delta_{i,5\to6}(I_j)]$	0.00	0.00	0.00	0.36	0.00	0.60	0.00	0.00	0.00	0.00	0.00	0.00	0.00	0.00	0.00	0.00	0.00	0.00	0.00	0.00

Figure S5: Parameters of the second individual in Illustrating Example 2.

Table S2: Estimated $\mathbf{c}$ and σ_k^2 in Simulations 1 and 2.

(a) Simulation 1

Parameter	Estimate (truth)
$[\![\mathbf{c}]\!]_{\{1\}}$	0.2011 (0.20)
$[\![\mathbf{c}]\!]_{\{2\}}$	0.0984 (0.10)
$[\![\mathbf{c}]\!]_{\{3\}}$	0.0001 (0.00)
$[\![\mathbf{c}]\!]_{\{4\}}$	0.0013 (0.00)
$[\![\mathbf{c}]\!]_{\{5\}}$	-0.0005 (0.00)
$[\![\mathbf{c}]\!]_{\{6\}}$	0.0002 (0.00)
$[\![\mathbf{c}]\!]_{\{7\}}$	0.2025 (0.20)
$[\![\mathbf{c}]\!]_{\{8\}}$	0.1010 (0.10)
$\sigma^2_{\{1\}}$	1.0008 (1.00)
$\sigma^2_{\{2\}}$	0.9993 (1.00)

(b) Simulation 2

Parameter	Estimate (truth)
$[\![\mathbf{c}]\!]_{\{1\}}$	0.3007 (0.30)
$[\![\mathbf{c}]\!]_{\{2\}}$	0.1990 (0.20)
$[\![\mathbf{c}]\!]_{\{3\}}$	0.0997 (0.10)
$[\![\mathbf{c}]\!]_{\{4\}}$	0.0013 (0.00)
$[\![\mathbf{c}]\!]_{\{5\}}$	0.3009 (0.30)
$[\![\mathbf{c}]\!]_{\{6\}}$	0.1996 (0.20)
$[\![\mathbf{c}]\!]_{\{7\}}$	0.1003 (0.10)
$\sigma^2_{\{1\}}$	1.0001 (1.00)
$\sigma^2_{\{2\}}$	1.0008 (1.00)

The variance-covariance matrix of $\widehat{\boldsymbol{\beta}}_{s_1\to s_2}$ can be approximated by the inverse of $\mathbf{I}_c^{(1)}(\boldsymbol{\beta}_{s_1\to s_2})$ evaluated using the estimated parameters, which we denote by $\mathbf{V}_{s_1\to s_2} = \left[\mathbf{I}_c^{(1)}(\boldsymbol{\beta}_{s_1\to s_2})\right]^{-1}\big|_{\boldsymbol{\Theta}=\widehat{\boldsymbol{\Theta}}}$, where we use the notation $\widehat{\cdot}$ to denote the estimated parameters. Let ρ be a set of indices corresponding to the coefficients in $\boldsymbol{\beta}_{s_1\to s_2}$ of interest. A Wald test for the null hypothesis that $[\![\widehat{\boldsymbol{\beta}}_{s_1\to s_2}]\!]_\rho = \boldsymbol{\beta}^{(0)}$ can be conducted using the Wald test statistic

$$W = \left([\![\widehat{\boldsymbol{\beta}}_{s_1\to s_2}]\!]_\rho - \boldsymbol{\beta}^{(0)}\right)^{\mathsf{T}}\left([\![\mathbf{V}_{s_1\to s_2}]\!]_{\rho,\rho}\right)^{-1}\left([\![\widehat{\boldsymbol{\beta}}_{s_1\to s_2}]\!]_\rho - \boldsymbol{\beta}^{(0)}\right),$$

where the p-values can be obtained by comparing W to the upper tail of a chi-square distribution with $|\rho|$ degrees of freedom. The $(1-\alpha)\times 100\%$ confidence interval for $[\![\boldsymbol{\beta}_{s_1\to s_2}]\!]_\rho$ when ρ contains a single index of interest is then given by $[\![\widehat{\boldsymbol{\beta}}_{s_1\to s_2}]\!]_\rho \pm z_{\alpha/2}\times\sqrt{[\![\mathbf{V}_{s_1\to s_2}]\!]_{\rho,\rho}}$, where $z_{\alpha/2}$ is the $(1-\alpha/2)$-quantile of the standard normal distributions, and $\sqrt{[\![\mathbf{V}_{s_1\to s_2}]\!]_{\rho,\rho}}$ serves as an estimate of the standard error for $[\![\widehat{\boldsymbol{\beta}}_{s_1\to s_2}]\!]_\rho$.

However, as pointed out by several existing works (c.f., Hsieh et al. 2006), the profile-likelihood method oversimplifies the fact that the conditional expectations $\widetilde{\mathrm{E}}[\cdot]$ also depend on the parameter $\boldsymbol{\beta}_{s_1\to s_2}$. As a result, it may produce confidence intervals with undercoverage. We will later use

Table S3: Estimated $\boldsymbol{\Sigma}_a$ in Simulations 1 and 2. Numbers in the parentheses are the true values.

(a) Simulation 1

	Column 1	Column 2	Column 3	Column 4	Column 5	Column 6	Column 7	Column 8
Row 1	0.9973 (1.00)	0.0004 (0.00)	0.0001 (0.00)	-0.0004 (0.00)	0.0007 (0.00)	-0.0002 (0.00)	0.0016 (0.00)	0.0009 (0.00)
Row 2	0.0004 (0.00)	0.2489 (0.25)	0.0005 (0.00)	0.0001 (0.00)	-0.0001 (0.00)	0.0000 (0.00)	0.0009 (0.00)	0.0010 (0.00)
Row 3	0.0001 (0.00)	0.0005 (0.00)	0.1600 (0.16)	0.0003 (0.00)	-0.0003 (0.00)	0.0002 (0.00)	-0.0009 (0.00)	0.0001 (0.00)
Row 4	-0.0004 (0.00)	0.0001 (0.00)	0.0003 (0.00)	0.0896 (0.09)	-0.0004 (0.00)	0.0001 (0.00)	-0.0011 (0.00)	-0.0002 (0.00)
Row 5	0.0007 (0.00)	-0.0001 (0.00)	-0.0003 (0.00)	-0.0004 (0.00)	0.0398 (0.04)	0.0000 (0.00)	0.0003 (0.00)	-0.0003 (0.00)
Row 6	-0.0002 (0.00)	0.0000 (0.00)	0.0002 (0.00)	0.0001 (0.00)	0.0000 (0.00)	0.0101 (0.01)	-0.0001 (0.00)	0.0003 (0.00)
Row 7	0.0016 (0.00)	0.0009 (0.00)	-0.0009 (0.00)	-0.0011 (0.00)	0.0003 (0.00)	-0.0001 (0.00)	0.9986 (1.00)	0.0017 (0.00)
Row 8	0.0009 (0.00)	0.0010 (0.00)	0.0001 (0.00)	-0.0002 (0.00)	-0.0003 (0.00)	0.0003 (0.00)	0.0017 (0.00)	0.2504 (0.25)

(b) Simulation 2

	Column 1	Column 2	Column 3	Column 4	Column 5	Column 6	Column 7
Row 1	0.6472 (0.65)	0.0501 (0.05)	0.0500 (0.05)	0.0495 (0.05)	0.0013 (0.00)	0.0001 (0.00)	0.0011 (0.00)
Row 2	0.0501 (0.05)	0.3484 (0.35)	0.0505 (0.05)	0.0502 (0.05)	-0.0004 (0.00)	-0.0001 (0.00)	-0.0000 (0.00)
Row 3	0.0500 (0.05)	0.0505 (0.05)	0.2497 (0.25)	0.0501 (0.05)	-0.0016 (0.00)	-0.0003 (0.00)	0.0003 (0.00)
Row 4	0.0495 (0.05)	0.0502 (0.05)	0.0501 (0.05)	0.1498 (0.15)	-0.0010 (0.00)	0.0010 (0.00)	-0.0001 (0.00)
Row 5	0.0013 (0.00)	-0.0004 (0.00)	-0.0016 (0.00)	-0.0010 (0.00)	0.6467 (0.65)	0.0502 (0.05)	0.0506 (0.05)
Row 6	0.0001 (0.00)	-0.0001 (0.00)	-0.0003 (0.00)	0.0010 (0.00)	0.0502 (0.05)	0.3480 (0.35)	0.0506 (0.05)
Row 7	0.0011 (0.00)	-0.0000 (0.00)	0.0003 (0.00)	-0.0001 (0.00)	0.0506 (0.05)	0.0506 (0.05)	0.2504 (0.25)

some simulation studies to investigate it.

The second method is to use bootstrap confidence intervals. We refer to this approach hereafter as the bootstrap method. Under this approach, we resample individuals from the original training dataset to generate a new dataset of the same size and apply the estimation procedure to obtain bootstrap estimates of the parameters. This process is repeated B times. The $(1-\alpha)\times 100\%$ confidence interval for each parameter is then constructed using the $\alpha/2$ and $(1-\alpha/2)$ quantiles of the bootstrap estimates. Although bootstrap is a preferred method in many joint modeling applications, it requires repeatedly fitting the model, which is often computationally intensive and time-consuming.

We conducted simulation studies to evaluate the performance of the two methods for constructing confidence intervals. In Simulations 1 and 2, we assessed the profile-likelihood method. Due to computational limitations, the bootstrap method was evaluated only in Simulation 1, using 200 bootstrap samples. The coverage rates of the 95% confidence intervals based on the information matrix are reported in Table S4 for Simulation 1 and Table S5 for Simulation 2. The results indicate that confidence intervals constructed using the profile-likelihood method tended to have lower coverage than the nominal level. In contrast, the bootstrap method produced confidence intervals with coverage closer to the nominal probability in Simulation 1.

Table S4: Coverage rate of 95% Confidence intervals (CI) using the profile-likelihood and bootstrap method in Simulation 1.

Parameter	profile-likelihood CI coverage	Bootstrap CI coverage	Parameter	profile-likelihood CI coverage	Bootstrap CI coverage
$[\![\boldsymbol{\beta}_{1\to2}]\!]_{\{1\}}$	0.918	0.932	$[\![\boldsymbol{\beta}_{2\to3}]\!]_{\{4\}}$	0.940	0.952
$[\![\boldsymbol{\beta}_{1\to2}]\!]_{\{3\}}$	0.944	0.956	$[\![\boldsymbol{\beta}_{2\to4}]\!]_{\{1\}}$	0.848	0.936
$[\![\boldsymbol{\beta}_{1\to2}]\!]_{\{4\}}$	0.940	0.950	$[\![\boldsymbol{\beta}_{2\to4}]\!]_{\{2\}}$	0.858	0.960
$[\![\boldsymbol{\beta}_{1\to4}]\!]_{\{1\}}$	0.930	0.960	$[\![\boldsymbol{\beta}_{2\to4}]\!]_{\{3\}}$	0.894	0.964
$[\![\boldsymbol{\beta}_{1\to4}]\!]_{\{3\}}$	0.908	0.928	$[\![\boldsymbol{\beta}_{2\to4}]\!]_{\{4\}}$	0.876	0.938
$[\![\boldsymbol{\beta}_{1\to4}]\!]_{\{4\}}$	0.954	0.956	$[\![\boldsymbol{\beta}_{3\to4}]\!]_{\{1\}}$	0.888	0.958
$[\![\boldsymbol{\beta}_{2\to3}]\!]_{\{1\}}$	0.878	0.938	$[\![\boldsymbol{\beta}_{3\to4}]\!]_{\{2\}}$	0.914	0.954
$[\![\boldsymbol{\beta}_{2\to3}]\!]_{\{2\}}$	0.878	0.910	$[\![\boldsymbol{\beta}_{3\to4}]\!]_{\{3\}}$	0.918	0.946
$[\![\boldsymbol{\beta}_{2\to3}]\!]_{\{3\}}$	0.946	0.970	$[\![\boldsymbol{\beta}_{3\to4}]\!]_{\{4\}}$	0.896	0.930

Table S5: Coverage rate of 95% Confidence intervals (CI) using the provile-likelihood method in Simulation 2.

Parameter	profile-likelihood CI coverage	Parameter	profile-likelihood CI coverage	Parameter	profile-likelihood CI coverage	Parameter	profile-likelihood CI coverage
$[\![\boldsymbol{\beta}_{1\to2}]\!]_{\{1\}}$	0.896	$[\![\boldsymbol{\beta}_{2\to4}]\!]_{\{1\}}$	0.846	$[\![\boldsymbol{\beta}_{3\to4}]\!]_{\{1\}}$	0.856	$[\![\boldsymbol{\beta}_{4\to6}]\!]_{\{1\}}$	0.904
$[\![\boldsymbol{\beta}_{1\to2}]\!]_{\{2\}}$	0.862	$[\![\boldsymbol{\beta}_{2\to4}]\!]_{\{2\}}$	0.866	$[\![\boldsymbol{\beta}_{3\to4}]\!]_{\{2\}}$	0.852	$[\![\boldsymbol{\beta}_{4\to6}]\!]_{\{2\}}$	0.892
$[\![\boldsymbol{\beta}_{1\to2}]\!]_{\{3\}}$	0.946	$[\![\boldsymbol{\beta}_{2\to4}]\!]_{\{3\}}$	0.896	$[\![\boldsymbol{\beta}_{3\to4}]\!]_{\{3\}}$	0.904	$[\![\boldsymbol{\beta}_{4\to6}]\!]_{\{3\}}$	0.922
$[\![\boldsymbol{\beta}_{1\to2}]\!]_{\{4\}}$	0.914	$[\![\boldsymbol{\beta}_{2\to4}]\!]_{\{4\}}$	0.902	$[\![\boldsymbol{\beta}_{3\to4}]\!]_{\{4\}}$	0.898	$[\![\boldsymbol{\beta}_{4\to6}]\!]_{\{4\}}$	0.914
$[\![\boldsymbol{\beta}_{1\to3}]\!]_{\{1\}}$	0.854	$[\![\boldsymbol{\beta}_{2\to5}]\!]_{\{1\}}$	0.872	$[\![\boldsymbol{\beta}_{3\to5}]\!]_{\{1\}}$	0.862	$[\![\boldsymbol{\beta}_{5\to6}]\!]_{\{1\}}$	0.904
$[\![\boldsymbol{\beta}_{1\to3}]\!]_{\{2\}}$	0.906	$[\![\boldsymbol{\beta}_{2\to5}]\!]_{\{2\}}$	0.884	$[\![\boldsymbol{\beta}_{3\to5}]\!]_{\{2\}}$	0.852	$[\![\boldsymbol{\beta}_{5\to6}]\!]_{\{2\}}$	0.878
$[\![\boldsymbol{\beta}_{1\to3}]\!]_{\{3\}}$	0.934	$[\![\boldsymbol{\beta}_{2\to5}]\!]_{\{3\}}$	0.922	$[\![\boldsymbol{\beta}_{3\to5}]\!]_{\{3\}}$	0.890	$[\![\boldsymbol{\beta}_{5\to6}]\!]_{\{3\}}$	0.914
$[\![\boldsymbol{\beta}_{1\to3}]\!]_{\{4\}}$	0.924	$[\![\boldsymbol{\beta}_{2\to5}]\!]_{\{4\}}$	0.902	$[\![\boldsymbol{\beta}_{3\to5}]\!]_{\{4\}}$	0.896	$[\![\boldsymbol{\beta}_{5\to6}]\!]_{\{4\}}$	0.918

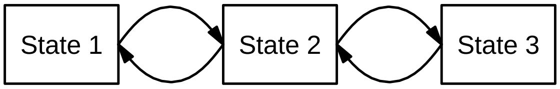

Figure S6: Multistate model for Simulation 4

S8 Additional simulation studies

S8.1 Simulation 3

This simulation study aims to investigate the multistate model used in the real data application (i.e., Figure 1 in the main text). In this example, $N_y = 2$, $N_z = 2$, $N_t = 200$, $\mathbf{c} = \mathbf{0}_{4\times1}$, $\boldsymbol{\Sigma}_a = \text{diag}\left\{\begin{pmatrix}1.0 & 0.2\\0.2 & 1.0\end{pmatrix}, \begin{pmatrix}1.0 & 0.2\\0.2 & 1.0\end{pmatrix}\right\}$, $h_{0,s_1\to s_2}(t) = 0.8^{|s_2-s_1|}$, $t_{ik,j+1} - t_{ikj} - 0.05 \sim \text{Gamma}(3, 0.01)$ and $\text{P}(s \neq s_i(t), s \in S_{ij}) = 0.02$. The estimated $\boldsymbol{\beta}_{s_1\to s_2}$ are presented in Table S6 and the estimated $\mathbf{c}$, σ_k and $\boldsymbol{\Sigma}_a$ are presented in Table S7. In this example, the second dimension of $\boldsymbol{\eta}_i(t)$ (i.e., $m_{i2}(t)$) is only included in the multistate model after leaving State 1 (similarly as glucose is only measured after the development of the second autoantibody in the TEDDY data application).

Table S6: True values of regression coefficients and the biases of estimated regression coefficients in Simulation 3.

Parameter	Truth	Proposed	MSM	Parameter	Truth	Proposed	MSM
$\llbracket \boldsymbol{\beta}_{1\to2} \rrbracket_{\{1\}}$	0.5	-0.003 (0.004)	-0.002 (0.003)	$\llbracket \boldsymbol{\beta}_{4\to6} \rrbracket_{\{3\}}$	0.3	-0.001 (0.019)	-0.267 (0.002)
$\llbracket \boldsymbol{\beta}_{1\to2} \rrbracket_{\{2\}}$	0.3	0.001 (0.007)	-0.267 (0.001)	$\llbracket \boldsymbol{\beta}_{4\to6} \rrbracket_{\{4\}}$	0.5	0.032 (0.019)	-0.444 (0.002)
$\llbracket \boldsymbol{\beta}_{1\to2} \rrbracket_{\{3\}}$	0.3	0.006 (0.008)	-0.267 (0.001)	$\llbracket \boldsymbol{\beta}_{4\to7} \rrbracket_{\{1\}}$	0.2	0.005 (0.009)	0.006 (0.008)
$\llbracket \boldsymbol{\beta}_{1\to3} \rrbracket_{\{1\}}$	0.2	0.001 (0.004)	0.005 (0.004)	$\llbracket \boldsymbol{\beta}_{4\to7} \rrbracket_{\{2\}}$	0.2	0.009 (0.008)	0.006 (0.008)
$\llbracket \boldsymbol{\beta}_{1\to3} \rrbracket_{\{2\}}$	0.5	-0.008 (0.008)	-0.447 (0.001)	$\llbracket \boldsymbol{\beta}_{4\to7} \rrbracket_{\{3\}}$	0.3	-0.024 (0.021)	-0.270 (0.002)
$\llbracket \boldsymbol{\beta}_{1\to3} \rrbracket_{\{3\}}$	0.3	0.012 (0.008)	-0.266 (0.001)	$\llbracket \boldsymbol{\beta}_{4\to7} \rrbracket_{\{4\}}$	0.3	0.053 (0.020)	-0.263 (0.002)
$\llbracket \boldsymbol{\beta}_{1\to4} \rrbracket_{\{1\}}$	0.2	-0.001 (0.004)	0.000 (0.004)	$\llbracket \boldsymbol{\beta}_{4\to9} \rrbracket_{\{1\}}$	0.2	0.001 (0.019)	0.142 (0.125)
$\llbracket \boldsymbol{\beta}_{1\to4} \rrbracket_{\{2\}}$	0.3	-0.002 (0.009)	-0.268 (0.001)	$\llbracket \boldsymbol{\beta}_{4\to9} \rrbracket_{\{2\}}$	0.2	0.003 (0.017)	-0.012 (0.022)
$\llbracket \boldsymbol{\beta}_{1\to4} \rrbracket_{\{3\}}$	0.5	0.005 (0.009)	-0.446 (0.001)	$\llbracket \boldsymbol{\beta}_{4\to9} \rrbracket_{\{3\}}$	0.3	0.165 (0.130)	-0.260 (0.015)
$\llbracket \boldsymbol{\beta}_{1\to9} \rrbracket_{\{1\}}$	0.2	-0.032 (0.011)	-0.017 (0.009)	$\llbracket \boldsymbol{\beta}_{4\to9} \rrbracket_{\{4\}}$	0.3	0.119 (0.138)	-0.266 (0.010)
$\llbracket \boldsymbol{\beta}_{1\to9} \rrbracket_{\{2\}}$	0.3	-0.056 (0.043)	-0.268 (0.002)	$\llbracket \boldsymbol{\beta}_{5\to8} \rrbracket_{\{1\}}$	0.5	0.031 (0.009)	0.043 (0.009)
$\llbracket \boldsymbol{\beta}_{1\to9} \rrbracket_{\{3\}}$	0.3	-0.077 (0.037)	-0.270 (0.002)	$\llbracket \boldsymbol{\beta}_{5\to8} \rrbracket_{\{2\}}$	0.2	0.006 (0.009)	0.016 (0.009)
$\llbracket \boldsymbol{\beta}_{2\to5} \rrbracket_{\{1\}}$	0.5	0.027 (0.006)	0.028 (0.006)	$\llbracket \boldsymbol{\beta}_{5\to8} \rrbracket_{\{3\}}$	0.3	0.021 (0.023)	-0.265 (0.002)
$\llbracket \boldsymbol{\beta}_{2\to5} \rrbracket_{\{2\}}$	0.2	0.004 (0.006)	0.004 (0.006)	$\llbracket \boldsymbol{\beta}_{5\to8} \rrbracket_{\{4\}}$	0.3	-0.004 (0.022)	-0.268 (0.002)
$\llbracket \boldsymbol{\beta}_{2\to5} \rrbracket_{\{3\}}$	0.3	-0.028 (0.014)	-0.270 (0.001)	$\llbracket \boldsymbol{\beta}_{5\to9} \rrbracket_{\{1\}}$	0.2	0.023 (0.011)	-0.014 (0.011)
$\llbracket \boldsymbol{\beta}_{2\to5} \rrbracket_{\{4\}}$	0.3	-0.002 (0.014)	-0.268 (0.001)	$\llbracket \boldsymbol{\beta}_{5\to9} \rrbracket_{\{2\}}$	0.5	0.076 (0.013)	0.067 (0.014)
$\llbracket \boldsymbol{\beta}_{2\to6} \rrbracket_{\{1\}}$	0.2	0.008 (0.008)	0.010 (0.007)	$\llbracket \boldsymbol{\beta}_{5\to9} \rrbracket_{\{3\}}$	0.3	0.105 (0.060)	-0.253 (0.006)
$\llbracket \boldsymbol{\beta}_{2\to6} \rrbracket_{\{2\}}$	0.2	0.006 (0.007)	0.005 (0.007)	$\llbracket \boldsymbol{\beta}_{5\to9} \rrbracket_{\{4\}}$	0.3	0.068 (0.031)	-0.263 (0.003)
$\llbracket \boldsymbol{\beta}_{2\to6} \rrbracket_{\{3\}}$	0.3	-0.009 (0.017)	-0.269 (0.002)	$\llbracket \boldsymbol{\beta}_{6\to8} \rrbracket_{\{1\}}$	0.2	0.024 (0.011)	0.033 (0.011)
$\llbracket \boldsymbol{\beta}_{2\to6} \rrbracket_{\{4\}}$	0.3	0.035 (0.018)	-0.264 (0.002)	$\llbracket \boldsymbol{\beta}_{6\to8} \rrbracket_{\{2\}}$	0.2	0.022 (0.011)	0.029 (0.011)
$\llbracket \boldsymbol{\beta}_{2\to9} \rrbracket_{\{1\}}$	0.2	0.000 (0.015)	0.005 (0.027)	$\llbracket \boldsymbol{\beta}_{6\to8} \rrbracket_{\{3\}}$	0.3	0.062 (0.026)	-0.261 (0.003)
$\llbracket \boldsymbol{\beta}_{2\to9} \rrbracket_{\{2\}}$	0.2	0.030 (0.014)	0.022 (0.013)	$\llbracket \boldsymbol{\beta}_{6\to8} \rrbracket_{\{4\}}$	0.5	0.090 (0.027)	-0.437 (0.003)
$\llbracket \boldsymbol{\beta}_{2\to9} \rrbracket_{\{3\}}$	0.3	0.197 (0.104)	-0.245 (0.012)	$\llbracket \boldsymbol{\beta}_{6\to9} \rrbracket_{\{1\}}$	0.2	0.013 (0.017)	-0.007 (0.017)
$\llbracket \boldsymbol{\beta}_{2\to9} \rrbracket_{\{4\}}$	0.3	0.067 (0.050)	-0.262 (0.005)	$\llbracket \boldsymbol{\beta}_{6\to9} \rrbracket_{\{2\}}$	0.5	0.104 (0.018)	0.073 (0.022)
$\llbracket \boldsymbol{\beta}_{3\to5} \rrbracket_{\{1\}}$	0.2	0.005 (0.006)	0.010 (0.006)	$\llbracket \boldsymbol{\beta}_{6\to9} \rrbracket_{\{3\}}$	0.3	0.149 (0.108)	-0.256 (0.008)
$\llbracket \boldsymbol{\beta}_{3\to5} \rrbracket_{\{2\}}$	0.2	0.009 (0.007)	0.016 (0.007)	$\llbracket \boldsymbol{\beta}_{6\to9} \rrbracket_{\{4\}}$	0.3	0.046 (0.101)	-0.233 (0.037)
$\llbracket \boldsymbol{\beta}_{3\to5} \rrbracket_{\{3\}}$	0.3	0.033 (0.016)	-0.265 (0.002)	$\llbracket \boldsymbol{\beta}_{7\to8} \rrbracket_{\{1\}}$	0.2	0.002 (0.011)	0.008 (0.011)
$\llbracket \boldsymbol{\beta}_{3\to5} \rrbracket_{\{4\}}$	0.3	0.011 (0.015)	-0.267 (0.002)	$\llbracket \boldsymbol{\beta}_{7\to8} \rrbracket_{\{2\}}$	0.2	0.025 (0.011)	0.019 (0.010)
$\llbracket \boldsymbol{\beta}_{3\to7} \rrbracket_{\{1\}}$	0.2	0.003 (0.008)	-0.000 (0.008)	$\llbracket \boldsymbol{\beta}_{7\to8} \rrbracket_{\{3\}}$	0.5	0.072 (0.031)	-0.440 (0.003)
$\llbracket \boldsymbol{\beta}_{3\to7} \rrbracket_{\{2\}}$	0.2	0.012 (0.008)	0.014 (0.008)	$\llbracket \boldsymbol{\beta}_{7\to8} \rrbracket_{\{4\}}$	0.3	0.037 (0.027)	-0.264 (0.003)
$\llbracket \boldsymbol{\beta}_{3\to7} \rrbracket_{\{3\}}$	0.5	0.029 (0.020)	-0.443 (0.002)	$\llbracket \boldsymbol{\beta}_{7\to9} \rrbracket_{\{1\}}$	0.2	0.015 (0.021)	0.031 (0.042)
$\llbracket \boldsymbol{\beta}_{3\to7} \rrbracket_{\{4\}}$	0.3	-0.015 (0.020)	-0.269 (0.002)	$\llbracket \boldsymbol{\beta}_{7\to9} \rrbracket_{\{2\}}$	0.5	0.120 (0.021)	-0.140 (0.251)
$\llbracket \boldsymbol{\beta}_{3\to9} \rrbracket_{\{1\}}$	0.2	0.030 (0.014)	0.042 (0.015)	$\llbracket \boldsymbol{\beta}_{7\to9} \rrbracket_{\{3\}}$	0.3	0.347 (0.119)	-0.299 (0.040)
$\llbracket \boldsymbol{\beta}_{3\to9} \rrbracket_{\{2\}}$	0.2	-0.004 (0.014)	-0.019 (0.014)	$\llbracket \boldsymbol{\beta}_{7\to9} \rrbracket_{\{4\}}$	0.3	0.153 (0.081)	-0.233 (0.032)
$\llbracket \boldsymbol{\beta}_{3\to9} \rrbracket_{\{3\}}$	0.3	-0.005 (0.103)	-0.259 (0.009)	$\llbracket \boldsymbol{\beta}_{8\to9} \rrbracket_{\{1\}}$	0.5	0.028 (0.008)	0.025 (0.007)
$\llbracket \boldsymbol{\beta}_{3\to9} \rrbracket_{\{4\}}$	0.3	-0.077 (0.099)	-0.270 (0.008)	$\llbracket \boldsymbol{\beta}_{8\to9} \rrbracket_{\{2\}}$	0.5	0.028 (0.008)	0.035 (0.008)
$\llbracket \boldsymbol{\beta}_{4\to6} \rrbracket_{\{1\}}$	0.2	0.013 (0.008)	0.014 (0.008)	$\llbracket \boldsymbol{\beta}_{8\to9} \rrbracket_{\{3\}}$	0.3	0.009 (0.022)	-0.267 (0.002)
$\llbracket \boldsymbol{\beta}_{4\to6} \rrbracket_{\{2\}}$	0.2	0.002 (0.008)	0.005 (0.008)	$\llbracket \boldsymbol{\beta}_{8\to9} \rrbracket_{\{4\}}$	0.5	0.046 (0.022)	-0.442 (0.002)

Table S7: (Left) estimated $\mathbf{c}$ and σ_k^2 in Simulation 4. (Right) estimated $\mathbf{\Sigma}_a$ in Simulation 4. Numbers in the parentheses are the true values.

Parameter	Estimate (truth)
$[\![\mathbf{c}]\!]_{\{1\}}$	0.0016 (0.00)
$[\![\mathbf{c}]\!]_{\{2\}}$	-0.0023 (0.00)
$[\![\mathbf{c}]\!]_{\{3\}}$	-0.0011 (0.00)
$[\![\mathbf{c}]\!]_{\{4\}}$	0.0027 (0.00)
$\sigma^2_{\{1\}}$	0.0400 (0.04)
$\sigma^2_{\{2\}}$	0.0400 (0.04)

	Column 1	Column 2	Column 3	Column 4
Row 1	0.9964 (1.00)	0.2003 (0.20)	0.0008 (0.00)	-0.0003 (0.00)
Row 2	0.2003 (0.20)	0.9983 (1.00)	0.0018 (0.00)	0.0026 (0.00)
Row 3	0.0008 (0.00)	0.0018 (0.00)	0.9980 (1.00)	0.2001 (0.20)
Row 4	-0.0003 (0.00)	0.0026 (0.00)	0.2001 (0.20)	1.0005 (1.00)

S8.2 Simulation 4

This simulation study aims to investigate a multistate model with loops as shown in Figure S6. In this example, $N_y = 2$, $N_z = 2$, $N_t = 150$, $\mathbf{c} = \mathbf{0}_{4\times 1}$, $\mathbf{\Sigma}_a = \text{diag}\left\{\begin{pmatrix} 0.5 & 0.1 \\ 0.1 & 0.5 \end{pmatrix}, \begin{pmatrix} 0.5 & 0.1 \\ 0.1 & 0.5 \end{pmatrix}\right\}$, $h_{0,1\to 2}(t) = h_{0,2\to 3}(t) = 0.8$, $h_{0,2\to 1}(t) = h_{0,3\to 2}(t) = 0.3$, $T_{i,j+1} - T_{ij} - 0.04 \sim \text{Gamma}(3, 0.01)$, $t_{ik,j+1} - t_{ikj} - 0.02 \sim \text{Gamma}(3, 0.01)$ and $\text{P}(s \neq s_i(t), s \in S_{ij}) = 0.02$. The estimated $\boldsymbol{\beta}_{s_1\to s_2}$ are presented in Table S8 and the estimated $\mathbf{c}$, σ_k and $\mathbf{\Sigma}_a$ are presented in Table S9.

Table S8: True values of regression coefficients and the biases of estimated regression coefficients in Simulation 4.

Parameter	Truth	Proposed	MSM
$[\![\boldsymbol{\beta}_{1\to2}]\!]_{\{1\}}$	0.4	0.001 (0.003)	-0.010 (0.004)
$[\![\boldsymbol{\beta}_{1\to2}]\!]_{\{2\}}$	0.2	-0.001 (0.003)	-0.007 (0.003)
$[\![\boldsymbol{\beta}_{1\to2}]\!]_{\{3\}}$	0.5	0.006 (0.005)	-0.276 (0.003)
$[\![\boldsymbol{\beta}_{1\to2}]\!]_{\{4\}}$	0.3	0.000 (0.006)	-0.168 (0.003)
$[\![\boldsymbol{\beta}_{2\to1}]\!]_{\{1\}}$	-0.1	-0.009 (0.010)	-0.008 (0.009)
$[\![\boldsymbol{\beta}_{2\to1}]\!]_{\{2\}}$	-0.1	0.006 (0.010)	0.007 (0.010)
$[\![\boldsymbol{\beta}_{2\to1}]\!]_{\{3\}}$	-0.2	-0.016 (0.019)	0.105 (0.008)
$[\![\boldsymbol{\beta}_{2\to1}]\!]_{\{4\}}$	-0.2	-0.033 (0.019)	0.098 (0.008)
$[\![\boldsymbol{\beta}_{2\to3}]\!]_{\{1\}}$	0.2	0.006 (0.005)	0.001 (0.005)
$[\![\boldsymbol{\beta}_{2\to3}]\!]_{\{2\}}$	0.4	0.017 (0.005)	0.007 (0.005)
$[\![\boldsymbol{\beta}_{2\to3}]\!]_{\{3\}}$	0.3	-0.005 (0.009)	-0.170 (0.004)
$[\![\boldsymbol{\beta}_{2\to3}]\!]_{\{4\}}$	0.5	0.012 (0.009)	-0.274 (0.005)
$[\![\boldsymbol{\beta}_{3\to2}]\!]_{\{1\}}$	-0.1	0.010 (0.021)	0.013 (0.019)
$[\![\boldsymbol{\beta}_{3\to2}]\!]_{\{2\}}$	-0.1	-0.024 (0.023)	-0.012 (0.020)
$[\![\boldsymbol{\beta}_{3\to2}]\!]_{\{3\}}$	-0.2	-0.080 (0.045)	0.088 (0.017)
$[\![\boldsymbol{\beta}_{3\to2}]\!]_{\{4\}}$	-0.2	0.048 (0.040)	0.137 (0.016)

Table S9: (Left) estimated $\mathbf{c}$ and σ_k^2 in Simulation 4. (Right) estimated $\boldsymbol{\Sigma}_a$ in Simulation 4. Numbers in the parentheses are the true values.

Parameter	Estimate (truth)
$[\![\mathbf{c}]\!]_{\{1\}}$	0.0008 (0.00)
$[\![\mathbf{c}]\!]_{\{2\}}$	-0.0016 (0.00)
$[\![\mathbf{c}]\!]_{\{3\}}$	-0.0011 (0.00)
$[\![\mathbf{c}]\!]_{\{4\}}$	0.0026 (0.00)
$\sigma^2_{\{1\}}$	0.0399 (0.04)
$\sigma^2_{\{2\}}$	0.0400 (0.04)

	Column 1	Column 2	Column 3	Column 4
Row 1	0.4982 (0.50)	0.1003 (0.10)	0.0005 (0.00)	-0.0005 (0.00)
Row 2	0.1003 (0.10)	0.4989 (0.50)	0.0004 (0.00)	0.0011 (0.00)
Row 3	0.0005 (0.00)	0.0004 (0.00)	0.4985 (0.50)	0.1009 (0.10)
Row 4	-0.0005 (0.00)	0.0011 (0.00)	0.1009 (0.10)	0.4996 (0.50)

S9 Additional Details on Real Data Application

S9.1 Descriptive summary of TEDDY dataset

The baseline characteristics of the participants in the TEDDY data application are provided in Table S10. The number of observed state transitions is summarized in Table S11.

Table S10: Characteristics and baseline summary of participants in the TEDDY data application.

**Characteristic**	**N = 384**
Age	Median: 5.3, inter-quartile range: 2.5–9.1
DQ2/8 present	Count: 187, frequency: 49%
Glucose	Median: 106, inter-quartile range: 96–119
HbA1c	Median: 5.2, inter-quartile range: 5.0–5.3

Table S11: Observed transition counts in the TEDDY data application (rows: "from" status; columns: "to" status). Symbols denote the positivity of autoantibodies at the statuses (in the order of IA2A, IAA, ZnT8A), and "T1D" denotes the status of T1D diagnosis. −: autoantibody negative, +: autoantibody positive, ?: autoantibody not tested.

**From \To**	− − ?	− − −	− − +	− + ?	− + −	− + +	+ − −	+ − +	+ + ?	+ + −	+ + +	T1D
− − ?	0	0	1	1	0	0	0	0	1	0	0	1
− − −	15	4163	46	4	79	5	33	5	0	7	3	12
− − +	0	0	450	0	0	8	0	23	0	0	1	5
− + ?	0	0	0	7	0	1	0	0	5	0	1	0
− + −	0	0	0	2	541	23	0	0	0	21	5	4
− + +	0	0	0	0	0	264	0	0	0	0	22	6
+ − −	0	0	0	0	0	0	263	12	0	7	2	6
+ − +	0	0	0	0	0	0	0	509	0	0	12	15
+ + ?	0	0	0	0	0	0	0	0	9	0	5	1
+ + −	0	0	0	0	0	0	0	0	1	225	18	14
+ + +	0	0	0	0	0	0	0	0	0	0	1005	48
T1D	0	0	0	0	0	0	0	0	0	0	0	0

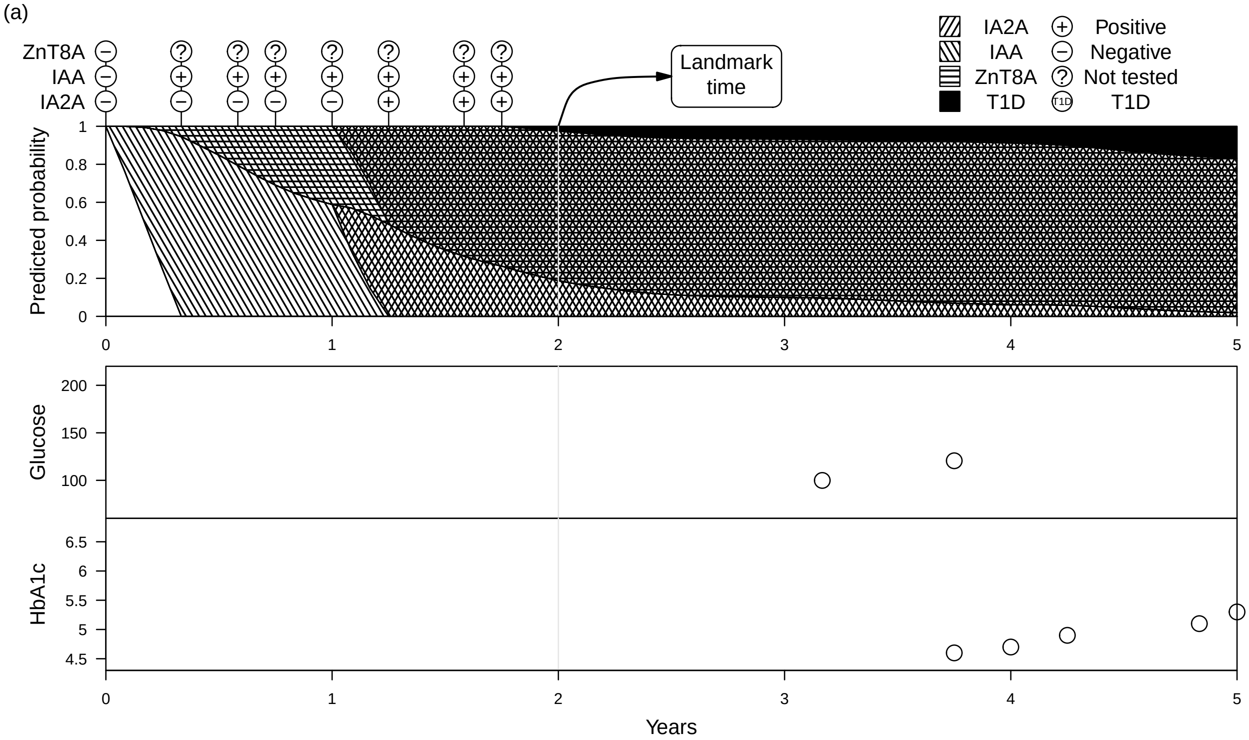

Figure S7: The example in Figure 5(a), with longitudinal measurements added to the graph.

S9.2 Additional information on the results

Figure 5 in the main text showcases three examples demonstrating the proposed dynamic prediction method. In this section, we present the three examples with their longitudinal data in Figures S7–S9.

References

Gu, Y., Zeng, D., Heiss, G., and Lin, D. (2024). Maximum likelihood estimation for semiparametric regression models with interval-censored multistate data. *Biometrika* **111,** 971–988.

Hsieh, F., Tseng, Y.-K., and Wang, J.-L. (2006). Joint modeling of survival and longitudinal data: likelihood approach revisited. *Biometrics* **62,** 1037–1043.

Jäckel, P. (2005). A note on multivariate Gauss-Hermite quadrature. *London: ABN-Amro. Re* .

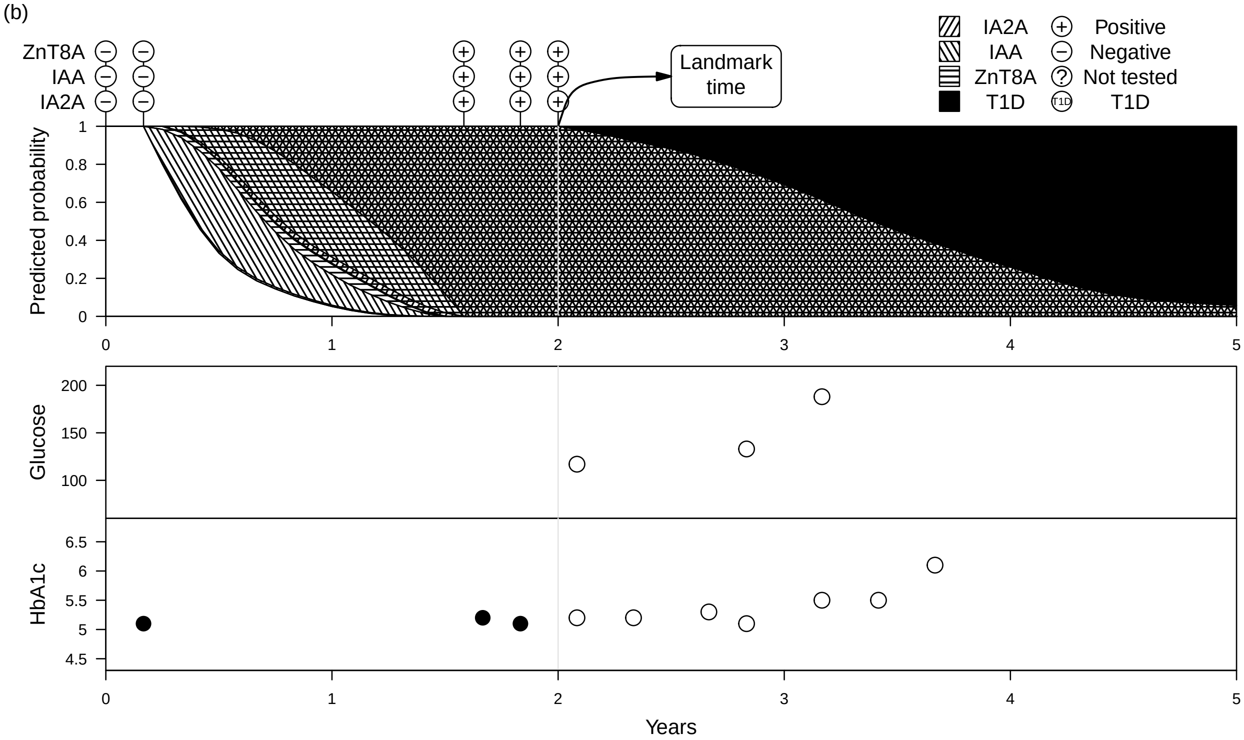

Figure S8: The example in Figure 5(b), with longitudinal measurements added to the graph.

Pullenayegum, E. M. and Lim, L. S. (2016). Longitudinal data subject to irregular observation: A review of methods with a focus on visit processes, assumptions, and study design. *Statistical Methods in Medical Research* **25,** 2992–3014.

Rizopoulos, D. (2012). *Joint Models for Longitudinal and Time-to-Event Data: With Applications in R.* CRC press.

Wulfsohn, M. S. and Tsiatis, A. A. (1997). A joint model for survival and longitudinal data measured with error. *Biometrics* pages 330–339.

You, L., Liu, X., and Krischer, J. (2024). A discrete approximation method for modeling interval-censored multistate data. *Statistics in Medicine* **43,** 2452–2471.

You, L., Salami, F., Törn, C., Lernmark, Å., and Tamura, R. (2024). Joint modeling of multistate and nonparametric multivariate longitudinal data. *The Annals of Applied Statistics* **18,** 2444–2461.

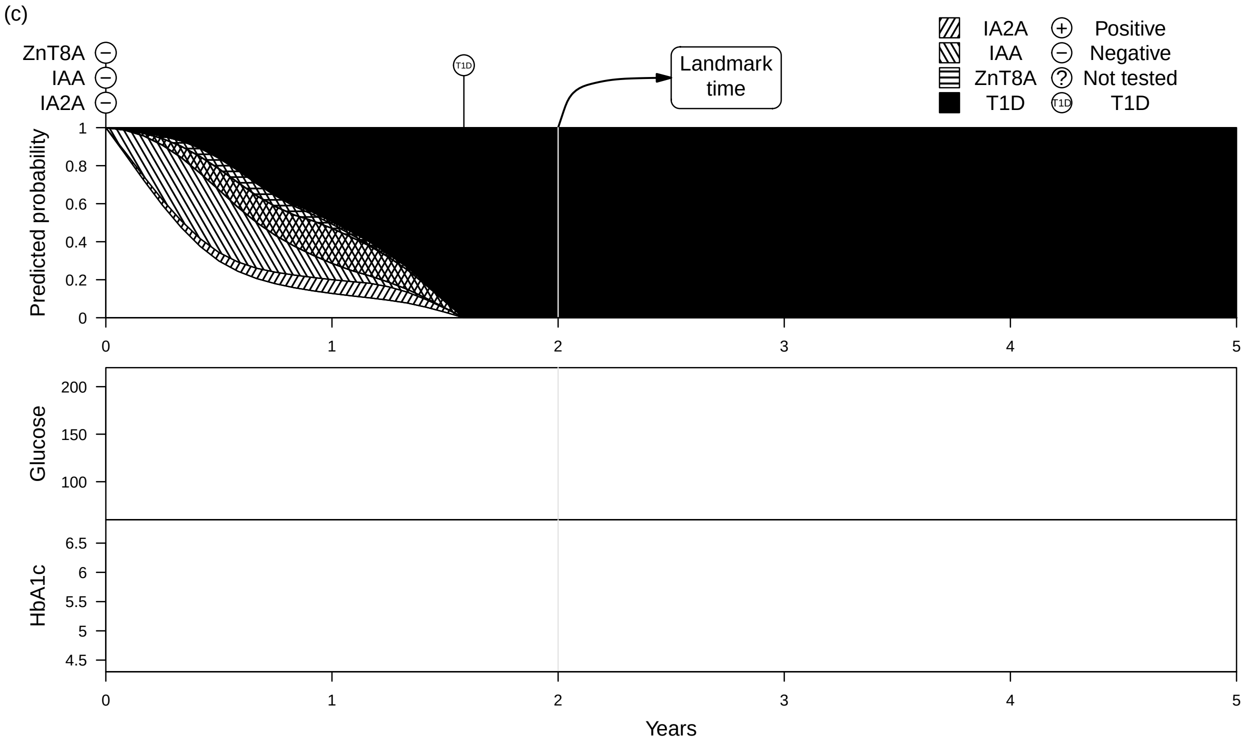

Figure S9: The example in Figure 5(c), with longitudinal measurements added to the graph.
